# Integrated analysis of gut microbiome and fecal metabolome reveals potential non-invasive biomarkers for early-stage silicosis

**DOI:** 10.1128/spectrum.02977-25

**Published:** 2026-02-11

**Authors:** Yiru Qin, Zhiming Hu, Zexian Dong, Jianlin Shen, Ying Han, Jiayun Wu, Yali Lan, Chuifei Zhong, Yushi Ou, Jie Sun, Jianhua Luo, Cong Li, Zhongxiang Gao, Qifeng Wu, Ying Zhang, Lvqin Wen, Xinxiang Qiu, Weihui Liang, Qiying Nong, Ping Wang, Yongshun Huang, Na Zhao

**Affiliations:** 1 Guangdong Province Hospital for Occupational Disease Prevention and Treatment 639460 , Guangzhou, China; 2 School of Public Health, Guangzhou Medical University 26468, Guangzhou, China; 3 School of Public Health, Sun Yat-sen University 26469 , Guangzhou, China; 4 School of Public Health, Southern Medical University 70570, , Guangzhou, China; Children's National Hospital, George Washington University, Washington, DC, USA

**Keywords:** silicosis, gut microbiome, metabolome, early-stage diagnosis, non-invasive biomarker

## Abstract

Silicosis is an irreversible and progressive form of pulmonary fibrosis resulting from inhalation of silica particles, representing a persistent global health concern. Although the gut microbiota has been implicated in chronic lung diseases, its role in silicosis remains largely unexplored. Here, we performed 16S ribosomal RNA (rRNA) gene sequencing on fecal samples from 78 silicosis patients (27 stage I, 24 stage II, 27 stage III) and 30 matched healthy controls (HCs), and further conducted untargeted fecal metabolomics profiling in stage I patients, the critical point for microbial dysbiosis. Silicosis patients exhibited significantly altered beta diversity compared with HCs. At the phylum level, a progressive increase in *Proteobacteria* and a decline in *Bacteroidota* were observed. Notably, *Pantoea, Kluyvera,* and unclassified *Pasteurellaceae* were significantly enriched in stage I patients, with persistent alterations across later stages, suggesting stage I as a key turning point of microbial dysbiosis. Metabolomic analysis of stage I patients revealed distinct profiles enriched in tyrosine, histidine, purine metabolism, and arginine biosynthesis pathways. Correlation analysis identified strong associations between specific taxa and metabolites, and combined microbial–metabolite signatures such as *Lactobacillus* with N-succinyl-2-amino-6-ketopimelate (N-Succinyl-AKP) achieved an area under the curve (AUC) of 0.84 in distinguishing stage I patients from HCs.

**IMPORTANCE:**

This study systematically characterizes gut microbial changes across different stages of silicosis and integrates microbiome–metabolome data specifically in early-stage patients. We demonstrate that stage I is a critical point for gut microbiome alterations and identify microbe–metabolite signatures with diagnostic potential. These findings highlight the gut microbiome–metabolome combination as a promising source of non-invasive biomarkers for the early detection of silicosis.

## INTRODUCTION

Silicosis is an irreversible and progressive form of pulmonary fibrosis resulting from prolonged inhalation of dust containing high concentrations of crystalline silica (1). Despite advances in occupational health, it remains a global public health challenge, particularly in developing countries with large at-risk workforces (2, 3). Between 1990 and 2019, the incidence of silicosis grew by 64.6%, its prevalence rose by 91.4%, and disability-adjusted life years associated with the disease increased by 20.8% globally (4). Although oxidative stress, chronic inflammation, and immune dysregulation have been implicated in its pathogenesis (5–7), the underlying mechanisms remain incompletely understood, and reliable biomarkers for early diagnosis are still lacking.

Emerging evidence reveals the effects of gut microbiota and gut-lung bidirectional communication in the development of pulmonary diseases (8, 9). Several long-term lung disorders have been linked to gut microbiota dysbiosis (10), including asthma (11), chronic obstructive pulmonary disease (12), bronchiectasis (13), idiopathic pulmonary fibrosis (14), and pneumoconiosis (15). Microbe-derived metabolites act as mediators of gut–lung communication, influencing inflammation and tissue remodeling (16, 17). These findings highlight the gut microbiome and its metabolites as potential contributors to lung disease pathogenesis and possible sources of non-invasive biomarkers.

Recent studies suggest that silicosis is also associated with alterations in gut microbiota, both in humans and in animal models. Patients with silicosis exhibit reduced microbial diversity compared with healthy controls (HCs), while experimental models have demonstrated significant shifts in microbial composition and amino acid metabolism following silica exposure (18–20). However, critical gaps remain. First, the stage-specific alterations of gut microbiota across the clinical progression of silicosis have not been systematically investigated. Moreover, the interactions between gut microbes and fecal metabolites in silicosis remain poorly understood, limiting our ability to link microbiome changes with functional metabolic consequences. Last but not least, it is unclear whether early-stage silicosis, the critical window for intervention, exhibits distinct microbiome–metabolome signatures that could serve as non-invasive biomarkers for diagnosis.

Culture-based methods are difficult to operate under conventional laboratory conditions for studying obligate anaerobes or fastidious species of intestinal microorganisms (21). For instance, Campylobacter spp. require microaerophilic environments and specialized media to grow, and recent reports have identified highly aerotolerant clinical isolates that further complicate culture-based identification (22). Therefore, high-throughput sequencing of 16S ribosomal RNA (rRNA) genes provides a culture-independent, comprehensive approach to profile the gut microbial community with greater accuracy and sensitivity (23). In this context, we hypothesized that the gut microbiome and its derived metabolites undergo stage-specific dysregulation during the clinical progression of silicosis, and that these changes may serve as potential indicators of disease onset and development. To test this hypothesis, we conducted 16S rRNA gene sequencing of fecal samples from patients across three clinical stages of silicosis and matched HCs, systematically characterizing stage-specific alterations in gut microbial composition. Based on the observation that stage I represents a critical point for microbial dysbiosis, we further performed untargeted fecal metabolomics and integrated microbiome–metabolome analyses in stage I patients. Our findings reveal distinct microbial and metabolic alterations, as well as diagnostic signatures based on combined microbe–metabolite markers, providing new insights into silicosis pathogenesis and offering promising candidates for non-invasive early diagnosis.

## MATERIALS AND METHODS

### Participant enrollment

Approval for this study was granted by the Medical Ethics Committee of Guangdong Province Hospital for Occupational Disease Prevention and Treatment (approval no. GDHOD MEC 2022032). Participants, including HCs and silicosis patients, were recruited from the same hospital. Silicosis patients were diagnosed following the Chinese Diagnostic Criteria for Silicosis (GBZ 70-2015). The control group consisted of HCs who were specifically recruited from the hospital’s routine health examination center, rather than from inpatients or outpatient clinics. All participants met the inclusion criteria and exclusion criteria in Table S1. HCs matched to the patient group by age, sex, body mass index (BMI), smoking status, and alcohol consumption. A standardized questionnaire was used to gather demographic data and possible risk factors, including family histories of pulmonary and gastrointestinal diseases. All participants were male Han Chinese, aged 18 to 65 years, with no specific dietary habits and free from systemic, metabolic, or other pulmonary diseases. The exclusive inclusion of males was intended to minimize the confounding influence of sex hormones or menstrual cycle-related fluctuations on gut microbiota composition and metabolic activity, which are known to differ significantly between genders (24, 25). Moreover, epidemiological data indicate that over 95% of occupationally exposed silicosis cases occur among male workers (26, 27). Both patients and HCs with a BMI ≥35 or ≤18, or those who had received antibiotics, probiotics, or immunosuppressive therapies within the previous 3 months, were excluded.

### Pulmonary function assessments

Pulmonary function assessments were conducted by a qualified physician following standard protocols. The assessed parameters included diffusing capacity for total lung capacity (TLC), forced expiratory volume in one second (FEV1), forced vital capacity (FVC), and carbon monoxide (DLCO). Measured values were compared to the predicted values for DLCO, TLC, FVC, and FEV1, which were automatically determined by the pulmonary function instrument based on the patient’s gender, height, weight, and age. This comparison provided the FVC (% predicted), FEV1 (% predicted), DLCO (% predicted), TLC (% predicted), and the FEV1/FVC (% predicted). All assessments were conducted using a pulmonary function testing device (MIR Spirolab II, Roma, Italy).

### Fecal samples collection, DNA extraction, and 16S rRNA gene sequencing

In total, 108 fecal samples were obtained using disposable sterile forceps in the morning after a fasting period of at least 8 h. All samples were promptly aliquoted and preserved for the subsequent extraction of DNA at −80°C. The DNA of microorganisms was extracted following the protocol provided by the DNA Extraction Kit (Omega Bio-tek, Norcross, USA). DNA quantification was conducted with the NanoDrop 2000 (Thermo Scientific, Waltham, USA). The bacterial 16S rRNA V3–V4 variable region was amplified using PCR with the primers: 341 F (5′-CCTACGGGNGGCWGCAG-3′) and 805R (5′-GACTACHVGGGTATCTAATCC-3′). A total of 12.5 μL of PCR Premix, 25 ng of template DNA, 5 μL of primer mix, and RNase-free double-distilled water were the components of the PCR reaction mixture. Qualified PCR products were utilized for library preparation, and the NovaSeq 6000 platform was used for sequence analysis (LC-Bio, Hangzhou, China).

### Sequencing data processing, contamination control, and microbial taxonomy assignment

Primers and adapter sequences were eliminated from raw sequencing data using Cutadapt (v1.9). Paired-end sequences were combined into longer sequences relying on overlapping regions with FLASH (v1.2.8). To ensure data quality, sequencing reads were processed with Fqtrim (v0.94) to filter out sequences shorter than 100 bp or those with a base quality score below 5% after trimming. Chimeric sequences were detected and eliminated with Vsearch (v2.3.4). High-resolution amplicon sequence variants (ASVs) were inferred using the DADA2 plugin implemented in QIIME2, with length-based filtering applied during the denoising process. This workflow generated an ASV feature table, representative sequences, and ASV abundance matrices.

To control for potential reagent- or environment-derived contamination, a frequency-based decontamination procedure was performed using the decontam R package version 1.17.0. Sample-specific DNA concentrations (Table S5) were quantified using a Qubit fluorometer prior to PCR amplification and used to model the inverse relationship between ASV abundance and DNA concentration. Due to the absence of sequenced negative controls, prevalence-based decontamination was not applied. ASVs identified as potential contaminants were removed at the ASV level prior to downstream analyses. Taxonomic annotation was performed by comparing ASV representative sequences to the SILVA NT-16S database (v.138) with a confidence threshold of 0.7. Alpha diversity, beta diversity, and abundance statistics analyses were performed by the ggplot2 package.

### Microbial function analysis

Differentially abundant taxa across study groups were identified using the linear discriminant analysis (LDA) effect size (LEfSe) algorithm based on the decontaminated ASV-derived taxonomic table. Subsequent correlation analysis was conducted on genera with an LDA score >2.0 to examine their association with lung function parameters. To evaluate the discriminatory capacity of these genera, receiver operating characteristic (ROC) curves were constructed using the pROC package. The phylogenetic investigation of communities by reconstructing unobserved states 2 (PICRUSt2) was used to provide functional predictions of microbial communities. The predicted results were then analyzed and visualized using STAMP (v2.1.3) to identify significantly different Kyoto Encyclopedia of Genes and Genomes (KEGG) pathways and to forecast the functional capabilities of the silicosis microbiota.

### Liquid chromatography-quadrupole time-of-flight mass spectrometry analysis

Sample preparation was based on a previous study (28). Briefly, fecal samples (50 mg) were homogenized with 1 mL of pre-cooled methanol–acetonitrile–water (2:2:1, vol/vol/vol). The mixture was processed by vortexing for 30 s, high-frequency homogenizing at 45 Hz for 4 min, followed by ultrasonication in an ice bath for 30 min. After extraction, the samples were centrifuged at 12,000 rpm for 15 min at 4°C. The resulting supernatant was then directly transferred to the autosampler vials for liquid chromatography-quadrupole time-of-flight mass spectrometry analysis. Quality control (QC) samples were created by combining 10 µL from each sample to assess the stability and reproducibility of the untargeted metabolomics data acquisition process. Samples were analyzed using a G6545 Q-TOF mass spectrometer (Agilent, Santa Clara, USA) coupled with an Agilent 1290 HPLC system (Agilent, Santa Clara, USA), utilizing a modified polar reverse-phase C18 column (Agilent Poroshell 120 EC). The mobile phase included 0.1% formic acid, 0.2% ammonium formate (A), and acetonitrile (B) at a 0.4 mL/min flow rate, employing gradient elution. Data were collected using electrospray ionization in both positive and negative ion modes.

### Data treatment and compound identification in untargeted metabolomics data

All data were collected using Agilent MassHunter Data Acquisition software (vB.09.00) and then processed with Agilent MassHunter Workstation Profinder (v10.0 SP1) and Agilent MassHunter Workstation Mass Profiler Professional (v15.1). This process included peak alignment, noise reduction, normalization, and correction of missing values. Metabolites were further annotated against public databases, including the KEGG, the human metabolome database, lipid maps (v2.3), and METLIN. For missing value imputation, the recommended minimum imputation default setting of MetaboAnalyst 6.0 was utilized. Using principal component analysis (PCA) via SIMCA-P (v14.1), dimension reduction and exploratory analysis of the identified metabolites were performed. Orthogonal partial least-squares discriminant analysis (OPLS-DA) was employed to discern features contributing to group separation. The cataloging of differential metabolites between the two groups was followed by mapping them to their corresponding biochemical pathways using the KEGG database for metabolic enrichment pathway analysis.

### Statistical analysis

Continuous variables were compared using one-way analysis of variance (ANOVA) or the Kruskal-Wallis test, whereas categorical variables were compared using the chi-square test. For microbiota data, the Wilcoxon test was used for differential comparisons between two groups with biological replicates, and the Kruskal-Wallis test was applied for comparisons across multiple groups with biological replicates. PCA based on the unweighted UniFrac index was performed, and *P* values were calculated using permutational multivariate analysis of variance (PERMANOVA). In metabolomics analysis, metabolites with variable importance in projection (VIP) score >1, fold change (FC) >1.5 or <0.67, and a Student’s *t*-test *P* value <0.05 were considered differentially abundant between the two groups. Metabolic enrichment pathway analysis was performed on all identified metabolites using hypergeometric testing. Every statistical test was two-sided, and a *P* < 0.05 was deemed statistically significant. Statistical analyses were conducted using SPSS (v29.0) and R (v4.1.3).

## RESULTS

### Characteristics of the study population

The ultimate research participants comprised 30 HCs and 78 silicosis patients, stratified across three disease stages (27 in stage I, 24 in stage II, and 27 in stage III) (Fig. S1). No significant differences were observed in demographic characteristics, including age, BMI, smoking status, and alcohol consumption across the four groups, ensuring that no confounding variables influenced group distinctions prior to experimental design and sample collection (Table 1). Stage III silicosis patients exhibited significantly lower values for FVC (% predicted), FEV1 (% predicted), and DLCO (% predicted) compared to those in stage I (*P* < 0.01), underlining the progressive nature of the disease (detailed data available in Table S2). 

**TABLE 1:** Characteristics of the enrolled subjects*^f^*^,^*^g^*

	HCs (*n* = 30)	Stage I (*n* = 27)	Stage II (*n* = 24)	Stage III (*n* = 27)	*P* values
Age, mean ± SD, years^*a*^	54.80 ± 2.85	53.67 ± 5.14	54.63 ± 4.91	56.30 ± 5.35	0.221
BMI, mean ± SD, kg/m^2*a*^	23.80 ± 2.61	24.18 ± 2.52	25.05 ± 3.76	22.90 ± 2.60	0.068
Smoking status, *n* (%)*^c^*					0.227
Never smoker	13 (43.3)	17 (63.0)	15 (62.5)	13 (48.1)	
Ever smokers	6 (20.0)	6 (22.2)	7 (29.2)	9 (33.3)	
Current smokers	11 (36.7)	4 (14.8)	2 (8.3)	5 (18.5)	
Alcohol status, *n* (%)^*c*^					0.229
>4 times/month	3 (10.0)	0	0	1 (3.7)	
Never or ≤4 times/month	27 (90.0)	27 (100.0)	24 (100.0)	26 (96.3)	
FVC (% predicted)^*a*^	–^*h*^	89.01 ± 11.74	86.29 ± 14.09	73.59 ± 14.97	<0.001^*d*,*e*^
FEV_1_ (% predicted)^*b*^	–	85.59 ± 12.84	80.81 ± 13.03	53.94 ± 17.94	<0.001^*d*,*e*^
FEV_1_/FVC (% predicted)^*b*^	–	101.09 ± 8.72	96.78 ± 10.01	72.19 ± 17.90	<0.001^*d*,*e*^
DLCO (% predicted)*^a^*	–	80.01 ± 13.61	69.62 ± 28.16	57.64 ± 22.33	0.007^*d*^
TLC (% predicted)^*b*^	–	77.92 ± 11.28	68.88 ± 24.96	87.07 ± 27.34	0.045^*e*^

^*a*^ Statistics were based on one-way ANOVA with LSD multiple comparisons.

^*b*^ Statistics were based on a Kruskal-Wallis *H*-test with Dunn’s multiple comparisons.

^*c*^ Statistics were based on a chi-squared test.

^*d*^ *P *< 0.05 between stage I and III silicosis patients.

^*e*^ *P* < 0.05 between stage II and III silicosis patients.

^*f*^ BMI, body mass index; FEV1, forced expiratory volume in 1 s; FVC, forced vital capacity; DLCO, diffusing capacity for carbon monoxide; TLC, total lung capacity.

^*g*^ Data are presented as *n* (%) or means ± SD.

^*h*^ –, not applicable.

### Altered gut microbial composition at different stages of silicosis patients

**Fig 1:**
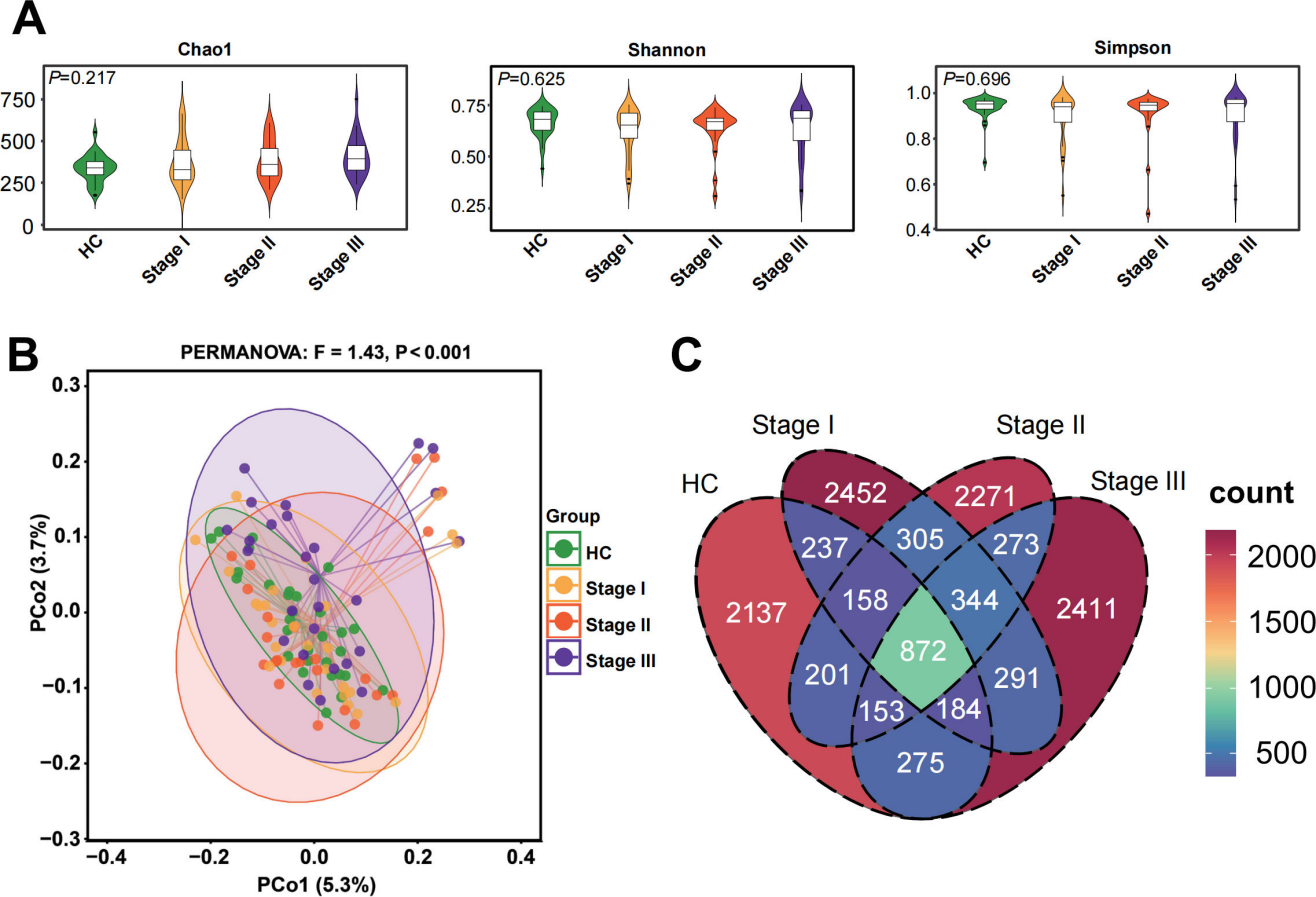
Gut microbiome diversity analysis. (**A**) Alpha diversity indices, including Chao1 index (*P* = 0.217), Shannon index (*P* = 0.625), and Simpson index (*P* = 0.696). (**B**) Beta diversity depicted through unweighted UniFrac analysis using PCoA. (**C**) Venn diagram illustrating the shared ASVs across the four groups.

A total of 68,855,092 high-quality reads were obtained from 16S rRNA gene sequencing, 63,963 reads per sample on average (range: 48,015–75,344). Alpha diversity measures richness and evenness of microbial communities within individuals, while beta diversity reflects differences in microbial community composition between subjects. Analysis of alpha diversity using the Chao1, Shannon, and Simpson indices revealed no significant differences among the four groups (Fig. 1A). In contrast, beta diversity analysis, assessed by principal coordinates analysis (PCoA) based on unweighted UniFrac distance, revealed a notable separation among the four groups (PERMANOVA test, *P* < 0.001; Fig. 1B). Subsequent pairwise comparisons demonstrated significant differences between most group pairs, except between stages I and II silicosis patients (Fig. S2). A Venn diagram depicted that 872 of the total 12,565 ASVs were shared across all groups, while each stage of silicosis exhibited a substantial number of unique ASVs: 2,452 in stage I, 2,271 in stage II, and 2,411 in stage III (Fig. 1C). Despite the absence of notable variations in alpha diversity, distinct alterations in gut microbiome composition occurred across different stages of silicosis, suggesting stage-specific microbial community shifts.

**Fig 2:**
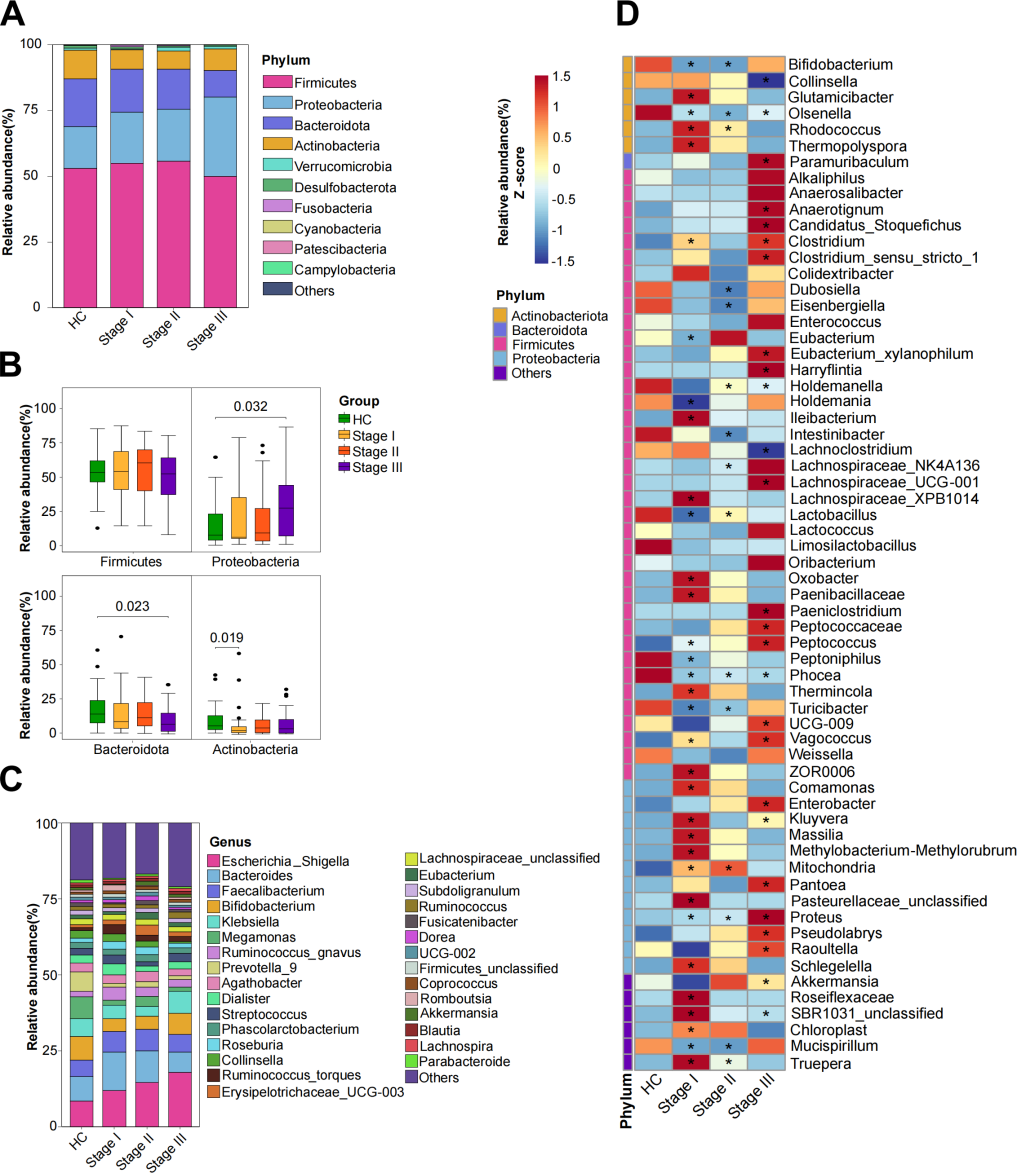
Composition of the gut microbiota. The phylum (**A and B**) and genus (**C**) level of dominant taxa’s relative abundance. (**D**) Heatmap displaying microbial taxa with statistically significant differences between HCs, stages I, II, and III silicosis patients (Wilcoxon test, *P < 0.05).

Compared to HCs, stage III silicosis patients showed a higher abundance of *Proteobacteria* and a lower abundance of *Bacteroidota* at the phylum level. Additionally, a reduction in *Actinobacteria* was noted in stage I silicosis patients compared to the HCs (Fig. 2A and B). No significant differences were found in the *Firmicutes*-to-*Bacteroidetes* (F/B) ratio between the groups (Additional file 1: Fig. S3). At the genus level, fecal microbiota composition varied across groups (Fig. 2C). Specifically, stage III silicosis patients exhibited lower proportions of several beneficial commensal genera, including M*egamonas*, *Prevotella_9*, and *Akkermansia,* compared to HCs. Collectively, microbial composition analysis at various taxonomic levels revealed distinct compositional patterns across different stages of silicosis. We next sought to evaluate stage-specific microbiota patterns in silicosis. Using the Wilcoxon test, we revisited 63 silicosis-associated gut microbes from 761 identified genera (Additional file 2: Table S3), comparing their relative abundances across different stages of silicosis (I, II, and III) and HCs (Fig. 2D). Several genera from the *Firmicutes* phylum, including *Lactobacillus* and *Phocea*, were consistently downregulated in the silicosis groups, while genera from the *Proteobacteria* phylum, such as *Proteus*, were upregulated. Notably, the number of differential microbes, including *Phocea* and *Olsenella*, changed significantly between stage I silicosis patients and HCs, with these alterations persisting in stage II and III. These results suggest that stage I represents a critical point for microbiota compositional changes that may be linked to the progression of silicosis.

### Gut microbiota profiles in early-stage silicosis patients

**Fig 3:**
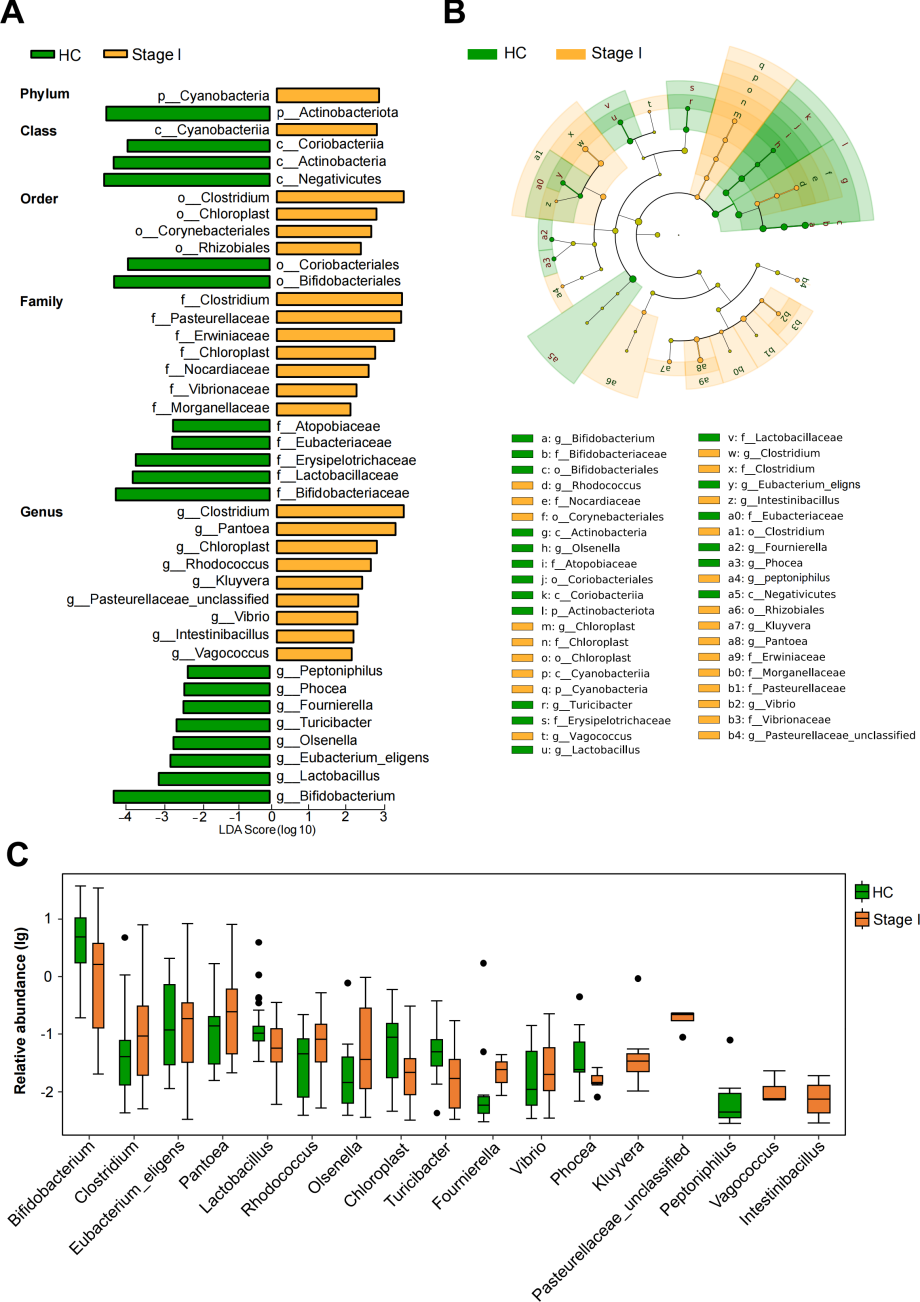
Linear discriminant effect analysis of gut microbiota alterations associated with stage I silicosis patients. (**A**) Cladogram generated from LEfSe analysis, indicating differential abundant bacterial taxa from the phylum to genus levels between HCs and stage I silicosis patients (Wilcoxon test, *P* < 0.05, LDA score > 2). (**B**) Taxonomic cladogram from LEfSe analysis illustrating the distribution of differential gut microbiota identified between HCs and stage I silicosis patients. (**C**) Relative abundance of the top 17 differentially enriched genera between HCs and stage I silicosis patients (Wilcoxon test, *P* < 0.05, LDA score > 2).

To further understand the alterations in gut microbiota of stage I silicosis patients, we identified particular different bacterial taxa that differed between stage I and HCs using a LEfSe multi-level species difference discriminant analysis from phylum to genus level. With an LDA threshold of 2.0, we identified 41 discriminative features across various taxonomic levels: 2 at the phylum level, 4 at the class level, 6 at the order level, 12 at the family level, and 17 at the genus level (Fig. 3A). A cladogram illustrated that nine genera were enriched in stage I, with four belonging to phylum *Proteobacteria* (Fig. 3B). Furthermore, the boxplot depicted the relative abundance of the top 17 genera in stage I patients versus HCs, showing that beneficial genera, such as *Bifidobacterium* and *Lactobacillus,* were more abundant in HCs, while *Proteobacteria* genera, including *Pantoea*, *Kluyvera*, and unclassified *Pasteurellaceae,* were enriched in stage I patients (Fig. 3C).

**Fig 4:**
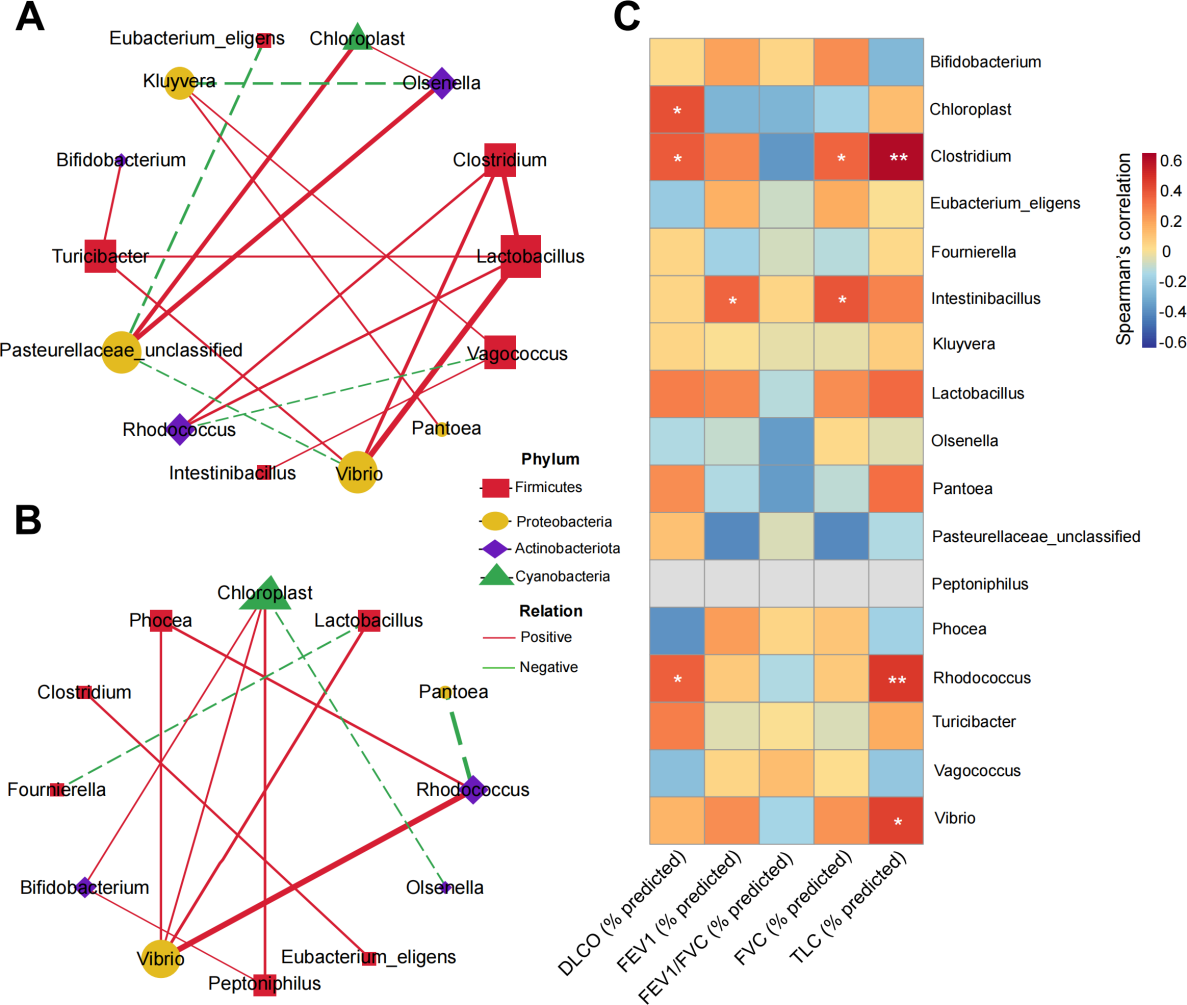
Co-occurrence network of microbes in HCs and stage I silicosis patients. Co-occurrence networks are depicted for the top 17 relatively abundant genera in stage I silicosis patients (**A**) and HCs (**B**). Green edges represent negative correlations (Spearman correlation coefficient < −0.4), while red edges indicate positive correlations (Spearman correlation coefficient > 0.4). The size of each node corresponds to the quantity of related genera, with node colors representing phylum-level classification. (**C**) Heatmap illustrating correlations between 17 differential genera and pulmonary function parameters (Spearman’s correlation analysis, *P < 0.05, **P < 0.01).

Recognizing gut microorganisms as a dynamic, interacting community, we constructed an interaction network among the 17 relatively abundant genera. The gut microbial network in stage I silicosis patients (Fig. 4A) displayed greater complexity and higher connectivity compared to that of HCs (Fig. 4B). Network analysis revealed more frequent interactions among potentially pathogenic bacteria, including unclassified *Pasteurellaceae* and *Vibrio*, in stage I silicosis patients compared to HCs. Conversely, the sparser network in HCs suggested a more stable ecological balance.

We further explored correlations between clinical parameters and individual bacterial taxa among the 17 relatively abundant genera identified in stage I silicosis patients. Notably, several genera showed significant positive correlations with pulmonary function parameters (Fig. 4C). Specifically, *Clostridium* and *Rhodococcus* showed significant positive correlations with DLCO (% predicted) and TLC (% predicted). Additionally, *Intestinibacillus* was significantly positively correlated with FEV1 (% predicted) and FVC (% predicted). To gain insights into the functional alterations in microbial communities between stage I silicosis patients and HCs, we employed PICRUSt2 to predict microbiota functions. This analysis indicated an enrichment of pathways related to fatty acid metabolism and biosynthesis of unsaturated fatty acids in stage I silicosis patients (Fig. S4).

### Metabolic differences in fecal samples between early-stage silicosis patients and HCs

**Fig 5:**
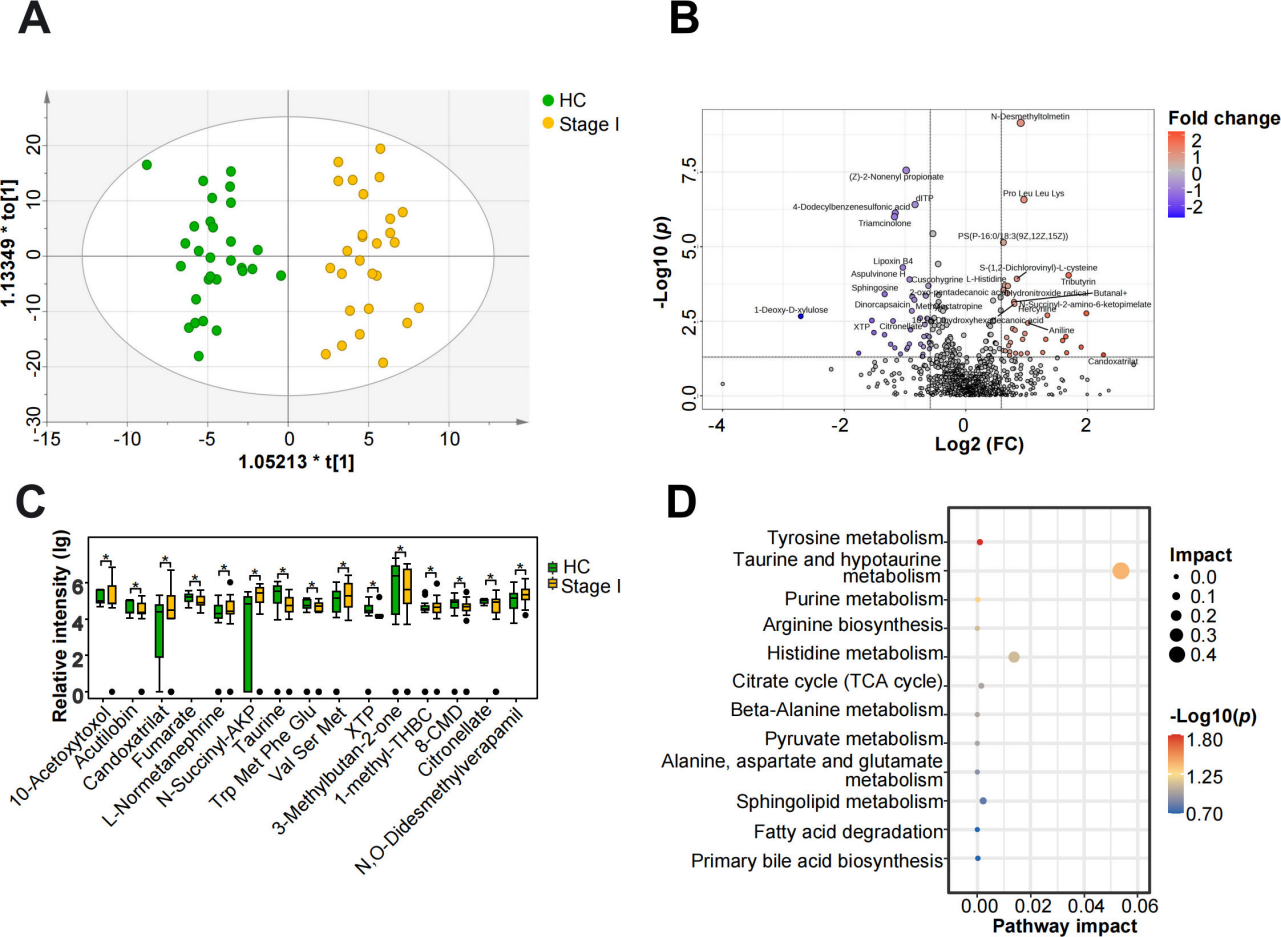
Fecal metabolomics alterations in stage I silicosis patients. (**A**) OPLS-DA score plots derived from untargeted metabolomics data of fecal samples from HCs and stage I silicosis patients. (**B**) Volcano plot visualizing metabolites changes between stage I silicosis patients and HCs (Student’s *t*-test *P* < 0.05, fold change >1.5 or <0.67). (**C**) Boxplot showing the top 15 significantly differential metabolites based on VIP values (*P < 0.05). (**D**) Bubble plot highlighting a significantly enriched pathway.

Considering the potential link between gut microbiota dysbiosis and metabolic changes, we conducted a non-targeted fecal metabolomics analysis of stage I silicosis patients and HCs. The PCA plot demonstrated tight clustering of QC samples, confirming the stability of the analytical process (Fig. S5A and B). A total of 909 metabolites were found, and the OPLS-DA score plot revealed distinct clustering between stage I silicosis patients and HCs, underscoring a clear segregation of metabolites between these two groups (Fig. 5A). The robustness of the OPLS-DA model was further confirmed by the negative Q² intercept from the permutation test (Fig. S5C).

A total of 58 significantly differential metabolites were identified between stage I silicosis patients and HCs, with 25 upregulated and 33 downregulated (Fig. 5B; Table S4). A boxplot illustrated the top 15 significantly differential metabolites based on VIP values between stage I silicosis patients and HCs (Fig. 5C). Compared to HCs, stage I silicosis patients were characterized by elevated levels of L-normetanephrine and N-succinyl-2-amino-6-ketopimelate (N-Succinyl-AKP), while metabolites such as fumarate, xanthosine 5′-triphosphate, and taurine were reduced. These differential metabolites were primarily enriched in pathways related to tyrosine, histidine, purine, taurine, and hypotaurine metabolism, as well as arginine biosynthesis (Fig. 5D).

### Correlations between gut microbiota and metabolites

**Fig 6:**
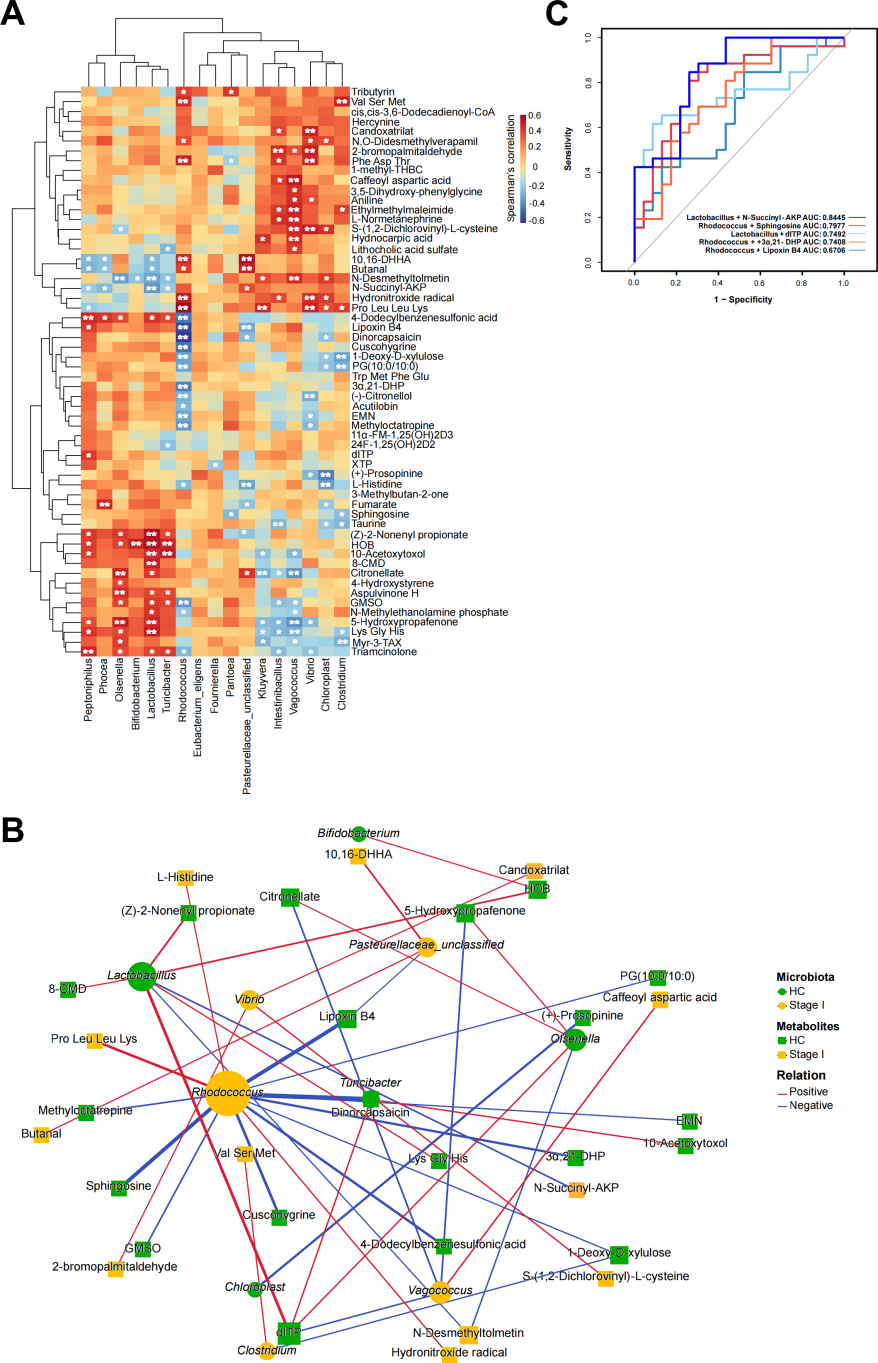
Correlations between microbiota and metabolites, and disease identification based on their combinations. (**A**) Heatmap of Spearman correlations between 17 significantly abundant genera and 58 significantly altered metabolites (*P < 0.05, **P < 0.01). (**B**) Network diagram illustrating microbiota–metabolite interactions based on 41 significant microbiota–metabolite pairs (*P* < 0.05, |r| > 0.4). The correlation coefficient is proportional to the thickness of the edges, while the size of the nodes is correlated with the number of related genera or metabolites. Red denotes a positive link, and blue denotes a negative correlation. (**C**) ROC curve depicting the diagnostic potential of the combined microbiota and metabolites.

To explore the connection between gut microbiota composition and metabolism in early-stage silicosis, Spearman correlation analysis was conducted between 17 significantly abundant genera and 58 metabolites, revealing 169 significant associations (Fig. 6A, *P* < 0.05). A heatmap was created to display the correlation patterns between genera and metabolites. Both positive and negative correlations were observed, involving taxa and metabolites enriched in either stage I silicosis patients or HCs. For instance, genera such as unclassified *Pasteurellaceae* and *Pantoea*, which were more abundant in stage I silicosis patients, exhibited negative correlations with L-Histidine and sphingosine, but positive correlations with 10,16-dihydroxyhexadecanoic acid and butanal. Additionally, fumarate correlated positively with *Phocea*, which was decreased in stage I silicosis patients.

A network of 41 significant gut microbiota–metabolite pairs (*P* < 0.05, |r| > 0.4) was constructed, centering around *Rhodococcus*, *Vibrio*, and *Lactobacillus* (Fig. 6B). This network was closely associated with key metabolites such as 2′-Deoxyinosine-5′-triphosphate (dITP), sphingosine, and 5-hydroxypropafenone. *Rhodococcus*, which was significantly enriched in stage I silicosis patients, exhibited negative correlations with sphingosine and Lipoxin B4, while *Lactobacillus* was positively correlated with dITP. Moreover, combinations of these bacterial taxa and metabolites demonstrated potential for predicting stage I silicosis, such as *Lactobacillus* + N-Succinyl-AKP (area under the curve [AUC]: 0.8445), *Rhodococcus* + sphingosine (AUC: 0.7977), and *Lactobacillus* + dITP (AUC: 0.7492) (Fig. 6C). These findings suggest that the classification model based on microbial–metabolite interactions could serve as a potential diagnostic marker for early-stage silicosis, offering a promising avenue for enhancing diagnostic accuracy and disease monitoring.

## DISCUSSION

The intricate interaction between gut microbiota and host metabolism is essential for both health and disease (8, 29). However, how these changes and interactions manifest in silicosis patients remains unclear. To the best of our understanding, this study is the first to provide a comprehensive characterization of gut microbiota alterations across different clinical stages of silicosis and to explore their functional links to fecal metabolites in early-stage patients, identifying potential non-invasive diagnostic biomarkers.

Although alpha diversity remained comparable among HCs and different stages of silicosis, beta diversity showed significant stage-specific shifts in microbial community structure, indicating distinct gut microbial profiles associated with disease progression. While the F/B ratio remained stable, we noted a progressive decline in *Bacteroidota* alongside an expansion of *Proteobacteria*, a phylum known to produce endotoxins such as lipopolysaccharides (30, 31), suggesting heightened systemic inflammation as silicosis progresses. Concurrently, beneficial SCFA-producing genera, including *Lactobacillus* and *Megamonas*, were reduced, potentially compromising gut homeostasis and contributing to altered host energy and lipid metabolism (32, 33). These coordinated changes suggest that silicosis progression is accompanied by distinct alterations in gut microbial composition, prompting further exploration of disease-associated taxa and their potential functional implications.

Early-stage silicosis was associated with a shift in gut microbial composition, including expansion of potential opportunistic taxa and depletion of beneficial genera, such as *Lactobacillus* and *Megamonas*. The enrichment of *Proteobacteria* members, such as *Kluyvera,* may reflect compromised epithelial barrier integrity, contributing to heightened susceptibility to inflammation and immune activation (34). Correlation analyses suggest that certain gut microbes, including *Clostridium* and *Intestinibacillus,* are linked to pathways relevant to pulmonary immunity. Consistent with previous studies, *Clostridium* has been shown to reduce systemic Th17 cell activity, potentially mitigating IL-17-mediated epithelial-to-mesenchymal transition and fibrotic progression (35–38). *Intestinibacillus*, a propionate-producing genus, may exert protective effects through short-chain fatty acid-mediated modulation of inflammation (39, 40). While these associations do not imply causality, they raise the possibility that gut microbial composition may modulate host immune and inflammatory pathways relevant to lung function and require further mechanistic investigation.

Metabolomic profiling revealed significant changes in fecal metabolites of stage I silicosis patients, particularly in tyrosine, taurine, and hypotaurine metabolism pathways, which have been linked to pulmonary fibrosis pathogenesis (41, 42). A particular finding was the significant reduction of fumarate, a key organic acid involved in regulating redox balance and inflammatory responses (43, 44). Fumarate levels were correlated with *Phocea* and are known to be either utilized or synthesized by various gut bacteria (35, 36, 45, 46). Wang et al. demonstrated that fumarate contributes to idiopathic pulmonary fibrosis through its conversion to succinate (37). L-normetanephrine, a norepinephrine derivative associated with lung diseases due to heightened sympathetic nervous system activity (38, 47, 48), was elevated. Correlations between microbial taxa and these metabolites suggest coordinated changes between gut microbiota and host metabolism. These integrated alterations support that gut microbiota and host metabolism undergo coordinated changes during early silicosis, but more investigation is required to verify the function and causality.

Most studies investigating diagnostic biomarkers for silicosis have predominantly focused on blood markers (49, 50). In contrast, our study highlights the strong discriminative potential of microbiome–metabolite combinations as candidate non-invasive diagnostic biomarkers. Emerging evidence from other chronic pulmonary disorders suggests that gut microbiota–metabolite crosstalk reflects systemic inflammatory and metabolic alterations more sensitively than single-compartment markers (51–53). In this context, our findings support the concept that coordinated changes between specific microbial taxa and host metabolites may encode early disease-related signals in silicosis. Notably, metabolites involved in lipid signaling and nucleotide metabolism, such as sphingosine and purine derivatives, have been implicated in immune modulation and epithelial stress responses in pulmonary diseases (54, 55). Moreover, *Lactobacillus*-associated metabolic profiles have been linked to host metabolic homeostasis in inflammatory conditions (56). Rather than acting as isolated indicators, these microbial–metabolite associations likely represent integrated host–microbe responses to early silica-induced injury.

Several limitations should be noted. The sample size was moderate, and all participants were male Chinese patients, which may restrict generalizability. Although this design minimized confounding by sex hormones and reflects the epidemiology of silicosis, the absence of female participants precludes evaluation of sex-specific microbial or metabolic responses. In addition, HCs were recruited from a hospital-based health examination center rather than the general community, which may introduce bias related to healthcare exposure or lifestyle factors and potentially affect gut microbiota composition and external validity. Crucially, the functional associations between gut microbiota and host metabolism were inferred from Spearman correlations and PICRUSt2-based predictions and should be regarded as exploratory and hypothesis-generating. Accordingly, further *in vivo* and *in vitro* studies are required to establish causal and mechanistic links between candidate microbes, metabolites, and silicosis pathogenesis. Moreover, multiple statistical comparisons were conducted in the differential analyses of microbial taxa and metabolites. Given the exploratory design and limited sample size, formal false discovery rate correction was not applied, increasing the possibility of false-positive findings. Thus, the reported differential features should be interpreted with caution and considered candidate signals requiring validation in larger, well-powered, and diverse cohorts. Future studies with larger, more diverse cohorts are warranted to confirm the diagnostic utility of these findings.

In summary, our study demonstrates stage-specific alterations of gut microbiota in silicosis and identifies stage I as a critical point of microbial dysbiosis. Integration of microbiome and metabolome analyses in early-stage patients revealed distinct metabolic changes and uncovered microbe–metabolite signatures with potential for non-invasive diagnosis. However, these findings should be interpreted as exploratory and hypothesis-generating, providing mechanistic insights rather than definitive diagnostic criteria. Importantly, these candidate signatures are not intended to be used as stand-alone tools for early detection but should be interpreted in conjunction with established clinical indicators, while their disease specificity and cost-effectiveness require further evaluation in larger, multicenter cohorts and across related pulmonary conditions. These results provide new insights into gut microbiome–metabolome interactions in silicosis and lay the groundwork for developing early diagnostic biomarkers.

## Data Availability

The data sets generated during the current study are available in the NCBI SRA repository under projects PRJNA1171554 and SRP538125.

## SUPPLEMENTAL MATERIAL

Supplemental figures spectrum.02977-25-s0001.docx Figures S1 to S5.

Supplemental tables spectrum.02977-25-s0002.docx Tables S1 to S5.

ASM does not own the copyrights to Supplemental Material that may be linked to, or accessed through, an article. The authors have granted ASM a non-exclusive, world-wide license to publish the Supplemental Material files. Please contact the corresponding author directly for reuse.
