# Supplementary material for: Integrated analysis of gut microbiome and fecal metabolome reveals potential non-invasive biomarkers for early-stage silicosis
Source: Microbiol Spectr. 2026 Feb 11;14(3):e02977-25. doi: 10.1128/spectrum.02977-25 (PMC12955483; doi:10.1128/spectrum.02977-25)
Supplement: Supplemental figures — Figures S1 to S5. [file spectrum.02977-25-s0001.docx]

**ADDITIONAL FILES.**

**TITLE:**

**Integrated analysis of** **gut microbiome and fecal metabolome reveals potential non-invasive biomarkers for early-stage silicosis**

**AUTHORS:**

Yiru Qin^1†^, Zhiming Hu^1,2^^†^, Zexian Dong^1†^, Jianlin Shen^3^, Ying Han^1^, Jiayun Wu^1^, Yali Lan^1,3^, Chuifei Zhong^1^, Yushi Ou^1^, Jie Sun^1^, Jianhua Luo^1^, Cong Li^1^, Zhongxiang, Gao^1^, Qifeng Wu^1^, Ying Zhang^1^, Lvqin Wen^1^, Xinxiang Qiu^1^, Weihui Liang^1^, Qiying Nong^1^, Ping Wang^1^, Yongshun Huang^1^ and Na Zhao^1,4^

Affiliations:

^1^Guangdong Provincial Occupational Disease Prevention and Treatment Hospital, Guangzhou, China.

^2^School of Public Health, Guangzhou Medical University, Guangzhou, China.

^3^School of Public Health, Sun Yat-sen University, Guangzhou, China.

^4^School of Public Health, Southern Medical University, Guangzhou, China.

† Yiru Qin, Zhiming Hu and Zexian Dong are co-first authors of this work.

Corresponding author: Na Zhao, zhaonabmu@126.com

**Additional files 1**

**Fig. S1** Flow chart illustrating the procedures for screening and selection of study participants.

**Fig. S2** Multiple comparisons of beta diversity across study groups.

**Fig. S3** The ratio of Firmicutes/Bacteroidetes among the four groups.

**Fig. S4** Abundant pathways of gut microbiota predicted by PICRUSt2 in HCs and stage I silicosis patients.

**Fig. S5** Untargeted metabolomics data comparison between HCs and stage I silicosis patients.


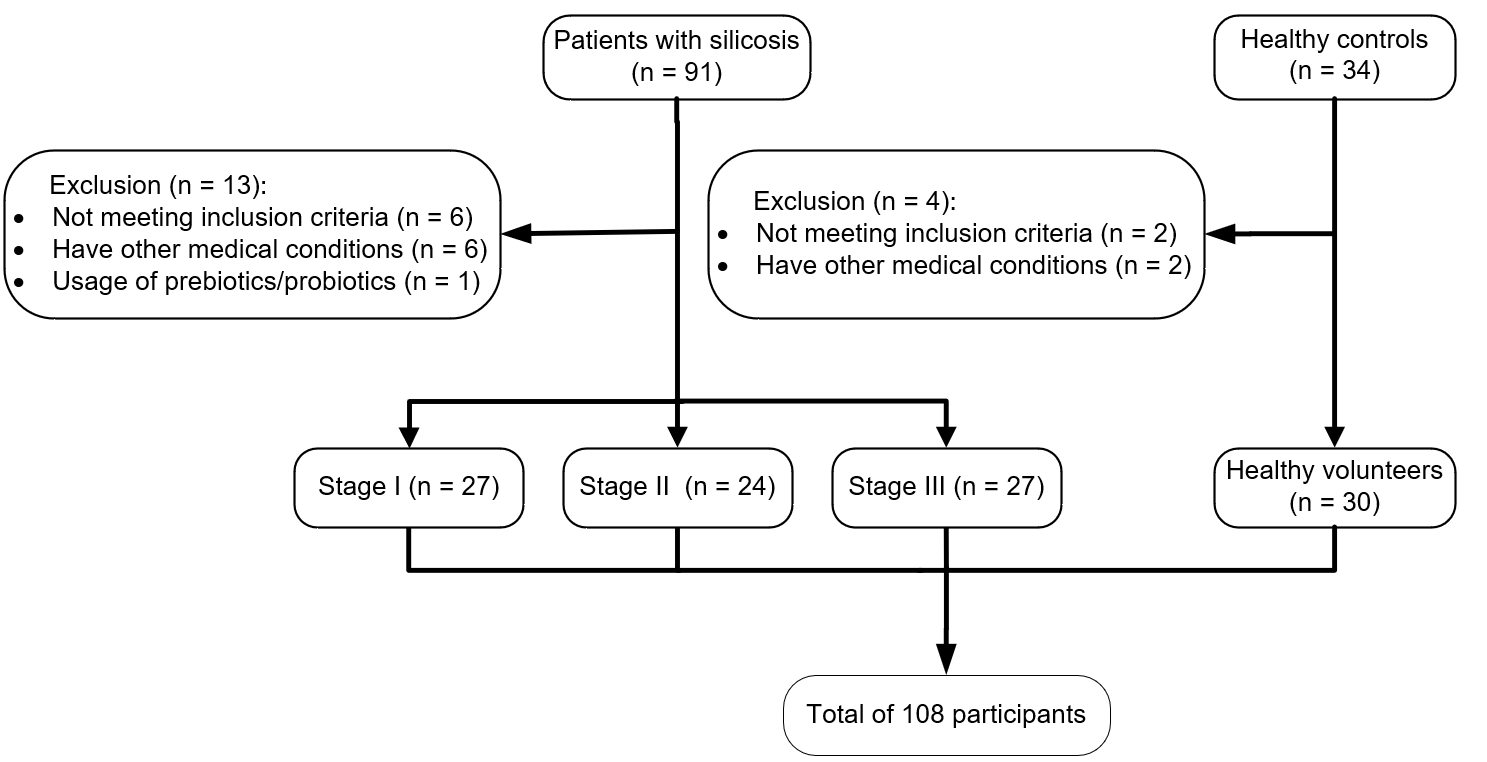


**Fig. S1 Flow chart illustrating the procedures for screening and selection of study participants.**


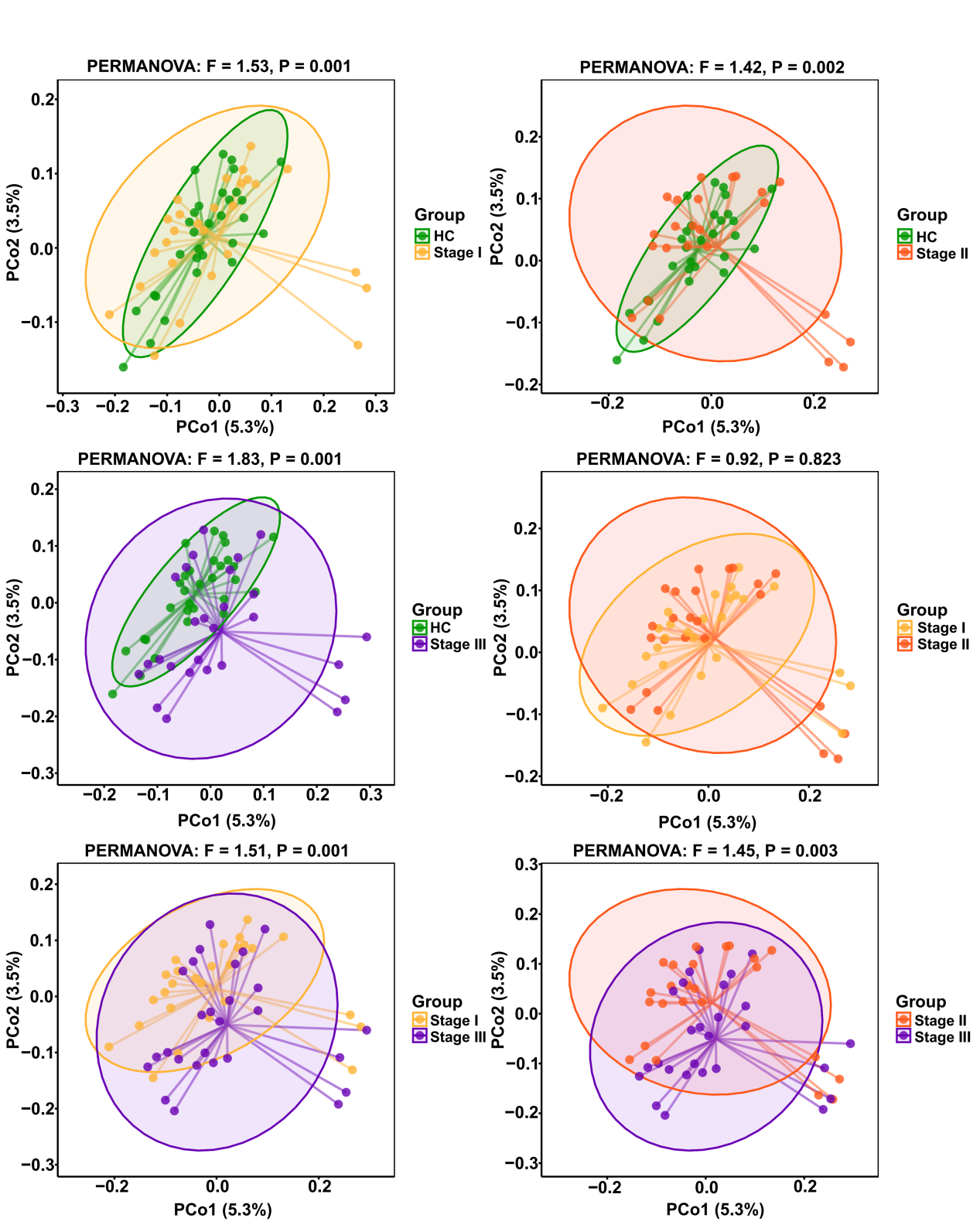


**Fig. S2 Multiple comparisons of beta diversity across study groups.**


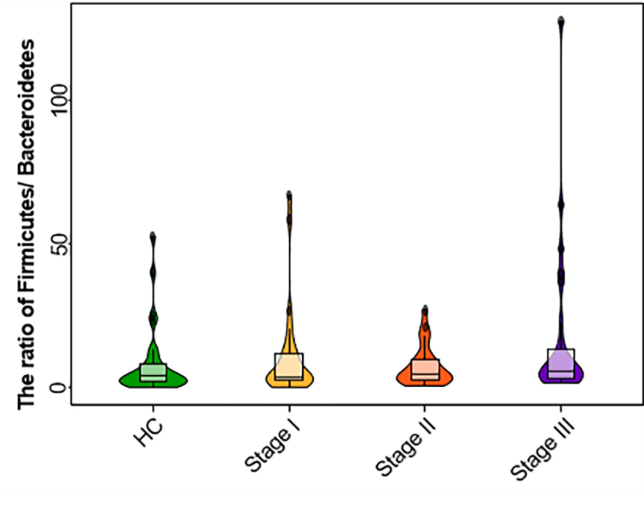


**Fig. S3 The ratio of *Firmicutes*/*Bacteroidetes* among the four groups.**

**
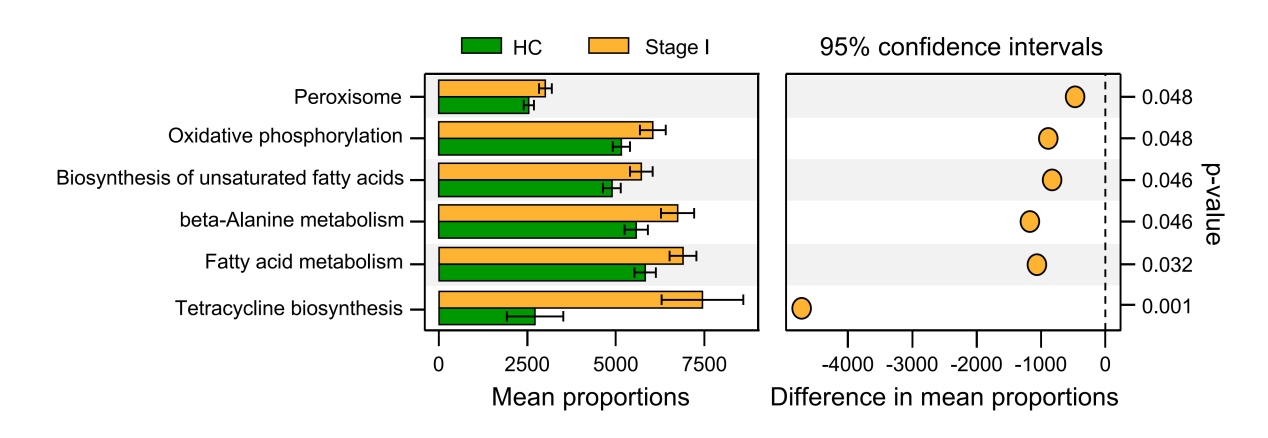
**

**Fig. S4 Abundant pathways of gut microbiota predicted by PICRUSt2 in HCs and stage I silicosis patients.**


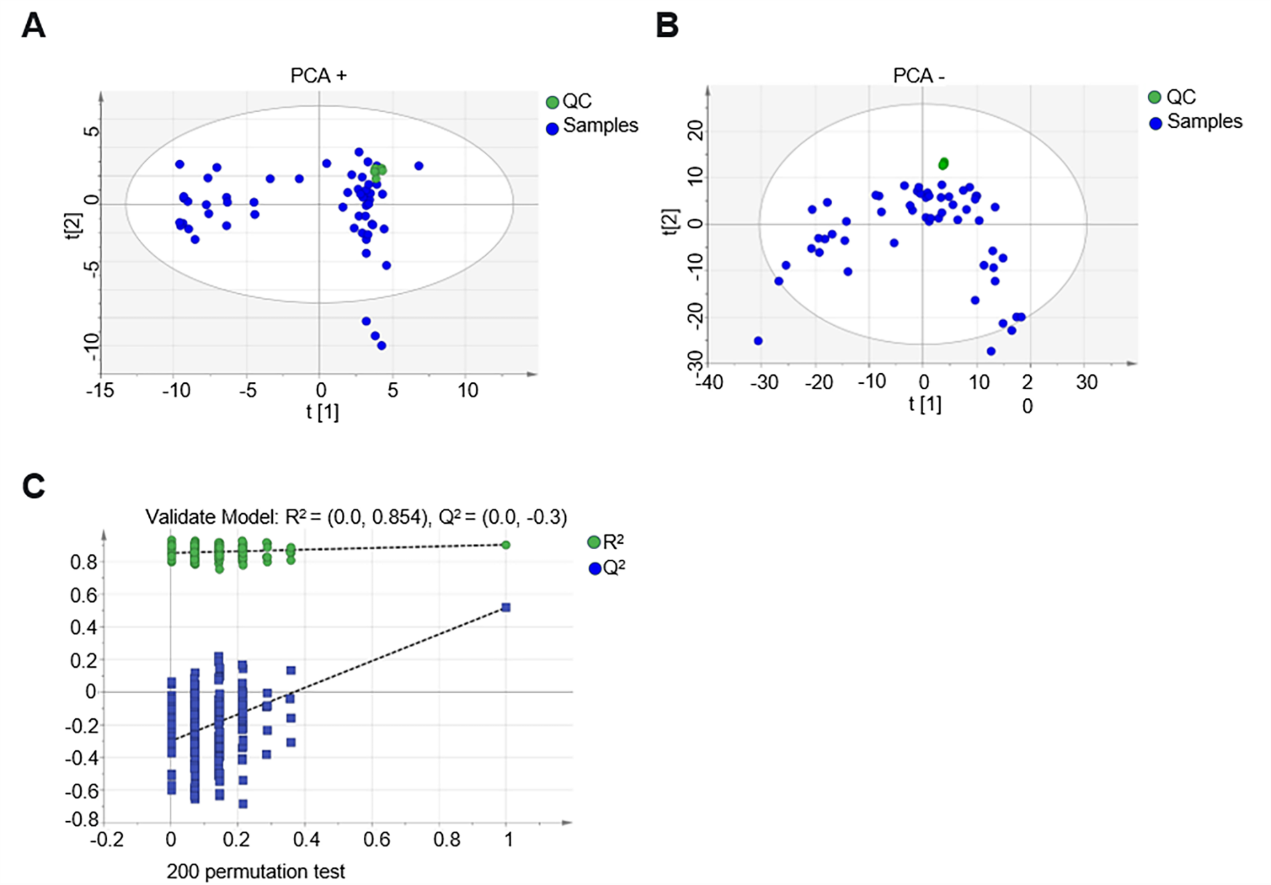


**Fig. S5 Untargeted metabolomics data comparison between HCs and stage I silicosis patients.**

**(A****-B)** PCA score plots of all peak features in positive and negative ion modes from HCs and silicosis stage I. **(C)** OPLS-DA validation plot showing intercepts of R^2^Y = (0.0, 0.854) and Q^2^ = (0.0, -0.3000).
