# Supplementary material for: Integrated analysis of gut microbiome and fecal metabolome reveals potential non-invasive biomarkers for early-stage silicosis
Source: Microbiol Spectr. 2026 Feb 11;14(3):e02977-25. doi: 10.1128/spectrum.02977-25 (PMC12955483; doi:10.1128/spectrum.02977-25)
Supplement: Supplemental tables — Tables S1 to S5. [file spectrum.02977-25-s0002.docx]

| **Table S1.** Inclusion and exclusion criteria for study participation. |
| --- |
| Inclusion criteria: |
| 1. Provision of written informed consent. |
| 2. Male, Han Chinese, aged 18 to 65 years, with no specific dietary habits. |
| 3. Free from any systemic, metabolic, or pulmonary diseases. |
| 4. No use of diets or medications that could affect gut microbiota. |
| 5. HCs without a history of occupational dust exposure. |
| Exclusion criteria: |
| 1. BMI ≥ 35 or ≤ 18. |
| 2. History of alcohol or substance abuse. |
| 3. Acute illness or current evidence of acute or chronic inflammatory or infective diseases. |
| 4. Presence of concomitant lung diseases such as asthma, pulmonary tuberculosis, or malignancies. |
| 5. Use of antibiotics, probiotics, or immunosuppressive therapies within the past three months. |
| 6. Family history of pulmonary or gastrointestinal diseases. |
| 7. History of abdominal or rectal surgery within the past three years. |

**Table S2.** Metadata information of the study population.

| Specimen_name | Age | BMI | Smoking status | Drinking status | Groups | FVC (% predicted) | FEV1 (% predicted) | FEV1/FVC (% predicted) | DLCO (% predicted) | TLC (% predicted) |
| --- | --- | --- | --- | --- | --- | --- | --- | --- | --- | --- |
| S01 | 49 | 24.17 | Ever | Never | Stage I silicosis | 85.00 | 83.00 | 103.00 | 88.00 | 91.00 |
| S02 | 48 | 28.62 | Ever | Never | Stage I silicosis | 87.00 | 81.00 | 96.00 | 97.00 | 83.00 |
| S03 | 58 | 24.68 | Current | Never | Stage I silicosis | 66.00 | 71.00 | 112.00 | 71.00 | 76.00 |
| S04 | 50 | 22.56 | Ever | Never | Stage I silicosis | 100.00 | 97.00 | 100.00 | 70.00 | 65.00 |
| S05 | 58 | 29.63 | Ever | Never | Stage I silicosis | 85.00 | 77.00 | 95.00 | 100.00 | 82.00 |
| S06 | 42 | 21.63 | Ever | Never | Stage I silicosis | 77.00 | 74.00 | 107.00 | 91.00 | 72.00 |
| S07 | 59 | 26.10 | Never | Never | Stage I silicosis | 71.00 | 56.00 | 82.00 | 123.00 | 91.00 |
| S08 | 51 | 26.54 | Never | Never | Stage I silicosis | 97.00 | 94.00 | 101.00 | 91.00 | 88.00 |
| S09 | 56 | 22.83 | Current | ≤4 times/month | Stage I silicosis | 112.00 | 100.00 | 94.00 |  |  |
| S10 | 54 | 25.26 | Never | Never | Stage I silicosis | 109.00 | 103.00 | 103.00 | 92.00 | 84.00 |
| S11 | 54 | 27.59 | Never | Never | Stage I silicosis | 87.00 | 86.00 | 103.00 |  |  |
| S12 | 55 | 22.03 | Never | Never | Stage I silicosis | 94.00 | 100.00 | 112.00 |  |  |
| S13 | 52 | 26.85 | Never | Never | Stage I silicosis | 91.00 | 101.00 | 118.00 | 86.00 | 83.00 |
| S14 | 57 | 20.59 | Never | Never | Stage I silicosis | 106.00 | 103.00 | 102.00 | 90.00 | 87.00 |
| S15 | 55 | 25.32 | Current | Never | Stage I silicosis | 95.00 | 88.00 | 98.00 | 85.00 | 80.00 |
| S16 | 57 | 24.17 | Never | Never | Stage I silicosis | 88.00 | 88.00 | 104.00 | 92.00 | 75.00 |
| S17 | 43 | 23.74 | Never | Never | Stage I silicosis | 93.00 | 90.00 | 101.00 | 79.00 | 78.00 |
| S18 | 57 | 20.35 | Never | Never | Stage I silicosis | 70.00 | 68.00 | 104.00 | 90.00 | 64.00 |
| S19 | 63 | 22.65 | Never | Never | Stage I silicosis | 85.00 | 62.00 | 77.00 | 68.00 | 44.00 |
| S20 | 54 | 24.41 | Never | Never | Stage I silicosis | 72.00 | 70.00 | 102.00 | 81.00 | 64.00 |
| S21 | 60 | 25.31 | Ever | ≤4 times/month | Stage I silicosis | 98.00 | 95.00 | 106.00 | 82.00 | 82.00 |
| S22 | 44 | 19.55 | Never | Never | Stage I silicosis | 90.00 | 85.00 | 97.00 | 72.00 | 88.00 |
| S23 | 56 | 24.28 | Never | Never | Stage I silicosis | 89.00 | 80.00 | 98.00 | 72.00 | 72.00 |
| S24 | 57 | 26.25 | Never | Never | Stage I silicosis | 81.00 | 90.00 | 116.00 | 64.00 | 71.00 |
| S25 | 51 | 22.14 | Never | Never | Stage I silicosis | 81.00 | 80.00 | 103.00 |  |  |
| S26 | 56 | 24.31 | Current | ≤4 times/month | Stage I silicosis | 92.00 | 90.00 | 101.00 | 75.00 | 72.00 |
| S27 | 53 | 21.31 | Never | Never | Stage I silicosis | 102.30 | 98.80 | 94.50 | 52.30 | 104.30 |
| S28 | 48 | 23.65 | Never | Never | Stage II silicosis | 85.00 | 82.00 | 102.00 |  |  |
| S29 | 58 | 28.36 | Never | ≤4 times/month | Stage II silicosis | 76.00 | 86.00 | 89.30 | 104.00 | 65.00 |
| S30 | 58 | 29.96 | Never | Never | Stage II silicosis | 98.00 | 80.00 | 87.00 | 73.00 | 84.00 |
| S31 | 49 | 22.11 | Never | Never | Stage II silicosis | 102.00 | 100.00 | 105.00 |  |  |
| S32 | 68 | 22.18 | Ever | Never | Stage II silicosis | 64.00 | 63.00 | 102.00 | 52.00 | 50.00 |
| S33 | 55 | 29.02 | Never | Never | Stage II silicosis | 84.00 | 86.00 | 107.00 | 80.00 | 66.00 |
| S34 | 55 | 23.88 | Ever | Never | Stage II silicosis | 66.00 | 66.00 | 104.00 |  |  |
| S35 | 46 | 14.52 | Never | Never | Stage II silicosis | 78.00 | 75.00 | 104.00 |  |  |
| S36 | 53 | 23.39 | Current | Never | Stage II silicosis | 108.00 | 75.00 | 74.00 | 78.00 | 96.00 |
| S37 | 57 | 27.04 | Never | Never | Stage II silicosis | 83.00 | 79.00 | 99.00 | 54.00 | 56.00 |
| S38 | 51 | 32.15 | Never | Never | Stage II silicosis | 112.00 | 110.00 | 103.00 | 88.00 | 91.00 |
| S39 | 56 | 24.01 | Never | Never | Stage II silicosis | 94.00 | 91.00 | 101.00 | 64.00 | 58.00 |
| S40 | 48 | 28.27 | Never | Never | Stage II silicosis | 90.00 | 87.00 | 101.00 | 128.00 | 81.00 |
| S41 | 54 | 28.09 | Ever | ≤4 times/month | Stage II silicosis | 84.00 | 88.00 | 109.00 | 95.00 | 78.00 |
| S42 | 58 | 25.91 | Ever | Never | Stage II silicosis | 89.00 | 78.00 | 94.00 | 70.00 | 73.00 |
| S43 | 56 | 29.39 | Ever | Never | Stage II silicosis | 86.00 | 80.00 | 96.00 |  |  |
| S44 | 57 | 22.32 | Never | Never | Stage II silicosis | 61.00 | 57.00 | 96.00 | 53.00 | 45.00 |
| S45 | 59 | 25.26 | Current | Never | Stage II silicosis | 74.00 | 73.00 | 102.00 | 52.00 | 54.00 |
| S46 | 52 | 19.27 | Ever | Never | Stage II silicosis | 100.00 | 88.00 | 91.00 | 87.00 | 89.00 |
| S47 | 59 | 25.88 | Never | Never | Stage II silicosis | 90.00 | 94.00 | 109.00 | 76.00 | 71.00 |
| S48 | 52 | 23.74 | Never | Never | Stage II silicosis | 90.00 | 84.00 | 98.00 | 70.00 | 85.00 |
| S49 | 49 | 24.74 | Ever | Never | Stage II silicosis | 86.00 | 81.00 | 97.00 |  |  |
| S50 | 53 | 24.74 | Never | Never | Stage II silicosis | 64.80 | 50.40 | 75.20 |  |  |
| S51 | 60 | 23.28 | Never | Never | Stage II silicosis | 106.20 | 86.10 | 77.10 | 74.80 | 117.80 |
| S52 | 58 | 23.40 | Never | Never | Stage III silicosis | 80.00 | 63.00 | 83.00 | 77.00 | 61.00 |
| S53 | 51 | 23.21 | Never | Never | Stage III silicosis | 103.00 | 80.00 | 81.00 | 71.00 | 75.00 |
| S54 | 53 | 25.82 | Never | Never | Stage III silicosis | 78.00 | 72.00 | 98.00 |  |  |
| S55 | 54 | 18.56 | Current | Never | Stage III silicosis | 72.00 | 58.00 | 84.00 | 52.00 | 60.00 |
| S56 | 52 | 26.99 | Never | Never | Stage III silicosis | 65.00 | 63.00 | 102.00 | 57.00 | 54.00 |
| S57 | 67 | 24.54 | Ever | Never | Stage III silicosis | 80.00 | 70.00 | 91.00 | 121.00 | 83.00 |
| S58 | 51 | 22.58 | Ever | Never | Stage III silicosis | 92.00 | 83.00 | 94.00 |  |  |
| S59 | 57 | 23.76 | Never | Never | Stage III silicosis | 62.00 | 45.00 | 75.00 | 50.00 | 60.00 |
| S60 | 52 | 20.03 | Never | Never | Stage III silicosis | 52.00 | 36.00 | 71.00 | 56.00 | 53.00 |
| S61 | 56 | 25.53 | Never | Never | Stage III silicosis | 81.00 | 68.00 | 87.00 | 43.00 | 68.00 |
| S62 | 61 | 24.46 | Never | Never | Stage III silicosis | 77.00 | 73.00 | 99.00 | 84.00 | 66.00 |
| S63 | 59 | 22.70 | Never | Never | Stage III silicosis | 79.50 | 31.10 | 37.30 | 49.90 | 107.10 |
| S64 | 59 | 28.06 | Current | Never | Stage III silicosis | 100.40 | 71.80 | 67.90 | 71.10 | 102.50 |
| S65 | 48 | 25.02 | Current | Never | Stage III silicosis | 81.70 | 63.00 | 75.20 | 51.50 | 94.70 |
| S66 | 50 | 23.19 | Ever | Never | Stage III silicosis | 75.70 | 43.70 | 56.70 | 53.30 | 89.20 |
| S67 | 60 | 20.65 | Never | Never | Stage III silicosis | 57.50 | 34.80 | 57.90 | 27.80 | 98.30 |
| S68 | 68 | 19.76 | Never | Never | Stage III silicosis | 42.00 | 24.60 | 54.40 |  |  |
| S69 | 56 | 22.65 | Never | Never | Stage III silicosis | 73.50 | 54.00 | 72.00 |  |  |
| S70 | 58 | 22.95 | Ever | Never | Stage III silicosis | 83.30 | 59.00 | 67.50 | 40.70 | 90.10 |
| S71 | 57 | 21.26 | Ever | Never | Stage III silicosis | 69.30 | 28.10 | 39.30 | 20.60 | 159.00 |
| S72 | 59 | 18.51 | Ever | Never | Stage III silicosis | 54.70 | 46.80 | 81.80 |  |  |
| S73 | 58 | 21.66 | Ever | Never | Stage III silicosis | 62.90 | 38.50 | 58.50 |  |  |
| S74 | 68 | 19.66 | Ever | Never | Stage III silicosis | 81.30 | 64.50 | 73.40 | 47.80 | 124.10 |
| S75 | 58 | 25.98 | Ever | Never | Stage III silicosis | 90.70 | 77.60 | 81.70 | 75.60 | 110.90 |
| S76 | 56 | 23.50 | Current | ≤4 times/month | Stage III silicosis | 71.40 | 46.50 | 62.50 | 45.80 | 98.50 |
| S77 | 54 | 24.92 | Current | Never | Stage III silicosis | 46.10 | 24.70 | 52.20 |  |  |
| S78 | 66 | 18.86 | Never | > 4 times/month | Stage III silicosis | 74.80 | 36.60 | 45.90 |  |  |
| H01 | 64 | 23.88 | Current | Never | Healthy control |  |  |  |  |  |
| H02 | 64 | 23.44 | Ever | Never | Healthy control |  |  |  |  |  |
| H03 | 50 | 25.10 | Current | Never | Healthy control |  |  |  |  |  |
| H04 | 57 | 27.76 | Never | > 4 times/month | Healthy control |  |  |  |  |  |
| H05 | 59 | 21.11 | Current | Never | Healthy control |  |  |  |  |  |
| H06 | 56 | 24.16 | Current | ≤4 times/month | Healthy control |  |  |  |  |  |
| H07 | 51 | 24.22 | Never | > 4 times/month | Healthy control |  |  |  |  |  |
| H08 | 60 | 21.26 | Current | Never | Healthy control |  |  |  |  |  |
| H09 | 47 | 31.83 | Never | ≤4 times/month | Healthy control |  |  |  |  |  |
| H10 | 60 | 21.26 | Never | Never | Healthy control |  |  |  |  |  |
| H11 | 53 | 25.65 | Ever | Never | Healthy control |  |  |  |  |  |
| H12 | 58 | 22.99 | Never | Never | Healthy control |  |  |  |  |  |
| H13 | 56 | 26.57 | Ever | > 4 times/month | Healthy control |  |  |  |  |  |
| H14 | 58 | 24.51 | Ever | Never | Healthy control |  |  |  |  |  |
| H15 | 57 | 21.45 | Current | Never | Healthy control |  |  |  |  |  |
| H16 | 56 | 24.49 | Never | Never | Healthy control |  |  |  |  |  |
| H17 | 58 | 23.26 | Current | ≤4 times/month | Healthy control |  |  |  |  |  |
| H18 | 53 | 24.91 | Never | Never | Healthy control |  |  |  |  |  |
| H19 | 44 | 25.22 | Current | Never | Healthy control |  |  |  |  |  |
| H20 | 53 | 21.22 | Current | Never | Healthy control |  |  |  |  |  |
| H21 | 57 | 23.14 | Ever | Never | Healthy control |  |  |  |  |  |
| H22 | 56 | 24.22 | Never | Never | Healthy control |  |  |  |  |  |
| H23 | 56 | 23.81 | Current | ≤4 times/month | Healthy control |  |  |  |  |  |
| H24 | 54 | 18.43 | Never | Never | Healthy control |  |  |  |  |  |
| H25 | 55 | 22.49 | Never | Never | Healthy control |  |  |  |  |  |
| H26 | 49 | 23.88 | Never | Never | Healthy control |  |  |  |  |  |
| H27 | 57 | 23.81 | Never | Never | Healthy control |  |  |  |  |  |
| H28 | 56 | 20.08 | Never | Never | Healthy control |  |  |  |  |  |
| H29 | 60 | 20.76 | Current | ≤4 times/month | Healthy control |  |  |  |  |  |
| H30 | 52 | 22.09 | Ever | Never | Healthy control |  |  |  |  |  |

Table S3. Statistical results of differential genus abundances.

| Genera | Relative abundance (%) | | | | *kruskal.test.p_value* | *wilcox.test.p_value* | | |
| --- | --- | --- | --- | --- | --- | --- | --- | --- |
|  | mean_HCs | mean_Stage I | mean_Stage II | mean_Stage III |  | HCs vs Stage I | HCs vs Stage II | HCs vs Stage III |
| Eubacterium_xylanophilum_group | 0.05 | 0.03 | 0.13 | 0.27 | 0 | 0.18 | 0.19 | 0.00 |
| Harryflintia | 0.00 | 0.00 | 0.00 | 0.01 | 0.00 | 0.41 | 0.30 | 0.00 |
| Roseiflexaceae_unclassified | 0 | 0.01 | 0 | 0 | 0.00 | 0.01 |  | 0.01 |
| Paramuribaculum | 0.00 | 0.01 | 0.00 | 0.01 | 0.00 | 0.55 | 0.19 | 0.01 |
| Turicibacter | 0.06 | 0.02 | 0.02 | 0.05 | 0.00 | 0.00 | 0.001 | 0.02 |
| Pantoea | 0.10 | 0.50 | 0.03 | 0.89 | 0.00 | 0.05 | 0.37 | 0.16 |
| Olsenella | 0.09 | 0.02 | 0.01 | 0.03 | 0.00 | 0.00 | 0.001 | 0.17 |
| Eisenbergiella | 0.02 | 0.00 | 0.00 | 0.01 | 0.00 | 0.10 | 0.017 | 0.01 |
| Mucispirillum | 0.01 | 0.00 | 0.00 | 0.02 | 0.00 | 0.01 | 0.010 | 0.00 |
| Lactobacillus | 0.29 | 0.08 | 0.19 | 0.15 | 0.00 | 0.00 | 0.015 | 0.00 |
| UCG-009 | 0.01 | 0.00 | 0.01 | 0.01 | 0.00 | 0.56 | 0.51 | 0.00 |
| Peptococcaceae_unclassified | 0.01 | 0.00 | 0.02 | 0.02 | 0.00 | 0.26 | 0.65 | 0.00 |
| Akkermansia | 0.82 | 0.31 | 1.35 | 0.99 | 0.00 | 0.37 | 0.40 | 0.00 |
| Weissella | 0.33 | 0.07 | 0.02 | 0.33 | 0.00 | 0.17 | 0.049 | 0.00 |
| Kluyvera | 0 | 0.04 | 0.00 | 0.02 | 0.00 | 0.00 | 0.26 | 0.69 |
| Mitochondria_unclassified | 0.00 | 0.02 | 0.02 | 0.01 | 0.00 | 0.00 | 0.004 | 0.03 |
| Candidatus_Stoquefichus | 0.00 | 0.00 | 0.00 | 0.00 | 0.00 | 0.81 | 0.71 | 0.01 |
| Chloroplast_unclassified | 0.03 | 0.13 | 0.14 | 0.02 | 0.00 | 0.00 | 0.10 | 0.00 |
| ZOR0006 | 0 | 0.01 | 0.00 | 0.00 | 0.00 | 0.00 | 0.11 | 0.03 |
| Lachnospiraceae_NK4A136_group | 0.07 | 0.07 | 0.08 | 0.15 | 0.00 | 0.14 | 0.006 | 0.02 |
| Paenibacillaceae_unclassified | 0 | 0.02 | 0.01 | 0 | 0.00 | 0.01 | 0.11 | 0.01 |
| Comamonas | 0 | 0.00 | 0.00 | 0 | 0.00 | 0.01 | 0.11 | 0.01 |
| Clostridium_sensu_stricto_1 | 0.27 | 0.77 | 0.13 | 1.33 | 0.01 | 0.46 | 0.57 | 0.06 |
| Schlegelella | 0 | 0.01 | 0.00 | 0 | 0.01 | 0.01 | 0.11 | 0.01 |
| Thermopolyspora | 0 | 0.01 | 0.01 | 0 | 0.01 | 0.01 | 0.11 | 0.01 |
| Thermincola | 0 | 0.01 | 0.01 | 0 | 0.01 | 0.01 | 0.11 | 0.01 |
| Clostridia_UCG-014_unclassified | 0.24 | 0.73 | 0.37 | 0.98 | 0.01 | 0.03 | 0.12 | 0.23 |
| Paeniclostridium | 0 | 0 | 0 | 0.01 | 0.01 |  |  | 0.04 |
| Pseudolabrys | 0.00 | 0.00 | 0.00 | 0.01 | 0.01 | 0.49 | 0.09 | 0.02 |
| Vagococcus | 0 | 0.00 | 0.00 | 0.00 | 0.01 | 0.01 | 0.11 | 0.30 |
| Phocea | 0.04 | 0.00 | 0.01 | 0.01 | 0.01 | 0.01 | 0.010 | 0.53 |
| Intestinibacter | 0.08 | 0.04 | 0.02 | 0.03 | 0.01 | 0.09 | 0.001 | 0.76 |
| Dubosiella | 0.03 | 0.01 | 0.01 | 0.03 | 0.01 | 0.05 | 0.020 | 0.03 |
| Lachnospiraceae_XPB1014_group | 0 | 0.01 | 0.00 | 0 | 0.01 | 0.01 | 0.11 | 0.02 |
| Truepera | 0 | 0.01 | 0.00 | 0.00 | 0.01 | 0.00 | 0.021 | 0.05 |
| Eubacterium | 0.14 | 0.04 | 0.33 | 0.05 | 0.01 | 0.00 | 0.89 | 0.21 |
| Enterobacter | 0.06 | 0.17 | 0.39 | 0.65 | 0.01 | 0.10 | 0.63 | 0.10 |
| Oxobacter | 0 | 0.00 | 0.00 | 0 | 0.02 | 0.01 | 0.049 | 0.02 |
| Glutamicibacter | 0 | 0.01 | 0.00 | 0.00 | 0.02 | 0.01 | 0.11 | 0.04 |
| Rhodococcus | 0.05 | 0.12 | 0.08 | 0.05 | 0.02 | 0.02 | 0.032 | 0.02 |
| Raoultella | 0.06 | 0.02 | 0.06 | 0.09 | 0.02 | 0.31 | 0.98 | 0.01 |
| Holdemanella | 1.01 | 0.17 | 0.55 | 0.46 | 0.02 | 0.07 | 0.008 | 0.66 |
| Pasteurellaceae_unclassified | 0 | 0.04 | 0.00 | 0.00 | 0.02 | 0.01 | 0.26 | 0.07 |
| SBR1031_unclassified | 0 | 0.01 | 0.00 | 0.00 | 0.03 | 0.00 | 0.049 | 0.18 |
| Peptococcus | 0.00 | 0.00 | 0.00 | 0.01 | 0.03 | 0.01 | 0.43 | 0.87 |
| Holdemania | 0.02 | 0.01 | 0.01 | 0.02 | 0.03 | 0.03 | 0.05 | 0.02 |
| Anaerotignum | 0.00 | 0.00 | 0.00 | 0.01 | 0.03 | 0.31 | 0.74 | 0.10 |
| Lachnospiraceae_UCG-001 | 0.02 | 0.01 | 0.02 | 0.07 | 0.03 | 0.07 | 0.18 | 0.26 |
| Lachnoclostridium | 0.56 | 0.59 | 0.46 | 0.30 | 0.04 | 0.99 | 0.85 | 0.03 |
| Bifidobacterium | 7.80 | 4.25 | 4.26 | 7.02 | 0.04 | 0.01 | 0.012 | 0.35 |
| Peptoniphilus | 0.00 | 0 | 0.00 | 0.00 | 0.04 | 0.02 | 0.25 | 0.32 |
| Methylobacterium-Methylorubrum | 0.00 | 0.01 | 0.00 | 0.00 | 0.04 | 0.01 | 0.19 | 0.06 |
| Proteus | 0 | 0.01 | 0.02 | 0.13 | 0.04 | 0.01 | 0.009 | 0.53 |
| Ileibacterium | 0 | 0.00 | 0.00 | 0.00 | 0.04 | 0.01 | 0.11 | 0.20 |
| Collinsella | 2.53 | 2.56 | 2.02 | 0.54 | 0.04 | 0.07 | 0.97 | 0.76 |
| Massilia | 0 | 0.00 | 0.00 | 0 | 0.04 | 0.03 | 0.11 | 0.04 |
| Alkaliphilus | 0.00 | 0 | 0.00 | 0.02 | 0.00 | 0.05 | 0.24 | 0.06 |
| Anaerosalibacter | 0.00 | 0 | 0 | 0.01 | 0.01 | 0.34 | 0.37 | 0.05 |
| Colidextribacter | 0.05 | 0.08 | 0.04 | 0.06 | 0.02 | 0.16 | 0.10 | 0.10 |
| Enterococcus | 0.10 | 0.05 | 0.03 | 0.26 | 0.02 | 0.46 | 0.28 | 0.06 |
| Lactococcus | 0.01 | 0.00 | 0.00 | 0.02 | 0.01 | 0.32 | 0.16 | 0.08 |
| Limosilactobacillus | 0.65 | 0.02 | 0.10 | 0.11 | 0.03 | 0.37 | 0.93 | 0.11 |
| Oribacterium | 0.00 | 0.00 | 0.00 | 0.00 | 0.01 | 0.08 | 0.23 | 0.11 |

**Table S4.** Nine hundred and nine metabolites identified from stage I silicosis patients and HCs

| Metabolites | mean_HCs | mean_Stage I | FC | Log2FC | *p*-values | VIP_score |
| --- | --- | --- | --- | --- | --- | --- |
| 3,9-Dihydroxypterocarpan | 0.132 | -0.142 | 0.063 | 3.998 | 0.400 | 0.687 |
| 1-Deoxy-D-xylulose* | 0.309 | -0.332 | 0.152 | 2.715 | 0.002 | 1.454 |
| (R)-Mevalonate | 0.237 | -0.255 | 0.216 | 2.214 | 0.129 | 1.037 |
| PG(10:0/10:0)* | 0.197 | -0.211 | 0.295 | 1.762 | 0.037 | 1.142 |
| Urate | 0.126 | -0.135 | 0.303 | 1.722 | 0.199 | 0.724 |
| Anhydroglycinol | 0.080 | -0.086 | 0.330 | 1.598 | 0.515 | 0.939 |
| PC(18:2(2E,4E)/0:0) | 0.237 | -0.254 | 0.334 | 1.583 | 0.180 | 1.208 |
| XTP* | 0.327 | -0.351 | 0.343 | 1.545 | 0.003 | 2.015 |
| Sucralose | 0.187 | -0.201 | 0.348 | 1.524 | 0.134 | 0.897 |
| 10-Acetoxytoxol* | 0.300 | -0.322 | 0.351 | 1.512 | 0.008 | 1.907 |
| Methyl salicylate | 0.140 | -0.150 | 0.354 | 1.500 | 0.075 | 0.662 |
| Norgalanthamine | 0.299 | -0.321 | 0.357 | 1.485 | 0.104 | 1.435 |
| Ursolic acid | -0.114 | 0.122 | 0.363 | 1.464 | 0.487 | 0.734 |
| Gibberellin A8 | 0.141 | -0.152 | 0.367 | 1.447 | 0.388 | 1.188 |
| Cortol | 0.236 | -0.254 | 0.386 | 1.372 | 0.158 | 1.387 |
| Acutilobin* | 0.349 | -0.375 | 0.396 | 1.337 | 0.009 | 2.218 |
| Sphingosine* | 0.246 | -0.264 | 0.397 | 1.334 | 0.000 | 1.376 |
| D-threo-3-Methylmalate | 0.155 | -0.167 | 0.400 | 1.324 | 0.066 | 0.664 |
| Tricetin | 0.175 | -0.188 | 0.401 | 1.319 | 0.312 | 1.412 |
| CAY10622 | 0.182 | -0.196 | 0.409 | 1.289 | 0.374 | 1.696 |
| Fumarate* | 0.429 | -0.461 | 0.425 | 1.236 | 0.019 | 1.910 |
| bis(7)-Tacrine | 0.181 | -0.195 | 0.431 | 1.215 | 0.201 | 0.787 |
| 2-(Hydroxymethyl)-4-oxobutanoate* | 0.241 | -0.259 | 0.436 | 1.197 | 0.003 | 1.179 |
| PC(O-14:0/2:0) | 0.022 | -0.023 | 0.441 | 1.182 | 0.862 | 0.674 |
| Picrotoxinin | 0.231 | -0.248 | 0.441 | 1.180 | 0.179 | 1.290 |
| D-Rhamnose | 0.082 | -0.088 | 0.442 | 1.178 | 0.314 | 0.780 |
| PE(18:0/0:0) | 0.097 | -0.104 | 0.442 | 1.177 | 0.488 | 0.731 |
| Triamcinolone* | 0.295 | -0.317 | 0.443 | 1.173 | 0.000 | 1.649 |
| Taurine* | 0.367 | -0.395 | 0.445 | 1.167 | 0.025 | 1.993 |
| 4-Dodecylbenzenesulfonic acid* | 0.280 | -0.301 | 0.447 | 1.161 | 0.000 | 1.431 |
| Arg Asn Lys Phe | 0.050 | -0.053 | 0.448 | 1.158 | 0.762 | 1.080 |
| Met Arg Trp Trp | -0.012 | 0.013 | 0.450 | 1.152 | 0.936 | 0.780 |
| Albanin B | 0.263 | -0.282 | 0.455 | 1.136 | 0.064 | 1.419 |
| M(IP)2C(t20:0/26:0) | 0.020 | -0.022 | 0.458 | 1.125 | 0.845 | 0.239 |
| 20-Eicosanolide | 0.264 | -0.284 | 0.469 | 1.093 | 0.178 | 1.436 |
| Trp Met Phe Glu* | 0.365 | -0.392 | 0.475 | 1.074 | 0.039 | 1.822 |
| PC(2:0/2:0) | 0.203 | -0.218 | 0.483 | 1.049 | 0.270 | 1.506 |
| Lipoxin B4* | 0.252 | -0.271 | 0.488 | 1.036 | 0.000 | 1.171 |
| TMDP | 0.047 | -0.051 | 0.503 | 0.992 | 0.757 | 0.758 |
| (Z)-2-Nonenyl propionate* | 0.289 | -0.311 | 0.507 | 0.980 | 0.000 | 1.249 |
| (+)-Prosopinine* | 0.297 | -0.319 | 0.510 | 0.972 | 0.027 | 1.784 |
| 3-Methylbutan-2-one* | 0.482 | -0.518 | 0.516 | 0.955 | 0.024 | 2.802 |
| (-)-Citronellol* | 0.240 | -0.257 | 0.526 | 0.928 | 0.018 | 1.537 |
| Aspulvinone H* | 0.262 | -0.282 | 0.527 | 0.924 | 0.000 | 1.436 |
| PA(O-16:0/0:0) | 0.252 | -0.271 | 0.533 | 0.909 | 0.064 | 1.292 |
| amifloxacin | 0.233 | -0.251 | 0.533 | 0.908 | 0.006 | 0.950 |
| Dinorcapsaicin* | 0.193 | -0.207 | 0.540 | 0.888 | 0.001 | 1.123 |
| EMN* | 0.238 | -0.255 | 0.544 | 0.879 | 0.000 | 1.485 |
| Muzanzagenin | 0.083 | -0.089 | 0.552 | 0.857 | 0.258 | 0.481 |
| 2-amino-octadecanoic acid | 0.213 | -0.229 | 0.553 | 0.855 | 0.107 | 1.401 |
| Methyloctatropine* | 0.209 | -0.224 | 0.556 | 0.846 | 0.001 | 1.214 |
| dITP* | 0.274 | -0.294 | 0.561 | 0.835 | 0.000 | 1.547 |
| Normorphine | 0.023 | -0.025 | 0.565 | 0.824 | 0.909 | 1.320 |
| 3,4-Dihydroxybenzaldehyde | 0.007 | -0.008 | 0.566 | 0.822 | 0.940 | 0.059 |
| N-Oleoyl-L-Serine | 0.284 | -0.305 | 0.567 | 0.818 | 0.113 | 1.528 |
| 3-Methylxanthine | -0.019 | 0.020 | 0.588 | 0.766 | 0.877 | 0.463 |
| 4-Acetamidobutanoate | 0.244 | -0.262 | 0.590 | 0.762 | 0.159 | 1.234 |
| 2-Methylbutylamine | 0.104 | -0.112 | 0.593 | 0.754 | 0.128 | 0.702 |
| Tetraacetylethylenediamine | 0.005 | -0.005 | 0.593 | 0.753 | 0.972 | 0.473 |
| Citronellate* | 0.430 | -0.461 | 0.594 | 0.751 | 0.003 | 2.298 |
| Esculetin | -0.158 | 0.170 | 0.595 | 0.748 | 0.272 | 1.223 |
| Austroinulin | 0.067 | -0.071 | 0.596 | 0.747 | 0.546 | 0.555 |
| 3α,21-DHP* | 0.170 | -0.183 | 0.598 | 0.741 | 0.019 | 1.025 |
| 3-O-Caffeoyl-4-O-methylquinic acid | -0.052 | 0.055 | 0.600 | 0.737 | 0.775 | 0.843 |
| SM(d18:1/15:0) | 0.153 | -0.164 | 0.600 | 0.736 | 0.182 | 1.189 |
| 5-Ethoxysorgoleone | 0.112 | -0.120 | 0.602 | 0.731 | 0.151 | 0.667 |
| 18-hydroxy-epoxy-stearic acid | 0.175 | -0.188 | 0.608 | 0.717 | 0.048 | 0.966 |
| Sarmentosin epoxide | 0.055 | -0.059 | 0.609 | 0.716 | 0.558 | 0.552 |
| 4-Hydroxystyrene* | 0.229 | -0.246 | 0.612 | 0.709 | 0.048 | 1.081 |
| 2-C-Methyl-D-erythritol phosphate | -0.004 | 0.004 | 0.613 | 0.706 | 0.975 | 0.518 |
| 5-Hydroxypropafenone* | 0.212 | -0.228 | 0.613 | 0.705 | 0.040 | 1.324 |
| 8-CMD* | 0.368 | -0.395 | 0.617 | 0.696 | 0.023 | 1.856 |
| Ursodeoxycholic acid 3-sulfate | 0.212 | -0.228 | 0.620 | 0.690 | 0.100 | 1.065 |
| 2-(chloromethyl)-Pyrimidine | 0.203 | -0.218 | 0.620 | 0.689 | 0.010 | 0.929 |
| m-Chlorophenylbiguanide | 0.227 | -0.244 | 0.623 | 0.682 | 0.093 | 1.414 |
| Costunolide | 0.158 | -0.169 | 0.627 | 0.673 | 0.004 | 0.948 |
| Pioglitazone | -0.284 | 0.305 | 0.633 | 0.661 | 0.098 | 1.417 |
| GMSO* | 0.192 | -0.206 | 0.634 | 0.658 | 0.000 | 1.166 |
| 6-O-Methylnorlaudanosoline | 0.095 | -0.102 | 0.641 | 0.642 | 0.627 | 1.133 |
| N-Methylethanolamine phosphate* | 0.175 | -0.188 | 0.643 | 0.637 | 0.003 | 1.058 |
| Sodium Tetradecyl Sulfate | 0.152 | -0.163 | 0.643 | 0.637 | 0.010 | 0.775 |
| Succinic aldehyde | 0.124 | -0.133 | 0.645 | 0.632 | 0.125 | 0.823 |
| (+)-Ligballinol | 0.061 | -0.066 | 0.648 | 0.625 | 0.763 | 1.835 |
| Cuscohygrine* | 0.191 | -0.205 | 0.653 | 0.615 | 0.000 | 1.106 |
| Tiletamine | 0.103 | -0.110 | 0.654 | 0.613 | 0.227 | 0.745 |
| Tetrahydropersin | 0.060 | -0.064 | 0.654 | 0.613 | 0.480 | 0.346 |
| Dihydrourocanate | 0.086 | -0.093 | 0.655 | 0.611 | 0.586 | 0.480 |
| 20-Eicosanolide | 0.095 | -0.102 | 0.657 | 0.606 | 0.288 | 0.515 |
| Myr-3-TAX* | 0.364 | -0.391 | 0.659 | 0.601 | 0.011 | 1.634 |
| cis,cis-3,6-Dodecadienoyl-CoA* | 0.298 | -0.320 | 0.661 | 0.598 | 0.016 | 1.622 |
| (R)-Malate | 0.170 | -0.183 | 0.661 | 0.597 | 0.027 | 0.847 |
| Lys Gly His* | 0.305 | -0.327 | 0.662 | 0.596 | 0.017 | 1.758 |
| Octadecanoic acid trichloroethyl ester | 0.262 | -0.282 | 0.663 | 0.593 | 0.062 | 1.777 |
| His Gly Ser | -0.025 | 0.027 | 0.667 | 0.585 | 0.838 | 0.699 |
| Gln Pro Leu Leu | 0.079 | -0.085 | 0.669 | 0.580 | 0.250 | 0.699 |
| Orthoform | 0.485 | -0.521 | 0.671 | 0.577 | 0.003 | 2.786 |
| 10-Deoxymethynolide | 0.162 | -0.174 | 0.672 | 0.573 | 0.017 | 0.985 |
| N-Undecylbenzenesulfonic acid | 0.180 | -0.194 | 0.672 | 0.573 | 0.020 | 0.802 |
| 5-Hydroxyconiferyl alcohol | 0.048 | -0.051 | 0.673 | 0.571 | 0.778 | 0.799 |
| Anthraquinone | 0.447 | -0.480 | 0.677 | 0.562 | 0.003 | 2.642 |
| PE(P-16:0/0:0) | 0.083 | -0.089 | 0.678 | 0.562 | 0.358 | 0.595 |
| Methylglyoxal | 0.145 | -0.155 | 0.678 | 0.561 | 0.083 | 0.876 |
| cis-Acetylacrylate | 0.065 | -0.070 | 0.680 | 0.556 | 0.461 | 0.677 |
| PA(15:1(9Z)/0:0) | 0.006 | -0.006 | 0.684 | 0.549 | 0.960 | 0.571 |
| 2-Carboxyphenylamino-DR5P | 0.446 | -0.479 | 0.687 | 0.542 | 0.006 | 2.128 |
| 2-(1-Propenyl)-delta1-piperideine | 0.223 | -0.240 | 0.687 | 0.541 | 0.000 | 1.354 |
| 1-Isothiocyanatobutane | 0.009 | -0.009 | 0.688 | 0.539 | 0.960 | 0.701 |
| Lys Ile Cys | -0.037 | 0.040 | 0.693 | 0.529 | 0.745 | 0.474 |
| Rhizocticin A | 0.156 | -0.167 | 0.698 | 0.520 | 0.002 | 0.919 |
| Narcissidine | 0.154 | -0.165 | 0.699 | 0.516 | 0.004 | 0.939 |
| 8-Amino-7-oxononanoate | 0.075 | -0.080 | 0.699 | 0.516 | 0.246 | 0.491 |
| PE(18:2(9Z,12Z)/0:0) | -0.152 | 0.163 | 0.701 | 0.512 | 0.359 | 0.773 |
| L-Rhamnulose 1-phosphate | -0.054 | 0.058 | 0.704 | 0.506 | 0.769 | 1.213 |
| N-oleoyl tyrosine | 0.109 | -0.117 | 0.707 | 0.500 | 0.540 | 0.778 |
| 4',6,7-Trihydroxyisoflavone | -0.035 | 0.038 | 0.709 | 0.495 | 0.857 | 0.617 |
| Panamine | 0.256 | -0.275 | 0.710 | 0.494 | 0.104 | 1.380 |
| Trp Arg Phe Glu | 0.131 | -0.141 | 0.713 | 0.489 | 0.075 | 0.847 |
| 16-Hydroxypalmitate | 0.237 | -0.254 | 0.713 | 0.488 | 0.022 | 1.360 |
| Thr Asp Cys Cys | 0.160 | -0.172 | 0.715 | 0.485 | 0.001 | 0.979 |
| 2-Methylfuran | 0.190 | -0.204 | 0.716 | 0.481 | 0.059 | 1.263 |
| 6-Acetyl-D-glucose | 0.074 | -0.080 | 0.717 | 0.479 | 0.360 | 0.487 |
| Arg Leu Arg | 0.083 | -0.090 | 0.717 | 0.479 | 0.616 | 0.803 |
| Dihydroxy-isobutyric acid | 0.117 | -0.126 | 0.718 | 0.478 | 0.215 | 1.055 |
| 8-C-Glucosylnaringenin | -0.140 | 0.150 | 0.719 | 0.475 | 0.478 | 1.455 |
| Cyclopassifloic acid B | 0.299 | -0.321 | 0.720 | 0.475 | 0.076 | 1.592 |
| Glycolaldehyde | 0.091 | -0.098 | 0.720 | 0.474 | 0.273 | 0.670 |
| 9-Hydroxybenzo[a]pyrene-oxide | -0.094 | 0.101 | 0.721 | 0.472 | 0.588 | 0.912 |
| Microlenin | 0.148 | -0.159 | 0.721 | 0.471 | 0.045 | 0.792 |
| Boc-Pro-DVal(NMe)-Val-OMe | 0.049 | -0.052 | 0.723 | 0.469 | 0.782 | 0.951 |
| Glu Phe Trp | 0.142 | -0.152 | 0.728 | 0.459 | 0.402 | 0.842 |
| Trimethyl-2,6,10-dodecatrienyl heptanoate | 0.040 | -0.043 | 0.729 | 0.456 | 0.651 | 0.649 |
| Dehydroepiandrosterone sulfate | 0.252 | -0.271 | 0.729 | 0.456 | 0.114 | 1.354 |
| Petasitenine | 0.196 | -0.210 | 0.730 | 0.455 | 0.000 | 1.162 |
| 2(α-D-Mannosyl)-D-glycerate | 0.156 | -0.168 | 0.733 | 0.447 | 0.001 | 0.820 |
| Decyl isobutyrate | 0.120 | -0.129 | 0.734 | 0.446 | 0.094 | 0.827 |
| 2-keto palmitic acid | 0.173 | -0.186 | 0.734 | 0.445 | 0.000 | 1.044 |
| 13(S)-HODE | 0.103 | -0.111 | 0.734 | 0.445 | 0.221 | 0.691 |
| (R)-3,3-Dimethylmalate | 0.034 | -0.036 | 0.735 | 0.444 | 0.676 | 0.466 |
| Echitovenine | 0.104 | -0.112 | 0.736 | 0.443 | 0.107 | 0.708 |
| TG(19:1) | 0.161 | -0.173 | 0.737 | 0.440 | 0.007 | 0.942 |
| 6-Hydroxydexamethasone | 0.147 | -0.158 | 0.738 | 0.438 | 0.004 | 0.972 |
| Acetylenedicarboxylate | 0.099 | -0.107 | 0.739 | 0.436 | 0.224 | 0.865 |
| Acrolein | 0.061 | -0.065 | 0.742 | 0.431 | 0.470 | 0.666 |
| Losartan | 0.197 | -0.212 | 0.742 | 0.430 | 0.065 | 1.057 |
| Bretylium | 0.127 | -0.136 | 0.746 | 0.422 | 0.263 | 0.748 |
| Nilotinib | 0.276 | -0.296 | 0.751 | 0.413 | 0.049 | 1.255 |
| Myxalamid A | 0.182 | -0.195 | 0.751 | 0.412 | 0.338 | 1.332 |
| Traumatic acid | 0.174 | -0.187 | 0.752 | 0.411 | 0.001 | 1.199 |
| N-docosahexaenoyl glutamic acid | -0.007 | 0.008 | 0.753 | 0.409 | 0.967 | 0.913 |
| Prostaglandin D2-1-glyceryl ester | 0.066 | -0.071 | 0.755 | 0.405 | 0.238 | 0.433 |
| Cinnamyl benzoate | 0.170 | -0.183 | 0.756 | 0.404 | 0.106 | 1.181 |
| Heliotrine | 0.121 | -0.130 | 0.757 | 0.402 | 0.066 | 0.755 |
| Myristoleyl arachidonate | 0.130 | -0.140 | 0.759 | 0.398 | 0.226 | 1.131 |
| D-erythro-Sphingosine C-20 | 0.158 | -0.169 | 0.761 | 0.394 | 0.041 | 0.558 |
| PG(O-20:0/21:0) | 0.165 | -0.177 | 0.766 | 0.385 | 0.005 | 0.988 |
| Aspirin | -0.028 | 0.030 | 0.766 | 0.385 | 0.705 | 0.163 |
| 2-Hydroxy-IMPY | 0.087 | -0.093 | 0.767 | 0.382 | 0.227 | 0.533 |
| cis-Resveratrol 3-sulfate | 0.174 | -0.187 | 0.771 | 0.375 | 0.001 | 1.038 |
| Asp Asp Cys Phe | 0.153 | -0.164 | 0.773 | 0.372 | 0.003 | 0.990 |
| Dieporeticenin | -0.052 | 0.056 | 0.773 | 0.372 | 0.787 | 1.647 |
| PS(16:1/22:2) | 0.160 | -0.172 | 0.776 | 0.367 | 0.413 | 1.890 |
| TG(17:2/18:4/20:4) | 0.176 | -0.189 | 0.778 | 0.362 | 0.042 | 1.064 |
| 2-Hydroxymyristic Acid | 0.252 | -0.271 | 0.779 | 0.360 | 0.052 | 1.577 |
| S 1033 | 0.095 | -0.102 | 0.779 | 0.360 | 0.122 | 0.642 |
| Glu Trp Tyr | 0.105 | -0.113 | 0.781 | 0.357 | 0.115 | 0.647 |
| 2-oxo-octadecanoic acid | 0.058 | -0.062 | 0.781 | 0.357 | 0.428 | 0.408 |
| Norcapsaicin | -0.117 | 0.126 | 0.782 | 0.355 | 0.511 | 1.476 |
| 9,10-dihydroxyoctadecanoic acid | 0.040 | -0.043 | 0.783 | 0.353 | 0.717 | 1.116 |
| PR-toxin | 0.332 | -0.357 | 0.784 | 0.350 | 0.021 | 2.147 |
| Bismuth subgallate | 0.150 | -0.161 | 0.785 | 0.349 | 0.161 | 1.110 |
| His Met Phe | 0.152 | -0.163 | 0.786 | 0.348 | 0.179 | 0.849 |
| Asp His Gly | 0.071 | -0.077 | 0.787 | 0.346 | 0.604 | 0.466 |
| Bis(2-methylpropanoyloxy)-p-mentha | 0.159 | -0.171 | 0.787 | 0.345 | 0.001 | 0.976 |
| PG(13:0/0:0) | 0.018 | -0.020 | 0.788 | 0.344 | 0.874 | 0.681 |
| Threoninyl-Serine | 0.088 | -0.095 | 0.789 | 0.342 | 0.231 | 0.432 |
| Ala Arg Asn Asp | 0.107 | -0.115 | 0.790 | 0.340 | 0.086 | 0.709 |
| 1α,25-Dihydroxy-D3-Thia | 0.183 | -0.197 | 0.790 | 0.339 | 0.300 | 1.556 |
| N-Acetylmannosamine | 0.112 | -0.121 | 0.793 | 0.334 | 0.118 | 0.632 |
| Cer(t18:0/18:0) | 0.164 | -0.176 | 0.795 | 0.330 | 0.245 | 0.864 |
| TG(15:1/17:1/17:2) | 0.143 | -0.153 | 0.796 | 0.330 | 0.018 | 0.875 |
| Traumatin | 0.146 | -0.157 | 0.796 | 0.329 | 0.029 | 1.048 |
| PG(16:1(9Z)/0:0) | 0.130 | -0.140 | 0.797 | 0.327 | 0.234 | 0.822 |
| 1-Keto-D-chiro-inositol | 0.105 | -0.113 | 0.800 | 0.322 | 0.183 | 0.711 |
| Isorhamnetin-3-O-β-D-Triacetylglucoside | 0.110 | -0.118 | 0.801 | 0.319 | 0.023 | 0.821 |
| D-Glycerate | 0.060 | -0.065 | 0.802 | 0.319 | 0.436 | 0.364 |
| (2R)-2-Hydroxy-2-methylbutanenitrile | 0.173 | -0.186 | 0.802 | 0.319 | 0.190 | 1.035 |
| 22:0-Glc-Sitosterol | 0.198 | -0.212 | 0.802 | 0.319 | 0.202 | 1.508 |
| Gln Pro His Cys | 0.307 | -0.330 | 0.802 | 0.318 | 0.079 | 1.652 |
| 5-Hydroxythiabendazole | 0.138 | -0.149 | 0.804 | 0.315 | 0.004 | 0.946 |
| Homocapsaicin | 0.174 | -0.187 | 0.804 | 0.315 | 0.130 | 1.078 |
| N-cis-Tetradecenoyl-HSL | 0.065 | -0.070 | 0.804 | 0.315 | 0.308 | 0.626 |
| Harpagoside | -0.034 | 0.036 | 0.806 | 0.311 | 0.862 | 1.118 |
| 26-hydroxycholesterol 3-sulfate | 0.311 | -0.334 | 0.807 | 0.310 | 0.032 | 1.554 |
| PA(O-16:0/14:1(9Z)) | 0.161 | -0.173 | 0.807 | 0.309 | 0.039 | 0.959 |
| 5-Phenyl-1,3-oxazinane-2,4-dione | 0.053 | -0.057 | 0.809 | 0.307 | 0.705 | 0.547 |
| PG(20:3(8Z,11Z,14Z)/0:0) | 0.133 | -0.143 | 0.811 | 0.302 | 0.013 | 0.887 |
| Thiodiacetic acid | 0.042 | -0.046 | 0.812 | 0.300 | 0.801 | 0.684 |
| 17α,20α-Dihydroxycholesterol | 0.024 | -0.025 | 0.813 | 0.299 | 0.840 | 0.806 |
| 2-Phenyl-1,3-propanediol carbamate | -0.168 | 0.180 | 0.813 | 0.299 | 0.308 | 0.959 |
| PG(P-18:0/18:1(9Z)) | 0.099 | -0.106 | 0.814 | 0.297 | 0.618 | 1.799 |
| Aphidicolin | 0.126 | -0.135 | 0.817 | 0.292 | 0.021 | 0.771 |
| His Arg Tyr Asp | 0.073 | -0.079 | 0.817 | 0.291 | 0.193 | 0.487 |
| JWH 200 7-hydroxyindole metabolite | 0.165 | -0.178 | 0.818 | 0.289 | 0.121 | 0.890 |
| Q-7-Me-3-CG-Glu | 0.117 | -0.126 | 0.819 | 0.289 | 0.015 | 0.818 |
| MG(0:0/18:1(9Z)/0:0) | 0.077 | -0.083 | 0.822 | 0.283 | 0.440 | 0.847 |
| 3-Methylbutan-2-one | 0.109 | -0.117 | 0.823 | 0.282 | 0.285 | 0.627 |
| Carnosine | 0.198 | -0.213 | 0.823 | 0.281 | 0.104 | 0.898 |
| 7-Formyldehydrothalicsimidine | 0.336 | -0.361 | 0.824 | 0.280 | 0.020 | 2.062 |
| Germacrene A acid | 0.034 | -0.036 | 0.824 | 0.280 | 0.531 | 0.434 |
| 2-(Acetamidomethylene)succinate | 0.036 | -0.038 | 0.826 | 0.277 | 0.647 | 0.319 |
| Methylpyrazine | 0.148 | -0.159 | 0.826 | 0.275 | 0.188 | 0.693 |
| Mesoporphyrin IX | 0.086 | -0.092 | 0.827 | 0.273 | 0.619 | 0.563 |
| Ethiprole | 0.157 | -0.169 | 0.830 | 0.269 | 0.031 | 1.070 |
| Val Arg Tyr | 0.067 | -0.072 | 0.831 | 0.267 | 0.599 | 0.997 |
| His His Ser | 0.053 | -0.057 | 0.832 | 0.265 | 0.323 | 0.428 |
| Threoninyl-Glycine | -0.052 | 0.056 | 0.833 | 0.264 | 0.639 | 0.602 |
| Noviflumuron | 0.160 | -0.172 | 0.833 | 0.264 | 0.177 | 1.006 |
| Val Arg Leu Lys | 0.121 | -0.130 | 0.835 | 0.259 | 0.149 | 0.799 |
| Benzodioxol-propyl benzoate | 0.115 | -0.123 | 0.836 | 0.259 | 0.010 | 0.810 |
| D-Arginine | -0.125 | 0.135 | 0.836 | 0.259 | 0.380 | 1.077 |
| Bouillonamide A | -0.022 | 0.024 | 0.838 | 0.255 | 0.897 | 1.106 |
| Myxochelin B | 0.131 | -0.141 | 0.838 | 0.255 | 0.058 | 0.785 |
| cis-CE-Cyclohexadiene-diol | -0.002 | 0.002 | 0.838 | 0.254 | 0.992 | 0.979 |
| 4-Methylaminobutyrate | 0.220 | -0.236 | 0.841 | 0.250 | 0.081 | 1.113 |
| S-(4-Bromophenyl)-mercaptopyruvate | 0.125 | -0.134 | 0.842 | 0.249 | 0.012 | 0.981 |
| Oxidized Photinus luciferin | 0.150 | -0.161 | 0.842 | 0.248 | 0.064 | 1.070 |
| Cortolone | 0.025 | -0.027 | 0.843 | 0.246 | 0.788 | 0.837 |
| Lys Lys Trp | 0.033 | -0.035 | 0.844 | 0.245 | 0.662 | 0.494 |
| 3-Hydroxy-2-naphthoate | 0.032 | -0.035 | 0.844 | 0.244 | 0.827 | 0.702 |
| 22-Hydroxydocosanoate | -0.044 | 0.047 | 0.845 | 0.243 | 0.755 | 0.782 |
| MG(18:2(9Z,12Z)/0:0/0:0) | 0.089 | -0.096 | 0.847 | 0.240 | 0.396 | 0.916 |
| 4-Carboxy-4-Methyl-Cholesterol | 0.090 | -0.097 | 0.849 | 0.237 | 0.649 | 1.196 |
| Stigmastan-3,5-diene | 0.069 | -0.074 | 0.851 | 0.233 | 0.197 | 0.580 |
| Glycerone | 0.101 | -0.109 | 0.852 | 0.230 | 0.019 | 0.733 |
| Cardanolide skeleton | 0.100 | -0.107 | 0.853 | 0.230 | 0.486 | 1.090 |
| Feruloylputrescine | 0.081 | -0.087 | 0.854 | 0.228 | 0.439 | 0.739 |
| Hexadecan-3-one | 0.118 | -0.126 | 0.854 | 0.227 | 0.098 | 0.751 |
| Cucujolide IX | 0.048 | -0.051 | 0.854 | 0.227 | 0.360 | 0.420 |
| 6-Lactoyl-5,6,7,8-tetrahydropterin | -0.163 | 0.175 | 0.855 | 0.227 | 0.233 | 1.035 |
| Dopamine 4-sulfate | 0.189 | -0.203 | 0.857 | 0.223 | 0.241 | 1.235 |
| Flufenacet | 0.095 | -0.102 | 0.857 | 0.223 | 0.047 | 0.815 |
| Tropolone | -0.061 | 0.065 | 0.858 | 0.221 | 0.596 | 0.445 |
| TG(15:0/18:3/22:4) | 0.052 | -0.056 | 0.858 | 0.221 | 0.541 | 0.840 |
| Formyl-N-acetyl-5-methoxykynurenamine | 0.132 | -0.142 | 0.858 | 0.221 | 0.043 | 0.858 |
| 17α,21-Dihydroxypregnenolone | 0.117 | -0.125 | 0.860 | 0.218 | 0.072 | 0.743 |
| Leu Leu Lys Lys | 0.213 | -0.228 | 0.860 | 0.218 | 0.085 | 1.378 |
| 4,5-Dimethyl-4-hexen-3-one | 0.041 | -0.044 | 0.862 | 0.215 | 0.433 | 0.324 |
| Perfluorooctanoic acid | 0.088 | -0.095 | 0.862 | 0.215 | 0.030 | 0.542 |
| PS(18:2(9Z,12Z)/0:0) | 0.052 | -0.056 | 0.863 | 0.213 | 0.369 | 0.392 |
| Phe Arg Ala | 0.110 | -0.118 | 0.865 | 0.210 | 0.019 | 0.777 |
| PG(P-16:0/14:1(9Z)) | 0.123 | -0.132 | 0.865 | 0.209 | 0.296 | 0.816 |
| Typhasterol | 0.165 | -0.177 | 0.866 | 0.208 | 0.150 | 0.752 |
| Arg Pro Lys Phe | 0.130 | -0.139 | 0.866 | 0.208 | 0.265 | 0.673 |
| Montanol | -0.146 | 0.157 | 0.867 | 0.205 | 0.308 | 1.133 |
| Panaquinquecol 7 | 0.085 | -0.092 | 0.868 | 0.204 | 0.221 | 0.481 |
| Isopropyl apiosylglucoside | -0.019 | 0.021 | 0.869 | 0.203 | 0.919 | 1.815 |
| Lucidenic acid C | 0.096 | -0.103 | 0.869 | 0.202 | 0.597 | 1.477 |
| Teflubenzuron | 0.310 | -0.333 | 0.871 | 0.200 | 0.029 | 1.841 |
| cis-Jasmone | 0.069 | -0.074 | 0.871 | 0.199 | 0.215 | 0.606 |
| C16 Sulfatide | 0.029 | -0.031 | 0.871 | 0.199 | 0.878 | 1.244 |
| L-Serine | 0.140 | -0.151 | 0.872 | 0.198 | 0.182 | 0.813 |
| Decylubiquinol | 0.093 | -0.100 | 0.872 | 0.197 | 0.067 | 0.657 |
| Decyl isobutyrate | 0.128 | -0.137 | 0.873 | 0.195 | 0.095 | 0.793 |
| Dihydroxy-hexahydroindolizin | 0.084 | -0.091 | 0.874 | 0.194 | 0.171 | 0.637 |
| 5α-Dihydrodeoxycorticosterone | 0.073 | -0.079 | 0.876 | 0.191 | 0.211 | 0.421 |
| Dihydro-7-desacetyldeoxygedunin | 0.147 | -0.158 | 0.878 | 0.187 | 0.152 | 1.159 |
| TG(15:0/15:1/18:4) | 0.334 | -0.359 | 0.879 | 0.185 | 0.034 | 1.552 |
| 6'-O-Galloylsucrose | 0.089 | -0.095 | 0.880 | 0.185 | 0.165 | 0.779 |
| Erythrinasinate A | 0.039 | -0.042 | 0.880 | 0.184 | 0.633 | 0.791 |
| (Z)-7-Dodecenyl propionate | 0.115 | -0.124 | 0.881 | 0.182 | 0.396 | 0.641 |
| Phe Arg Val | 0.162 | -0.174 | 0.883 | 0.180 | 0.039 | 1.090 |
| Methyl jasmonate | 0.049 | -0.053 | 0.883 | 0.179 | 0.352 | 0.347 |
| Taxa-4(20),11(12)-dien-5α-yl acetate | 0.103 | -0.110 | 0.883 | 0.179 | 0.324 | 0.610 |
| Coniferyl acetate | 0.073 | -0.079 | 0.884 | 0.178 | 0.185 | 0.582 |
| TG(19:1/20:1/20:2) | 0.057 | -0.062 | 0.887 | 0.174 | 0.433 | 0.698 |
| Cohibin D | -0.023 | 0.025 | 0.887 | 0.173 | 0.855 | 1.078 |
| Enalkiren | 0.080 | -0.086 | 0.887 | 0.173 | 0.622 | 0.747 |
| Taxa acetate | 0.091 | -0.098 | 0.888 | 0.171 | 0.251 | 0.734 |
| Malformin | 0.157 | -0.168 | 0.888 | 0.171 | 0.192 | 0.908 |
| Allopregnanalone sulfate | 0.262 | -0.282 | 0.889 | 0.170 | 0.071 | 1.175 |
| U 0521 | -0.228 | 0.245 | 0.890 | 0.168 | 0.137 | 1.454 |
| Pyrrolidine | 0.044 | -0.047 | 0.890 | 0.167 | 0.371 | 0.385 |
| Methyl-Fucogalactoside | 0.183 | -0.197 | 0.892 | 0.166 | 0.330 | 1.685 |
| 7-Methyloctyl 5-methylhexanoate | 0.072 | -0.077 | 0.893 | 0.164 | 0.230 | 0.590 |
| Bisdemethoxycurcumin | -0.123 | 0.132 | 0.894 | 0.161 | 0.533 | 1.389 |
| PG(20:2/22:4) | -0.005 | 0.005 | 0.894 | 0.161 | 0.978 | 1.659 |
| PC(9:0/0:0) | 0.071 | -0.076 | 0.895 | 0.160 | 0.147 | 0.655 |
| 3-Dimethylallyl-4-Hydroxy-MA | 0.046 | -0.049 | 0.897 | 0.157 | 0.354 | 0.447 |
| Xanthine | 0.170 | -0.183 | 0.899 | 0.154 | 0.149 | 0.991 |
| 4,10-undecadiynal | -0.001 | 0.001 | 0.901 | 0.150 | 0.986 | 0.153 |
| N-Succinyl-L-glutamate | 0.074 | -0.079 | 0.902 | 0.148 | 0.105 | 0.663 |
| β-Phenylalanoyl-CoA | 0.075 | -0.080 | 0.903 | 0.147 | 0.131 | 0.634 |
| Nonadecylic acid | 0.062 | -0.067 | 0.905 | 0.144 | 0.310 | 0.364 |
| Thyrotropin-releasing hormone | -0.008 | 0.009 | 0.905 | 0.144 | 0.889 | 0.313 |
| Tetrahydrocorticosterone | 0.041 | -0.044 | 0.906 | 0.143 | 0.502 | 0.633 |
| Icosadienoic acid | 0.090 | -0.097 | 0.906 | 0.143 | 0.210 | 0.421 |
| 9,10-Dihydroxy-12,13-epoxy-ODA | -0.099 | 0.106 | 0.906 | 0.142 | 0.458 | 0.877 |
| Nicotinate D-ribonucleotide | 0.078 | -0.084 | 0.907 | 0.141 | 0.146 | 0.537 |
| Templetine | 0.168 | -0.180 | 0.908 | 0.139 | 0.133 | 0.905 |
| Thiamine acetic acid | 0.017 | -0.018 | 0.908 | 0.139 | 0.823 | 0.376 |
| 6-Hydroxyethyl-dihydrosanguinarine | -0.138 | 0.149 | 0.911 | 0.135 | 0.293 | 1.076 |
| 1,7-Dimethyluric acid | 0.006 | -0.006 | 0.911 | 0.135 | 0.976 | 0.484 |
| 3-Hydroxy-apo-carotenal | 0.045 | -0.048 | 0.911 | 0.134 | 0.667 | 0.807 |
| PI(16:0/0:0) | 0.183 | -0.197 | 0.912 | 0.134 | 0.233 | 1.022 |
| DM-5-Methyl-7-Propyl-Indolizine | 0.090 | -0.096 | 0.912 | 0.133 | 0.208 | 0.722 |
| Glu Glu Phe Met | 0.093 | -0.100 | 0.912 | 0.133 | 0.221 | 0.794 |
| PE(18:1(9Z)/0:0) | 0.055 | -0.059 | 0.912 | 0.133 | 0.412 | 0.355 |
| 4-Methylhexan-2-one | 0.097 | -0.104 | 0.913 | 0.132 | 0.123 | 0.771 |
| Thr Arg Arg Ala | 0.043 | -0.046 | 0.913 | 0.131 | 0.527 | 0.388 |
| Sphingofungin E | 0.067 | -0.072 | 0.916 | 0.127 | 0.723 | 0.554 |
| WIN I(S) | -0.215 | 0.231 | 0.916 | 0.126 | 0.179 | 1.726 |
| Thonningianin B | 0.059 | -0.063 | 0.916 | 0.126 | 0.210 | 0.567 |
| Pentosidine | 0.081 | -0.087 | 0.917 | 0.125 | 0.266 | 0.661 |
| 2-Propylglutaric acid | 0.239 | -0.257 | 0.918 | 0.124 | 0.068 | 1.349 |
| Furmecyclox | 0.067 | -0.072 | 0.918 | 0.123 | 0.242 | 0.580 |
| Indolepyruvate | 0.027 | -0.029 | 0.918 | 0.123 | 0.712 | 0.245 |
| Thioridazine 2,5-disulfone | 0.076 | -0.082 | 0.919 | 0.123 | 0.113 | 0.681 |
| (S)-2-Hydroxypropylphosphonate | 0.037 | -0.039 | 0.919 | 0.122 | 0.835 | 0.768 |
| Lys Thr Gln | 0.135 | -0.146 | 0.919 | 0.121 | 0.209 | 0.830 |
| Phe Trp Trp | 0.052 | -0.056 | 0.920 | 0.121 | 0.355 | 0.466 |
| His Lys Met | 0.178 | -0.191 | 0.921 | 0.119 | 0.307 | 1.249 |
| Naltrindole | 0.084 | -0.091 | 0.922 | 0.117 | 0.102 | 0.706 |
| Asn Arg Thr Phe | -0.035 | 0.037 | 0.922 | 0.117 | 0.833 | 1.361 |
| Dihydroxy-DMOI | 0.061 | -0.066 | 0.924 | 0.114 | 0.414 | 0.540 |
| (S)-Methylmalonate semialdehyde | -0.050 | 0.054 | 0.925 | 0.113 | 0.503 | 0.222 |
| Pyridoindolyl-butanediol | 0.082 | -0.088 | 0.925 | 0.112 | 0.546 | 0.443 |
| Apigenin 7-GlcA-Malonyl-Glc | 0.130 | -0.140 | 0.926 | 0.111 | 0.504 | 1.266 |
| Nelumboside | 0.042 | -0.046 | 0.926 | 0.111 | 0.412 | 0.588 |
| Dihydrodeoxygedunin | 0.343 | -0.368 | 0.926 | 0.110 | 0.026 | 1.902 |
| 7-Methyloctyl octanoate | 0.045 | -0.048 | 0.928 | 0.108 | 0.576 | 0.766 |
| Dihydroxy-Taxa-Acetate | 0.071 | -0.076 | 0.928 | 0.108 | 0.261 | 0.694 |
| 4Z-Nonen-2-one | 0.057 | -0.062 | 0.929 | 0.106 | 0.211 | 0.593 |
| 5-Ethylundecan-6-one | 0.259 | -0.278 | 0.929 | 0.106 | 0.095 | 1.775 |
| Teniposide | -0.066 | 0.071 | 0.930 | 0.105 | 0.549 | 0.286 |
| Osmanthuside A | 0.072 | -0.077 | 0.930 | 0.105 | 0.132 | 0.594 |
| Palmitoyl 3-carbacyclic PA | 0.072 | -0.077 | 0.932 | 0.102 | 0.506 | 0.868 |
| N-Nitrosopyrrolidine | 0.281 | -0.302 | 0.932 | 0.101 | 0.041 | 1.593 |
| 5-Pyridoxolactone | 0.134 | -0.144 | 0.933 | 0.100 | 0.208 | 1.044 |
| Glucopyranosylmoranoline | 0.178 | -0.191 | 0.935 | 0.097 | 0.382 | 1.784 |
| 2-oxo-octadecanoic acid | 0.000 | 0.000 | 0.935 | 0.096 | 0.997 | 0.949 |
| Lysyl-Valine | 0.129 | -0.138 | 0.937 | 0.094 | 0.239 | 1.043 |
| N-Formylmethionine | 0.031 | -0.033 | 0.937 | 0.094 | 0.851 | 0.805 |
| Panaxacol | 0.051 | -0.054 | 0.937 | 0.094 | 0.465 | 0.322 |
| AM2232 | 0.034 | -0.037 | 0.937 | 0.094 | 0.497 | 0.335 |
| 5Z,12Z-otadecadienoic acid | 0.053 | -0.057 | 0.938 | 0.092 | 0.428 | 0.611 |
| α-Phocaecholic acid | 0.071 | -0.076 | 0.940 | 0.090 | 0.142 | 0.602 |
| C.I. Food Brown 3 | 0.038 | -0.041 | 0.940 | 0.089 | 0.787 | 0.226 |
| Pro Lys Gln | 0.131 | -0.141 | 0.941 | 0.087 | 0.254 | 0.992 |
| DG(16:0e/18:0/0:0) | 0.112 | -0.121 | 0.941 | 0.087 | 0.315 | 0.883 |
| Salvianolic acid L | -0.059 | 0.063 | 0.943 | 0.085 | 0.653 | 0.809 |
| 6-(Isopropylthio)purine | 0.024 | -0.026 | 0.944 | 0.082 | 0.689 | 0.292 |
| JWH213 | 0.006 | -0.007 | 0.945 | 0.082 | 0.966 | 1.025 |
| PE(22:6/20:3) | 0.159 | -0.171 | 0.946 | 0.080 | 0.251 | 1.130 |
| [6]-Gingerol | -0.035 | 0.037 | 0.947 | 0.079 | 0.535 | 0.220 |
| CAY10589 | 0.046 | -0.050 | 0.947 | 0.079 | 0.563 | 0.367 |
| Aminoparathion | -0.122 | 0.131 | 0.949 | 0.076 | 0.298 | 0.630 |
| 3β,5β-Ketotriol | 0.151 | -0.162 | 0.949 | 0.076 | 0.298 | 1.149 |
| 2,4-Dichloro-3-oxoadipate | 0.045 | -0.049 | 0.953 | 0.070 | 0.781 | 0.679 |
| Pangamic acid | 0.312 | -0.335 | 0.953 | 0.070 | 0.106 | 1.833 |
| Met His Met | -0.039 | 0.042 | 0.953 | 0.070 | 0.844 | 1.152 |
| Propanal | 0.078 | -0.084 | 0.953 | 0.069 | 0.226 | 0.677 |
| Treosulfan | 0.057 | -0.061 | 0.953 | 0.069 | 0.243 | 0.512 |
| Anethole | 0.029 | -0.031 | 0.954 | 0.068 | 0.575 | 0.285 |
| 2,4-Dichlorotoluene | -0.046 | 0.050 | 0.955 | 0.066 | 0.710 | 0.614 |
| cis,cis-dodeca-3,6-dienoic acid | -0.010 | 0.011 | 0.956 | 0.064 | 0.834 | 0.275 |
| Kanzonol W | 0.125 | -0.134 | 0.957 | 0.064 | 0.211 | 0.790 |
| (-)-Medicocarpin | 0.055 | -0.059 | 0.957 | 0.064 | 0.250 | 0.513 |
| PC(O-12:0/2:0) | -0.031 | 0.034 | 0.957 | 0.063 | 0.687 | 0.378 |
| 3-Methyl-2-butenal | -0.184 | 0.197 | 0.958 | 0.062 | 0.243 | 1.056 |
| Isoleucyl-Lysine | 0.030 | -0.032 | 0.958 | 0.062 | 0.616 | 0.257 |
| Ethylcyclohexane | 0.041 | -0.044 | 0.959 | 0.061 | 0.418 | 0.470 |
| Sudan Red 7B | 0.025 | -0.027 | 0.959 | 0.061 | 0.646 | 0.353 |
| 2-Deoxy-scyllo-inosamine | 0.092 | -0.099 | 0.959 | 0.060 | 0.377 | 0.784 |
| N2-Acetyl-L-AAP | -0.192 | 0.206 | 0.960 | 0.059 | 0.149 | 1.278 |
| Ginsenoside Rh4 | 0.157 | -0.168 | 0.960 | 0.059 | 0.218 | 0.793 |
| Cassiaside | 0.053 | -0.057 | 0.963 | 0.055 | 0.259 | 0.495 |
| 3β-Hydroxypregn sulfate | 0.309 | -0.331 | 0.965 | 0.052 | 0.091 | 1.756 |
| 4-Methylhistamine | 0.063 | -0.068 | 0.965 | 0.052 | 0.211 | 0.574 |
| Decyl acetate | 0.116 | -0.125 | 0.965 | 0.051 | 0.246 | 0.850 |
| 5-Acetamidopentanoate | -0.211 | 0.227 | 0.966 | 0.050 | 0.101 | 1.383 |
| Lys Gln Glu | 0.128 | -0.137 | 0.967 | 0.049 | 0.248 | 0.980 |
| Anhydrochlortetracycline | 0.025 | -0.026 | 0.969 | 0.046 | 0.606 | 0.313 |
| S-Japonin | 0.069 | -0.074 | 0.971 | 0.042 | 0.583 | 1.176 |
| 18:1(14Z) | 0.007 | -0.008 | 0.972 | 0.041 | 0.915 | 0.639 |
| 4-Methylaminobutyrate | 0.052 | -0.056 | 0.972 | 0.041 | 0.324 | 0.518 |
| all-trans-Retinoyl-β-glucuronide | -0.052 | 0.055 | 0.973 | 0.040 | 0.631 | 0.821 |
| BSA-arachidonoyl amine | 0.028 | -0.030 | 0.974 | 0.038 | 0.586 | 0.285 |
| Terfenadine | 0.167 | -0.180 | 0.975 | 0.037 | 0.342 | 0.661 |
| Anhalamine | 0.013 | -0.014 | 0.975 | 0.036 | 0.796 | 0.270 |
| 1-butyl-2-ethylcyclopentane | 0.045 | -0.048 | 0.975 | 0.036 | 0.411 | 0.433 |
| C.I. 14700 | 0.039 | -0.042 | 0.975 | 0.036 | 0.399 | 0.304 |
| 25R-Cholestane-Tetrol | 0.096 | -0.103 | 0.976 | 0.036 | 0.402 | 0.750 |
| C16-OH Sulfatide | -0.140 | 0.150 | 0.976 | 0.035 | 0.341 | 1.204 |
| Theasapogenol A | 0.409 | -0.439 | 0.977 | 0.034 | 0.009 | 2.361 |
| Orange B | 0.019 | -0.020 | 0.977 | 0.034 | 0.640 | 0.345 |
| Hexanethioic acid S-propyl ester | 0.050 | -0.054 | 0.978 | 0.032 | 0.312 | 0.467 |
| 5-Pentadecene | 0.036 | -0.039 | 0.979 | 0.031 | 0.498 | 0.421 |
| Sitosteryl glucoside | 0.112 | -0.120 | 0.980 | 0.030 | 0.271 | 0.664 |
| Methyleugenol | 0.017 | -0.018 | 0.984 | 0.023 | 0.742 | 0.403 |
| T2 Triol | 0.076 | -0.082 | 0.984 | 0.023 | 0.300 | 0.594 |
| 7α-Hydroxy-DHEA | 0.098 | -0.105 | 0.984 | 0.023 | 0.343 | 0.809 |
| Histamine | 0.050 | -0.053 | 0.986 | 0.020 | 0.334 | 0.393 |
| Propyl 1-(propylsulfinyl)propyl disulfide | 0.025 | -0.027 | 0.987 | 0.019 | 0.648 | 0.368 |
| C75 | 0.003 | -0.003 | 0.987 | 0.019 | 0.956 | 0.146 |
| Myrtine | 0.019 | -0.020 | 0.987 | 0.019 | 0.661 | 0.305 |
| PS(17:2(9Z,12Z)/0:0) | -0.013 | 0.014 | 0.989 | 0.017 | 0.916 | 0.581 |
| Isolithocholate | 0.010 | -0.010 | 0.989 | 0.016 | 0.862 | 0.286 |
| L-isoleucyl-L-proline | 0.157 | -0.169 | 0.990 | 0.014 | 0.145 | 0.784 |
| 2,4,6-Triaminotoluene | 0.039 | -0.042 | 0.991 | 0.014 | 0.404 | 0.427 |
| Resorcinol | -0.139 | 0.149 | 0.991 | 0.012 | 0.341 | 1.491 |
| 5-Hepten-2-one | 0.022 | -0.024 | 0.992 | 0.012 | 0.601 | 0.442 |
| ethyl 4-methyl-heptanoate | 0.111 | -0.119 | 0.993 | 0.010 | 0.305 | 0.917 |
| 10-Deacetylbaccatin III | -0.054 | 0.058 | 0.994 | 0.009 | 0.615 | 0.778 |
| Aclacinomycin T | 0.002 | -0.002 | 0.994 | 0.008 | 0.988 | 0.776 |
| Zolazepam | -0.024 | 0.026 | 0.994 | 0.008 | 0.651 | 0.273 |
| Trp Gln Pro Pro | -0.101 | 0.109 | 0.997 | 0.004 | 0.346 | 1.098 |
| Hypoxanthine | 0.000 | 0.000 | 0.998 | 0.003 | 0.994 | 0.502 |
| Gln Arg Arg Lys | 0.136 | -0.146 | 0.999 | 0.001 | 0.387 | 0.963 |
| PA(16:0/0:0) | 0.010 | -0.011 | 0.999 | 0.001 | 0.859 | 0.258 |
| 1-Hexadecen-3-one | 0.020 | -0.022 | 1.000 | 0.000 | 0.665 | 0.357 |
| Pseudouridine | -0.067 | 0.071 | 1.000 | 0.000 | 0.641 | 0.440 |
| PC(O-11:1(10E)/2:0) | 0.066 | -0.070 | 1.000 | 0.000 | 0.697 | 0.705 |
| Coniine | 0.021 | -0.023 | 1.001 | 0.001 | 0.636 | 0.367 |
| Isoamyl p-anisate | -0.024 | 0.026 | 1.003 | 0.004 | 0.664 | 0.130 |
| Diflunisal | 0.100 | -0.107 | 1.008 | 0.011 | 0.312 | 0.729 |
| Steryl sulfate | 0.169 | -0.181 | 1.008 | 0.011 | 0.177 | 1.024 |
| Allodeoxycholate | -0.039 | 0.042 | 1.008 | 0.011 | 0.795 | 1.224 |
| 7E-DHP | 0.026 | -0.028 | 1.009 | 0.013 | 0.590 | 0.338 |
| Leucodelphinidin | 0.261 | -0.280 | 1.010 | 0.014 | 0.052 | 1.569 |
| Tridecylic acid | 0.048 | -0.052 | 1.011 | 0.016 | 0.498 | 0.703 |
| Dodecan-3-one | 0.032 | -0.034 | 1.012 | 0.017 | 0.548 | 0.413 |
| Met-HoPhe-OH | 0.019 | -0.020 | 1.012 | 0.017 | 0.688 | 0.345 |
| TDE | 0.030 | -0.033 | 1.012 | 0.017 | 0.559 | 0.376 |
| Ala Pro Lys | 0.011 | -0.012 | 1.012 | 0.017 | 0.802 | 0.346 |
| Rutagravine | 0.107 | -0.115 | 1.012 | 0.018 | 0.320 | 0.888 |
| 2R-AHA | -0.013 | 0.014 | 1.013 | 0.018 | 0.770 | 0.228 |
| Hexadecanedioate | 0.113 | -0.122 | 1.013 | 0.018 | 0.074 | 0.633 |
| 5β-Cholestane-pentol | 0.066 | -0.071 | 1.013 | 0.019 | 0.696 | 1.411 |
| 8-Ethyl-Z-Hexyl-Methyl-Indolizine | 0.012 | -0.013 | 1.014 | 0.020 | 0.807 | 0.205 |
| (3S)-all-trans-3-Hydroxyretinal | 0.011 | -0.011 | 1.017 | 0.025 | 0.845 | 0.322 |
| Euglobal Ib | 0.027 | -0.029 | 1.019 | 0.026 | 0.639 | 0.324 |
| 7Z,11Z,13E-Hexadecatrienal | 0.101 | -0.108 | 1.019 | 0.028 | 0.302 | 0.516 |
| 17-Phenoxy-Trinor-PGF2α | 0.033 | -0.035 | 1.020 | 0.028 | 0.852 | 1.358 |
| Netilmicin | 0.025 | -0.027 | 1.021 | 0.030 | 0.673 | 0.294 |
| Fortimicin FU-10 | -0.126 | 0.135 | 1.021 | 0.030 | 0.535 | 1.478 |
| 3E,5E-tridecadienoic acid | -0.005 | 0.006 | 1.022 | 0.031 | 0.923 | 0.121 |
| 2,6-Dimethylaniline | -0.007 | 0.008 | 1.022 | 0.032 | 0.904 | 0.315 |
| 9(S)-HPOT | 0.051 | -0.054 | 1.023 | 0.032 | 0.498 | 0.647 |
| HDOPA | 0.006 | -0.006 | 1.023 | 0.033 | 0.907 | 0.248 |
| 3-Ethylheptadecan-2-one | -0.036 | 0.038 | 1.024 | 0.034 | 0.607 | 0.594 |
| 3-Butenenitrile | 0.024 | -0.026 | 1.024 | 0.034 | 0.622 | 0.357 |
| 2-Nonenyl acetate | 0.094 | -0.101 | 1.026 | 0.037 | 0.361 | 0.782 |
| N2-Acetyl-L-aminoadipate | -0.013 | 0.014 | 1.027 | 0.038 | 0.908 | 0.502 |
| 2-Oxo-4-phosphonobutanoate | 0.079 | -0.085 | 1.027 | 0.038 | 0.255 | 0.640 |
| Maritimetin | 0.007 | -0.007 | 1.029 | 0.041 | 0.900 | 0.341 |
| Quercetin 7-methyl ether disulfate | 0.035 | -0.038 | 1.029 | 0.042 | 0.572 | 0.395 |
| Nonoxynol-9 | 0.152 | -0.163 | 1.030 | 0.042 | 0.321 | 1.019 |
| 8,8-Dimethoxy-2-octanol | 0.005 | -0.005 | 1.030 | 0.043 | 0.917 | 0.277 |
| 9S,10R-dihydroxy-stearic acid | 0.058 | -0.062 | 1.031 | 0.044 | 0.525 | 0.515 |
| Decadienoic isobutylamide | 0.004 | -0.005 | 1.033 | 0.046 | 0.933 | 0.123 |
| Capsiate | -0.003 | 0.003 | 1.035 | 0.050 | 0.983 | 1.116 |
| 11-chloro-12-hydroxy-octadecanoic acid | -0.017 | 0.018 | 1.036 | 0.052 | 0.864 | 0.766 |
| Glaucarubol glucoside | 0.003 | -0.003 | 1.040 | 0.057 | 0.948 | 0.217 |
| MG(0:0/16:0/0:0) | -0.014 | 0.015 | 1.041 | 0.057 | 0.827 | 0.129 |
| Lupinine | -0.009 | 0.010 | 1.041 | 0.058 | 0.830 | 0.060 |
| Selegiline | -0.007 | 0.008 | 1.042 | 0.059 | 0.872 | 0.124 |
| 22α-Hydroxy-5α-campestan | 0.048 | -0.052 | 1.042 | 0.059 | 0.722 | 1.059 |
| Tetradecyl isobutyrate | 0.087 | -0.093 | 1.042 | 0.059 | 0.289 | 0.548 |
| Val Met Leu Leu | -0.006 | 0.006 | 1.042 | 0.060 | 0.933 | 0.200 |
| Ala Phe Phe Gln | -0.030 | 0.032 | 1.042 | 0.059 | 0.589 | 0.278 |
| Herbacetin 7-quinoylglucoside | 0.002 | -0.002 | 1.043 | 0.060 | 0.964 | 0.441 |
| 2'-Hydroxygenistein | 0.080 | -0.086 | 1.043 | 0.060 | 0.649 | 1.232 |
| Amino-oxocyclohex-CoA | -0.094 | 0.101 | 1.043 | 0.061 | 0.371 | 0.603 |
| Tetramethyl-Cyclopentaquinolizine | 0.103 | -0.111 | 1.044 | 0.062 | 0.333 | 0.557 |
| 5-campestenone | 0.021 | -0.023 | 1.044 | 0.062 | 0.867 | 0.509 |
| 2S-amino-tridecanoic acid | -0.005 | 0.005 | 1.044 | 0.063 | 0.928 | 0.192 |
| 2-Dodecylbenzenesulfonic acid | 0.020 | -0.021 | 1.044 | 0.063 | 0.727 | 0.282 |
| Eremopetasinorol | 0.118 | -0.127 | 1.045 | 0.064 | 0.274 | 0.636 |
| Vinyl-L-NIO | 0.072 | -0.077 | 1.046 | 0.065 | 0.623 | 0.799 |
| 7-Methyloctyl octanoate | 0.025 | -0.027 | 1.048 | 0.067 | 0.737 | 0.314 |
| Arcaine | 0.100 | -0.108 | 1.048 | 0.067 | 0.338 | 0.860 |
| Strigolactone ABC-rings | 0.180 | -0.193 | 1.048 | 0.068 | 0.230 | 1.343 |
| 5-Hexyltetrahydro-furancarboxylic acid | 0.101 | -0.109 | 1.048 | 0.068 | 0.376 | 0.882 |
| Kaempferol 3-malonylneohesperidoside | 0.119 | -0.128 | 1.051 | 0.072 | 0.520 | 1.619 |
| Lucidine B | 0.186 | -0.200 | 1.051 | 0.072 | 0.168 | 1.372 |
| 8-Methyl-5-propyloctahydroindolizin-8-ol | -0.032 | 0.034 | 1.052 | 0.072 | 0.555 | 0.146 |
| 6,8-Dimethyl-5-hexahydroindolizine | -0.013 | 0.014 | 1.053 | 0.075 | 0.816 | 0.171 |
| 6-Hydroxynicotinate | -0.020 | 0.021 | 1.053 | 0.075 | 0.663 | 0.085 |
| Ile Asn Asn | 0.013 | -0.014 | 1.054 | 0.076 | 0.842 | 0.211 |
| 4,5-Dioxopentanoate | -0.047 | 0.051 | 1.055 | 0.077 | 0.476 | 0.213 |
| Sedoheptulose | -0.001 | 0.001 | 1.055 | 0.077 | 0.987 | 0.229 |
| 2-Oxo-8-methylthiooctanoic acid | 0.018 | -0.019 | 1.055 | 0.078 | 0.755 | 0.370 |
| 1-hydroxy-24-methylsulfonyl-25,26,27-trinorvitamin D3 | 0.205 | -0.220 | 1.056 | 0.078 | 0.118 | 0.968 |
| Tris(butoxyethyl)phosphate | -0.006 | 0.007 | 1.057 | 0.080 | 0.965 | 0.842 |
| Ala Thr His | 0.085 | -0.092 | 1.058 | 0.081 | 0.533 | 0.894 |
| Sulfociprofloxacin | -0.009 | 0.010 | 1.058 | 0.081 | 0.875 | 0.070 |
| 3-Butylpyridine | -0.024 | 0.026 | 1.058 | 0.082 | 0.595 | 0.111 |
| Kamahine C | -0.028 | 0.030 | 1.060 | 0.084 | 0.812 | 0.804 |
| Cyclohexane | -0.013 | 0.014 | 1.061 | 0.085 | 0.845 | 0.192 |
| Pisatoside | -0.039 | 0.041 | 1.062 | 0.087 | 0.613 | 0.498 |
| β-hydroxylauric acid | 0.044 | -0.048 | 1.064 | 0.090 | 0.562 | 0.512 |
| Threoninyl-Leucine | -0.022 | 0.024 | 1.065 | 0.090 | 0.773 | 0.487 |
| 2-amino-tetradecanoic acid | 0.073 | -0.078 | 1.065 | 0.091 | 0.565 | 0.298 |
| PG(15:1(9Z)/0:0) | 0.092 | -0.099 | 1.066 | 0.092 | 0.507 | 0.673 |
| N-Isobutyl-2,4,8-decatrienamide | -0.010 | 0.011 | 1.066 | 0.092 | 0.833 | 0.191 |
| 5a-Dihydrotestosterone sulfate | 0.199 | -0.214 | 1.067 | 0.093 | 0.219 | 1.145 |
| Butyl-Hydroxy-PE-PP | 0.002 | -0.002 | 1.068 | 0.096 | 0.970 | 0.207 |
| Methenamine | -0.009 | 0.010 | 1.069 | 0.096 | 0.846 | 0.182 |
| (R)-2,3-Dihydroxypropane-1-sulfonate | -0.006 | 0.006 | 1.069 | 0.097 | 0.910 | 0.175 |
| PG(18:0/22:6) | 0.071 | -0.076 | 1.071 | 0.098 | 0.708 | 1.759 |
| PI(22:0/20:0) | -0.026 | 0.028 | 1.073 | 0.102 | 0.696 | 0.271 |
| 7-Ethyl-3,11-dimethyl-tridecatetraene | -0.009 | 0.009 | 1.074 | 0.103 | 0.860 | 0.180 |
| PE(16:0/0:0) | -0.040 | 0.043 | 1.075 | 0.104 | 0.548 | 0.366 |
| Anthranilyl-CoA | -0.014 | 0.015 | 1.078 | 0.108 | 0.780 | 0.141 |
| Actinorhodin | -0.032 | 0.034 | 1.080 | 0.111 | 0.522 | 0.128 |
| Kammogenin | 0.025 | -0.027 | 1.081 | 0.112 | 0.863 | 1.274 |
| Pentahydroxy-DMF-7-Glu | -0.027 | 0.029 | 1.081 | 0.113 | 0.577 | 0.114 |
| Retinol Acetate | 0.052 | -0.056 | 1.082 | 0.113 | 0.575 | 0.686 |
| N'-Nitrosoanabasine | -0.073 | 0.078 | 1.082 | 0.114 | 0.183 | 0.308 |
| 7-Sulfocholic acid | 0.031 | -0.033 | 1.082 | 0.114 | 0.785 | 0.595 |
| L-3,4-Dihydroxybutan-2-one 4-phosphate | -0.045 | 0.049 | 1.083 | 0.115 | 0.476 | 0.282 |
| 4-Sulfobenzyl alcohol | -0.192 | 0.206 | 1.083 | 0.115 | 0.341 | 1.703 |
| PA(P-18:0/0:0) | 0.060 | -0.064 | 1.086 | 0.119 | 0.682 | 1.274 |
| Phe Pro Met Cys | -0.020 | 0.022 | 1.086 | 0.119 | 0.795 | 0.461 |
| 2-Oxo-5-methylthiopentanoic acid | 0.001 | -0.001 | 1.087 | 0.120 | 0.989 | 0.228 |
| Sinapoylputrescine | 0.055 | -0.059 | 1.087 | 0.121 | 0.616 | 0.757 |
| N,N-Diethylglycine | -0.021 | 0.022 | 1.087 | 0.121 | 0.649 | 0.080 |
| Urocanate | 0.084 | -0.091 | 1.089 | 0.123 | 0.311 | 0.693 |
| Pidolic acid | 0.058 | -0.062 | 1.089 | 0.123 | 0.453 | 0.308 |
| 5α-Pregnan-20α-ol-3-one | 0.038 | -0.041 | 1.090 | 0.125 | 0.734 | 0.798 |
| Mezlocillin | -0.010 | 0.011 | 1.094 | 0.129 | 0.869 | 0.078 |
| Phenylalanyl-Isoleucine | 0.064 | -0.069 | 1.094 | 0.130 | 0.538 | 0.800 |
| cis-1,2-Dihydroxy-8-carboxynaphthalene | 0.007 | -0.008 | 1.095 | 0.131 | 0.966 | 0.835 |
| 11β-Hydroxyprogesterone | -0.033 | 0.036 | 1.101 | 0.138 | 0.640 | 0.248 |
| 3-Amino-5-hydroxybenzoate | -0.042 | 0.045 | 1.102 | 0.141 | 0.420 | 0.198 |
| Nitrazepam | 0.145 | -0.156 | 1.105 | 0.144 | 0.231 | 0.955 |
| Pinidine | -0.047 | 0.051 | 1.105 | 0.144 | 0.270 | 0.146 |
| Stearaldehyde | -0.016 | 0.017 | 1.106 | 0.145 | 0.832 | 0.553 |
| PtdIns-(3,4,5)-P3 (1,2-dioctanoyl) | -0.045 | 0.048 | 1.106 | 0.145 | 0.404 | 0.217 |
| Lucidenic acid A | -0.123 | 0.132 | 1.107 | 0.146 | 0.272 | 1.221 |
| Piperazine | -0.049 | 0.052 | 1.107 | 0.146 | 0.267 | 0.190 |
| 4,4'-Diapolycopenedial | 0.018 | -0.019 | 1.111 | 0.151 | 0.929 | 1.705 |
| Mitiglinide | 0.162 | -0.174 | 1.112 | 0.153 | 0.233 | 1.306 |
| 3-Hydroxy-2-methylpropylidene | -0.058 | 0.063 | 1.113 | 0.154 | 0.269 | 0.105 |
| Protoemetine | 0.006 | -0.006 | 1.113 | 0.154 | 0.970 | 0.769 |
| 3-Ethylpentadecan-2-one | -0.057 | 0.062 | 1.115 | 0.157 | 0.454 | 0.832 |
| Trihydroxystigmastan-6-one | -0.113 | 0.121 | 1.118 | 0.161 | 0.322 | 1.238 |
| 1,7-Dimethylguanosine | -0.128 | 0.137 | 1.118 | 0.161 | 0.238 | 0.854 |
| N,N-Dimethylphenethylamine | -0.042 | 0.045 | 1.121 | 0.165 | 0.341 | 0.094 |
| Methandriol dipropionate | -0.187 | 0.201 | 1.123 | 0.168 | 0.245 | 1.166 |
| Piperideine | -0.039 | 0.042 | 1.124 | 0.168 | 0.413 | 0.104 |
| ent-Isokaurene | -0.044 | 0.047 | 1.124 | 0.168 | 0.375 | 0.183 |
| 4-Methylthio-2-oxobutanoic acid | -0.031 | 0.033 | 1.124 | 0.168 | 0.622 | 0.194 |
| Leu Thr Leu | 0.291 | -0.312 | 1.124 | 0.169 | 0.145 | 1.370 |
| 29-demethylgeodisterol-O-sulfite | -0.076 | 0.081 | 1.124 | 0.169 | 0.223 | 0.445 |
| (R)-4-Hydroxymandelate | -0.147 | 0.158 | 1.125 | 0.169 | 0.463 | 1.260 |
| Coenzyme F420-1 | -0.038 | 0.041 | 1.125 | 0.170 | 0.458 | 0.180 |
| N-Ethylglycine | -0.049 | 0.053 | 1.126 | 0.171 | 0.572 | 0.822 |
| Estrone glucuronide | 0.100 | -0.108 | 1.128 | 0.173 | 0.507 | 0.854 |
| (25S)-5α-cholestan,26-heptol | -0.015 | 0.016 | 1.128 | 0.174 | 0.819 | 0.569 |
| Geranylgeranyl-glycerol phosphate | 0.132 | -0.142 | 1.130 | 0.176 | 0.531 | 1.794 |
| Val Arg Arg Lys | -0.047 | 0.051 | 1.130 | 0.176 | 0.335 | 0.354 |
| Flecainide meta-O-dealkylated | -0.056 | 0.060 | 1.131 | 0.178 | 0.483 | 0.575 |
| 1-Hexadecylamine | -0.026 | 0.028 | 1.133 | 0.180 | 0.611 | 0.151 |
| Glu Ile Ile Ile | -0.016 | 0.017 | 1.136 | 0.184 | 0.800 | 0.107 |
| N1-Acetylspermine | -0.074 | 0.080 | 1.137 | 0.185 | 0.103 | 0.340 |
| Uracil | 0.122 | -0.132 | 1.138 | 0.186 | 0.323 | 0.982 |
| 4-Fluoromuconolactone | -0.057 | 0.061 | 1.140 | 0.189 | 0.646 | 0.608 |
| Methylisoeugenol | -0.065 | 0.070 | 1.141 | 0.191 | 0.301 | 0.249 |
| Tetradecan-3-one | -0.039 | 0.042 | 1.142 | 0.192 | 0.444 | 0.140 |
| Borreverine | -0.119 | 0.128 | 1.142 | 0.192 | 0.392 | 1.094 |
| Riesling acetal | -0.006 | 0.006 | 1.142 | 0.192 | 0.928 | 0.141 |
| 2,4-Dinitrophenol | -0.086 | 0.093 | 1.143 | 0.193 | 0.133 | 0.416 |
| 3-Dimethylallyl-4-hydroxybenzoate | -0.074 | 0.080 | 1.143 | 0.193 | 0.126 | 0.402 |
| 5'-Deoxyadenosine | 0.120 | -0.129 | 1.144 | 0.194 | 0.348 | 0.813 |
| 7-N,N-Dimethylamino-pentathiocyclooctane | -0.116 | 0.124 | 1.144 | 0.195 | 0.467 | 1.077 |
| Canavalioside | 0.183 | -0.197 | 1.146 | 0.197 | 0.300 | 1.358 |
| Tyr Lys Leu | 0.183 | -0.196 | 1.148 | 0.199 | 0.348 | 1.342 |
| Neurosporaxanthin | -0.103 | 0.111 | 1.149 | 0.201 | 0.363 | 1.230 |
| D-Ribose 1-diphosphate | 0.019 | -0.021 | 1.151 | 0.203 | 0.897 | 0.828 |
| Quassin | 0.016 | -0.017 | 1.151 | 0.203 | 0.904 | 0.342 |
| N2'-Acetylgentamicin C1a | -0.104 | 0.111 | 1.151 | 0.203 | 0.345 | 0.237 |
| 3-Tetradecene | -0.068 | 0.073 | 1.152 | 0.204 | 0.121 | 0.336 |
| Asp Met Thr Tyr | 0.009 | -0.010 | 1.152 | 0.204 | 0.876 | 0.301 |
| Helinorbisabone | -0.072 | 0.077 | 1.152 | 0.204 | 0.151 | 0.401 |
| Tetradecan-3-one | 0.012 | -0.013 | 1.152 | 0.204 | 0.928 | 0.520 |
| Geosmin | -0.036 | 0.039 | 1.152 | 0.205 | 0.503 | 0.161 |
| S 1033 | -0.058 | 0.062 | 1.154 | 0.206 | 0.242 | 0.288 |
| Pirimicarb | -0.034 | 0.036 | 1.156 | 0.209 | 0.559 | 0.147 |
| 6?-Methylprednisolone | -0.148 | 0.159 | 1.156 | 0.209 | 0.238 | 0.970 |
| Isophosphamide mustard | -0.126 | 0.136 | 1.156 | 0.209 | 0.276 | 0.782 |
| (1R,2R,4S)-Limonene-1,2-diol | -0.003 | 0.003 | 1.156 | 0.210 | 0.956 | 0.497 |
| 1,3,5-Trihydroxy-10-methylacridone | -0.277 | 0.297 | 1.159 | 0.213 | 0.113 | 0.972 |
| Kelampayoside A | 0.357 | -0.383 | 1.160 | 0.215 | 0.045 | 2.034 |
| Vanillic acid 4-sulfate | 0.009 | -0.010 | 1.162 | 0.216 | 0.957 | 0.583 |
| Penicillin G | -0.041 | 0.044 | 1.162 | 0.217 | 0.827 | 1.723 |
| Verimol D | 0.160 | -0.172 | 1.163 | 0.217 | 0.354 | 1.324 |
| Cloβsol propionate | 0.001 | -0.001 | 1.163 | 0.218 | 0.995 | 1.434 |
| Polyporusterone F | 0.088 | -0.095 | 1.163 | 0.218 | 0.448 | 0.873 |
| Arg Pro Leu Ala | -0.069 | 0.074 | 1.165 | 0.220 | 0.659 | 1.621 |
| Isonicotinic acid | 0.044 | -0.048 | 1.165 | 0.220 | 0.678 | 0.904 |
| 6-Tridecene | -0.096 | 0.103 | 1.169 | 0.225 | 0.060 | 0.454 |
| 3,4-Dihydroxystyrene | -0.049 | 0.053 | 1.169 | 0.225 | 0.776 | 1.305 |
| (6E)-8-Oxogeranial | 0.082 | -0.088 | 1.170 | 0.226 | 0.565 | 0.533 |
| PC(O-16:0/2:0) | -0.162 | 0.174 | 1.173 | 0.231 | 0.186 | 1.266 |
| MG(18:0/0:0/0:0) | -0.026 | 0.028 | 1.176 | 0.234 | 0.641 | 0.170 |
| N-palmitoyl tryptophan | -0.020 | 0.022 | 1.177 | 0.235 | 0.900 | 1.525 |
| Val Arg Arg Gly | -0.030 | 0.033 | 1.177 | 0.235 | 0.617 | 0.105 |
| D-Methionine | 0.058 | -0.062 | 1.179 | 0.237 | 0.577 | 0.819 |
| Paraquat | 0.049 | -0.052 | 1.182 | 0.241 | 0.443 | 0.386 |
| Nebramycin factor 5' | 0.260 | -0.279 | 1.182 | 0.241 | 0.072 | 1.331 |
| Tannin | 0.091 | -0.097 | 1.183 | 0.242 | 0.529 | 0.577 |
| Elaeokanine C | -0.078 | 0.084 | 1.185 | 0.244 | 0.076 | 0.308 |
| Sorbitan palmitate | -0.054 | 0.058 | 1.185 | 0.245 | 0.345 | 0.256 |
| N(α)-t-Butoxycarbonyl-L-leucine | -0.047 | 0.050 | 1.189 | 0.250 | 0.698 | 0.888 |
| Lysyl-Phenylalanine | -0.032 | 0.035 | 1.190 | 0.250 | 0.598 | 0.085 |
| L-rhamnopyranosyl-3-hydroxydecanoic acid | 0.058 | -0.062 | 1.190 | 0.251 | 0.744 | 1.228 |
| β-Alanyl-L-lysine | -0.120 | 0.129 | 1.191 | 0.252 | 0.248 | 0.517 |
| PC(O-16:0/0:0) | -0.036 | 0.038 | 1.194 | 0.255 | 0.783 | 0.741 |
| Sinapine | -0.040 | 0.043 | 1.194 | 0.256 | 0.520 | 0.090 |
| Piperocaine | -0.021 | 0.023 | 1.195 | 0.257 | 0.862 | 0.326 |
| Val Thr Arg Arg | -0.037 | 0.039 | 1.198 | 0.260 | 0.558 | 0.097 |
| (22R,23R)-22,23-Dihydroxy-campest-4-en-3-one | -0.065 | 0.069 | 1.198 | 0.261 | 0.411 | 0.746 |
| Succinic aldehyde | -0.219 | 0.235 | 1.199 | 0.262 | 0.116 | 0.996 |
| 1-(3,4-Dihydroxyphenyl)-1-decene-3,5-dione | -0.085 | 0.091 | 1.199 | 0.262 | 0.101 | 0.318 |
| Urobilin | -0.210 | 0.226 | 1.201 | 0.264 | 0.226 | 1.838 |
| Hercynine | -0.062 | 0.067 | 1.202 | 0.266 | 0.277 | 0.243 |
| (S)-Acetoin | 0.052 | -0.056 | 1.203 | 0.267 | 0.631 | 0.656 |
| BAY-60-7550 | -0.159 | 0.170 | 1.203 | 0.267 | 0.197 | 0.841 |
| Halaminol A | -0.067 | 0.072 | 1.205 | 0.269 | 0.226 | 0.278 |
| 7-Methyloctyl (Z)-4-decenoate | -0.044 | 0.047 | 1.206 | 0.270 | 0.610 | 0.277 |
| Oleamide | -0.008 | 0.008 | 1.208 | 0.273 | 0.953 | 0.331 |
| Thiodiacetic acid sulfoxide | 0.106 | -0.114 | 1.210 | 0.274 | 0.491 | 0.701 |
| Benoxinate | 0.020 | -0.022 | 1.210 | 0.275 | 0.786 | 0.244 |
| Dinoterb | -0.114 | 0.122 | 1.211 | 0.276 | 0.031 | 0.544 |
| Propidium | 0.054 | -0.058 | 1.211 | 0.276 | 0.620 | 0.600 |
| N-(2-Methylpropyl)acetamide | -0.062 | 0.066 | 1.213 | 0.279 | 0.233 | 0.165 |
| 2,5-Dihydro-4,5-dimethyl-2-(2-methylpropyl)thiazole | -0.090 | 0.097 | 1.214 | 0.279 | 0.069 | 0.556 |
| Glu Lys Met Arg | -0.076 | 0.081 | 1.215 | 0.281 | 0.696 | 1.356 |
| Leu Ala Glu Lys | -0.052 | 0.056 | 1.215 | 0.281 | 0.400 | 0.152 |
| Toluene-cis-dihydrodiol | -0.105 | 0.113 | 1.216 | 0.282 | 0.441 | 0.912 |
| Isorenieratene | -0.147 | 0.158 | 1.220 | 0.287 | 0.184 | 0.340 |
| Anhydrorhodovibrin | -0.094 | 0.101 | 1.220 | 0.287 | 0.513 | 0.669 |
| Angoletin | 0.173 | -0.185 | 1.222 | 0.289 | 0.237 | 1.025 |
| Lys Pro Trp | -0.047 | 0.051 | 1.223 | 0.290 | 0.793 | 1.377 |
| Cholesterol sulfate | -0.074 | 0.080 | 1.223 | 0.291 | 0.206 | 0.529 |
| Alamarine | -0.048 | 0.052 | 1.224 | 0.292 | 0.797 | 1.407 |
| Karakoline | 0.037 | -0.039 | 1.225 | 0.292 | 0.804 | 0.771 |
| (25S)-trihydroxy-5β-cholestan-26-oic acid | 0.022 | -0.024 | 1.225 | 0.293 | 0.842 | 0.973 |
| MK 886 | -0.099 | 0.106 | 1.226 | 0.294 | 0.562 | 0.986 |
| Isoleucyl-Alanine | 0.006 | -0.006 | 1.227 | 0.295 | 0.943 | 0.397 |
| cis-1,2-Dihydroxy-1,2-dihydro-7-methylnaphthalene | -0.068 | 0.074 | 1.227 | 0.295 | 0.236 | 0.274 |
| 3,4-DMMA | -0.065 | 0.070 | 1.229 | 0.297 | 0.190 | 0.324 |
| Ganodermic acid TQ | 0.063 | -0.068 | 1.230 | 0.298 | 0.554 | 0.965 |
| Tropate | -0.106 | 0.114 | 1.230 | 0.299 | 0.574 | 1.468 |
| Dehydroxymethylflazine | -0.264 | 0.284 | 1.232 | 0.302 | 0.072 | 1.336 |
| Miraxanthin-V | -0.113 | 0.121 | 1.235 | 0.305 | 0.529 | 0.431 |
| PHOME | 0.041 | -0.044 | 1.236 | 0.305 | 0.694 | 0.423 |
| Oxprenolol | -0.114 | 0.122 | 1.236 | 0.305 | 0.089 | 0.478 |
| L-Citrulline | -0.013 | 0.014 | 1.237 | 0.307 | 0.943 | 1.512 |
| 6α-Hydroxy-castasterone | -0.136 | 0.147 | 1.240 | 0.310 | 0.186 | 1.113 |
| 1-Dodecene | -0.084 | 0.090 | 1.242 | 0.313 | 0.108 | 0.290 |
| Decylubiquinone | -0.059 | 0.063 | 1.244 | 0.315 | 0.393 | 0.543 |
| propionyl butyrate | -0.200 | 0.214 | 1.247 | 0.318 | 0.101 | 1.162 |
| Glycocholate | 0.046 | -0.050 | 1.250 | 0.322 | 0.814 | 1.303 |
| 3β-Fluoro-5α-androstan-17β-ol | -0.090 | 0.096 | 1.251 | 0.323 | 0.087 | 0.428 |
| Tyr-Tyr-OH | 0.164 | -0.176 | 1.253 | 0.326 | 0.299 | 0.807 |
| PA(P-20:0/19:1(9Z)) | 0.074 | -0.079 | 1.253 | 0.326 | 0.731 | 2.176 |
| Germacrene A acid | -0.117 | 0.126 | 1.255 | 0.327 | 0.015 | 0.539 |
| Hydroxysintaxanthin 5,6-epoxide | 0.004 | -0.004 | 1.256 | 0.329 | 0.982 | 1.441 |
| Thymidine | -0.038 | 0.040 | 1.256 | 0.329 | 0.626 | 0.483 |
| Polidocanol | -0.103 | 0.111 | 1.257 | 0.330 | 0.026 | 0.443 |
| 2,3-Dihydroisogedunin | 0.179 | -0.192 | 1.260 | 0.333 | 0.217 | 1.072 |
| Urocortisol | -0.114 | 0.122 | 1.260 | 0.334 | 0.060 | 0.593 |
| Menadione sulfonate | -0.117 | 0.126 | 1.262 | 0.336 | 0.015 | 0.553 |
| N-Methylpelletierine | 0.034 | -0.036 | 1.268 | 0.343 | 0.739 | 0.236 |
| PG(20:2(11Z,14Z)/0:0) | -0.025 | 0.027 | 1.272 | 0.347 | 0.845 | 0.902 |
| Methyl β-D-galactoside | -0.099 | 0.107 | 1.275 | 0.351 | 0.189 | 0.429 |
| Leucyl-Isoleucine | -0.043 | 0.046 | 1.278 | 0.354 | 0.735 | 0.890 |
| Valyl-Leucine | -0.037 | 0.040 | 1.279 | 0.354 | 0.657 | 0.468 |
| Polyoxyethylene 40 monostearate | -0.047 | 0.051 | 1.280 | 0.356 | 0.566 | 0.529 |
| Adenine | -0.196 | 0.210 | 1.282 | 0.358 | 0.246 | 1.302 |
| Calystegin A3 | 0.132 | -0.142 | 1.282 | 0.359 | 0.445 | 1.177 |
| 2S-aminoheptanoic acid | -0.101 | 0.109 | 1.282 | 0.359 | 0.056 | 0.417 |
| PE(15:0/0:0) | -0.083 | 0.089 | 1.283 | 0.360 | 0.287 | 0.690 |
| Farnesylcysteine | -0.026 | 0.028 | 1.290 | 0.367 | 0.836 | 0.630 |
| TyrMe-TyrMe-OH | 0.192 | -0.207 | 1.290 | 0.367 | 0.157 | 1.088 |
| Kiwiionoside | -0.011 | 0.012 | 1.292 | 0.369 | 0.946 | 0.669 |
| Tyr Met Cys Cys | -0.132 | 0.141 | 1.297 | 0.375 | 0.045 | 0.440 |
| MGDG(18:3(9Z,12Z,15Z)/18:4(6Z,9Z,12Z,15Z)) | -0.006 | 0.006 | 1.297 | 0.376 | 0.974 | 0.343 |
| Leu Ser Val | -0.095 | 0.102 | 1.298 | 0.376 | 0.127 | 0.532 |
| 3-(3'-Methylthio)propylmalic acid | -0.133 | 0.143 | 1.301 | 0.379 | 0.004 | 0.683 |
| 25-dihydroxy-19-nor-22-oxavitamin D3 | 0.019 | -0.020 | 1.304 | 0.383 | 0.924 | 1.958 |
| 6β,7β-Dihydroxykaurenoic acid | -0.013 | 0.014 | 1.305 | 0.384 | 0.924 | 1.182 |
| Serinyl-Serine | 0.036 | -0.039 | 1.306 | 0.385 | 0.756 | 0.538 |
| (Z)-7-Dodecenyl propionate | -0.120 | 0.129 | 1.310 | 0.389 | 0.044 | 0.669 |
| I-Urobilinogen | -0.048 | 0.051 | 1.311 | 0.390 | 0.756 | 1.003 |
| Undecyl isobutyrate | -0.012 | 0.013 | 1.311 | 0.390 | 0.873 | 0.656 |
| 2S,3R-dihydroxynonanoic acid | -0.108 | 0.116 | 1.313 | 0.392 | 0.514 | 1.578 |
| 7,8-Dehydro-β-micropteroxanthin | -0.091 | 0.098 | 1.316 | 0.396 | 0.093 | 0.307 |
| Convicine | 0.024 | -0.026 | 1.317 | 0.397 | 0.837 | 0.378 |
| trans-β-damascenone | -0.144 | 0.155 | 1.318 | 0.398 | 0.009 | 0.798 |
| Anabsin | 0.001 | -0.001 | 1.321 | 0.402 | 0.995 | 1.783 |
| Ethyl 3-iodo-2E-acrylate | -0.142 | 0.152 | 1.321 | 0.402 | 0.005 | 0.822 |
| L-threo-3-Methylaspartate | -0.113 | 0.121 | 1.323 | 0.403 | 0.207 | 0.874 |
| 2-Hydroxy-2,4-pentadienoate | -0.116 | 0.125 | 1.323 | 0.404 | 0.079 | 0.608 |
| His Met Lys | -0.126 | 0.135 | 1.323 | 0.404 | 0.386 | 1.084 |
| 13,14-dihydroxy-docosanoic acid | -0.344 | 0.369 | 1.324 | 0.405 | 0.026 | 1.889 |
| PA(O-20:0/18:3(6Z,9Z,12Z)) | -0.097 | 0.105 | 1.327 | 0.408 | 0.541 | 0.279 |
| Octopamine | -0.136 | 0.146 | 1.331 | 0.412 | 0.054 | 0.857 |
| Cinitapride | -0.127 | 0.137 | 1.332 | 0.413 | 0.009 | 0.606 |
| Bolegrevilol | 0.008 | -0.008 | 1.332 | 0.414 | 0.957 | 0.805 |
| Costunolide | -0.105 | 0.113 | 1.333 | 0.415 | 0.140 | 0.627 |
| Senecionine | -0.068 | 0.073 | 1.335 | 0.417 | 0.389 | 0.771 |
| 3-(3,4-Dihydroxyphenyl)pyruvate | -0.101 | 0.109 | 1.339 | 0.421 | 0.560 | 0.837 |
| 1,2-ditetradecanoyl-sn-glycero-3-phosphosulfocholine | -0.112 | 0.120 | 1.342 | 0.424 | 0.055 | 0.547 |
| (22R,23R)-22,23-Dihydroxycampesterol | -0.095 | 0.102 | 1.343 | 0.425 | 0.415 | 1.044 |
| Glycerol 1-dodecanoate 2-tetradecanoate 3-octanoate | 0.104 | -0.112 | 1.345 | 0.428 | 0.367 | 0.839 |
| β-D-Glc-Glc-1,3-octanediol | 0.104 | -0.112 | 1.346 | 0.429 | 0.402 | 0.675 |
| 8Z-Pentadecenyl acetate | -0.046 | 0.049 | 1.347 | 0.429 | 0.586 | 0.280 |
| Prostaglandin E2-biotin | -0.109 | 0.117 | 1.347 | 0.430 | 0.064 | 0.483 |
| Bisphenol A glycidylmethacrylate | -0.137 | 0.147 | 1.350 | 0.433 | 0.403 | 1.450 |
| N,N-Diethylglycine | 0.022 | -0.024 | 1.353 | 0.436 | 0.810 | 0.199 |
| Cys Asp Phe Arg | -0.040 | 0.043 | 1.354 | 0.437 | 0.813 | 1.327 |
| 5-Pyridoxolactone | -0.091 | 0.098 | 1.359 | 0.442 | 0.618 | 1.036 |
| (-)-Epigallocatechin | 0.143 | -0.153 | 1.361 | 0.445 | 0.356 | 0.747 |
| Stigmatellin A | -0.098 | 0.105 | 1.361 | 0.445 | 0.490 | 0.785 |
| 6Z-Octene-2,4-diynoic acid | -0.158 | 0.170 | 1.364 | 0.448 | 0.001 | 0.857 |
| 7Z-Octadecen-11-one | -0.041 | 0.044 | 1.364 | 0.448 | 0.511 | 0.252 |
| Perflubron | -0.158 | 0.170 | 1.366 | 0.450 | 0.003 | 0.886 |
| Ganolucidic acid A | 0.050 | -0.054 | 1.367 | 0.451 | 0.589 | 0.439 |
| Cassiachromone | -0.101 | 0.109 | 1.368 | 0.452 | 0.108 | 0.547 |
| Versiconal | -0.141 | 0.152 | 1.369 | 0.453 | 0.003 | 0.587 |
| Vitamin D3 sulfoconjugate | -0.063 | 0.067 | 1.371 | 0.455 | 0.411 | 0.716 |
| Scopolamine N-oxide | -0.027 | 0.029 | 1.376 | 0.460 | 0.881 | 1.358 |
| Avenanthramide A2 | -0.075 | 0.080 | 1.378 | 0.463 | 0.712 | 1.968 |
| 2,4-Diamino-6-hydroxylaminotoluene | 0.164 | -0.176 | 1.381 | 0.466 | 0.320 | 1.068 |
| Homoarecoline | -0.132 | 0.142 | 1.381 | 0.466 | 0.033 | 0.722 |
| (E)-3,7-Dimethyl-2,6-octadienyl dodecanoate | -0.018 | 0.020 | 1.384 | 0.469 | 0.813 | 0.274 |
| Asp Ile Asp | 0.032 | -0.034 | 1.384 | 0.469 | 0.869 | 1.422 |
| 5-Pentacosyl-1,3-benzenediol | 0.148 | -0.159 | 1.391 | 0.476 | 0.246 | 1.153 |
| (3R,7R)-1,3,7-Octanetriol | -0.069 | 0.075 | 1.391 | 0.476 | 0.346 | 0.479 |
| Leu Lys Lys Glu | -0.013 | 0.014 | 1.391 | 0.477 | 0.915 | 0.275 |
| 16-Hexadecanolide | -0.088 | 0.094 | 1.397 | 0.483 | 0.206 | 0.684 |
| 2-Aminoadenosine | -0.093 | 0.100 | 1.397 | 0.483 | 0.621 | 1.039 |
| 8-Methylthiooctanaldoxime | -0.132 | 0.142 | 1.398 | 0.483 | 0.017 | 0.670 |
| 16?-Hydroxy-17-epistanozolol | -0.004 | 0.004 | 1.399 | 0.484 | 0.968 | 0.726 |
| Lys Arg Ser Tyr | 0.065 | -0.070 | 1.400 | 0.485 | 0.618 | 0.679 |
| Glu Arg Arg Thr | -0.154 | 0.166 | 1.401 | 0.486 | 0.220 | 0.892 |
| Ladderane-OCA-Glycerol | -0.031 | 0.033 | 1.402 | 0.488 | 0.878 | 2.057 |
| Pyridine | -0.170 | 0.182 | 1.405 | 0.491 | 0.118 | 1.057 |
| Tiliroside | -0.125 | 0.135 | 1.407 | 0.493 | 0.018 | 0.564 |
| 4-Hydroxy-5-phenyltetrahydro-1,3-oxazin-2-one | 0.094 | -0.101 | 1.409 | 0.495 | 0.516 | 1.000 |
| Pro Gln Leu | 0.159 | -0.171 | 1.409 | 0.495 | 0.239 | 0.758 |
| Reticulataxanthin | -0.072 | 0.077 | 1.409 | 0.495 | 0.564 | 0.774 |
| Norverapamil | -0.011 | 0.012 | 1.414 | 0.499 | 0.939 | 1.177 |
| 4-Hydroxyphenylglyoxylate | -0.175 | 0.188 | 1.415 | 0.500 | 0.018 | 1.017 |
| Tenovin-6 | -0.017 | 0.019 | 1.418 | 0.504 | 0.878 | 0.965 |
| Versiconol acetate | -0.141 | 0.151 | 1.418 | 0.504 | 0.164 | 0.835 |
| 2-chlorohexadecanol | 0.037 | -0.040 | 1.420 | 0.505 | 0.815 | 0.369 |
| (E,E)-3,7,11-Trimethyl-2,6,10-dodecatrienyl propionate | -0.087 | 0.093 | 1.421 | 0.506 | 0.339 | 0.669 |
| SQDG(16:0/16:0) | -0.181 | 0.195 | 1.424 | 0.510 | 0.367 | 0.963 |
| trans,trans-Farnesyl phosphate | -0.128 | 0.137 | 1.436 | 0.522 | 0.346 | 1.576 |
| PS(O-16:0/0:0) | -0.139 | 0.150 | 1.443 | 0.529 | 0.467 | 1.244 |
| Duartin (-) | -0.156 | 0.168 | 1.446 | 0.532 | 0.444 | 1.729 |
| Pro Cys Cys | -0.232 | 0.249 | 1.449 | 0.535 | 0.225 | 1.417 |
| Cucurbitacin S | 0.042 | -0.045 | 1.450 | 0.536 | 0.697 | 0.786 |
| (S)-1-Phenylethanol | -0.089 | 0.095 | 1.452 | 0.538 | 0.637 | 1.289 |
| Isolithocholate | -0.070 | 0.076 | 1.457 | 0.543 | 0.654 | 1.521 |
| Dipropyl hexanedioate | 0.034 | -0.036 | 1.458 | 0.544 | 0.615 | 0.460 |
| Kolanone | -0.131 | 0.141 | 1.461 | 0.547 | 0.356 | 0.617 |
| 2-oxo-heneicosanoic acid | -0.079 | 0.085 | 1.463 | 0.549 | 0.276 | 0.511 |
| Plaunotol | -0.019 | 0.021 | 1.464 | 0.550 | 0.841 | 0.763 |
| Thiothece-474 | -0.118 | 0.127 | 1.467 | 0.552 | 0.026 | 0.555 |
| 3-Methylbutyl pentadecanoate | -0.012 | 0.013 | 1.475 | 0.561 | 0.886 | 0.320 |
| Valyl-Valine | -0.007 | 0.008 | 1.485 | 0.570 | 0.931 | 0.452 |
| 1H-Imidazole-4-methanol | -0.184 | 0.197 | 1.486 | 0.572 | 0.001 | 0.943 |
| BL II | -0.168 | 0.180 | 1.488 | 0.574 | 0.001 | 0.730 |
| AminoDHQ | -0.205 | 0.220 | 1.489 | 0.574 | 0.261 | 1.118 |
| Gln His Trp | -0.026 | 0.027 | 1.491 | 0.577 | 0.810 | 0.177 |
| Bufanolide skeleton | -0.025 | 0.027 | 1.502 | 0.587 | 0.851 | 1.378 |
| N6-Acetyl-L-lysine | 0.061 | -0.065 | 1.503 | 0.588 | 0.578 | 0.748 |
| Austalide G | -0.214 | 0.230 | 1.505 | 0.590 | 0.061 | 1.129 |
| 5-Ethylundecan-6-one | 0.015 | -0.016 | 1.508 | 0.593 | 0.846 | 0.316 |
| Simvastatin | -0.008 | 0.009 | 1.512 | 0.596 | 0.936 | 0.818 |
| PA(19:3(10Z,13Z,16Z)/0:0) | -0.084 | 0.090 | 1.512 | 0.597 | 0.613 | 1.641 |
| Cys His Lys Cys | -0.040 | 0.042 | 1.523 | 0.607 | 0.795 | 1.104 |
| D-Urobilin | -0.011 | 0.011 | 1.524 | 0.608 | 0.905 | 0.460 |
| Mequitazine | -0.146 | 0.157 | 1.529 | 0.613 | 0.030 | 0.912 |
| PS(P-16:0/18:3(9Z,12Z,15Z)) | -0.223 | 0.240 | 1.535 | 0.618 | 0.000 | 0.978 |
| 2-oxo-pentadecanoic acid | -0.193 | 0.207 | 1.537 | 0.620 | 0.000 | 0.998 |
| 6α-Hydroxy-3-oxo-5β-cholan-24-oic Acid | -0.159 | 0.170 | 1.544 | 0.627 | 0.070 | 1.227 |
| 16-Hydroxypalmitate | 0.029 | -0.031 | 1.545 | 0.628 | 0.770 | 0.704 |
| 3,3'-Dimethylbenzidine | -0.087 | 0.094 | 1.548 | 0.630 | 0.480 | 0.549 |
| Norselic acid E | -0.294 | 0.315 | 1.548 | 0.630 | 0.143 | 2.037 |
| Gln Leu Lys Lys | 0.029 | -0.032 | 1.550 | 0.632 | 0.863 | 1.519 |
| Tributyl phosphate | 0.069 | -0.074 | 1.550 | 0.633 | 0.599 | 0.847 |
| Epidioxy-Eg-6-one | -0.204 | 0.219 | 1.560 | 0.642 | 0.000 | 0.819 |
| PC(11:0/0:0) | 0.005 | -0.006 | 1.568 | 0.649 | 0.963 | 0.601 |
| Pimelate | -0.092 | 0.099 | 1.570 | 0.650 | 0.221 | 0.771 |
| 11α-(fluoromethyl)-1α,25-dihydroxyvitamin D3* | -0.201 | 0.216 | 1.571 | 0.651 | 0.031 | 1.113 |
| 13E-Docosenamide | 0.011 | -0.012 | 1.571 | 0.652 | 0.939 | 0.344 |
| PG(P-18:0/18:3(9Z,12Z,15Z)) | 0.013 | -0.014 | 1.575 | 0.656 | 0.949 | 2.044 |
| 3β,5β-Ketodiol | -0.010 | 0.011 | 1.583 | 0.663 | 0.932 | 0.358 |
| 2-Propylglutaric acid | -0.157 | 0.168 | 1.593 | 0.672 | 0.011 | 0.842 |
| 7-Hydroxyrisperidone | 0.158 | -0.170 | 1.596 | 0.674 | 0.302 | 1.246 |
| 4-Hydroxy-2-quinolone | -0.180 | 0.194 | 1.602 | 0.680 | 0.205 | 1.820 |
| Acetoxy-Lanostatriene-26-oic acid | -0.204 | 0.219 | 1.604 | 0.682 | 0.160 | 1.334 |
| Hydronitroxide radical* | -0.213 | 0.229 | 1.605 | 0.683 | 0.000 | 1.084 |
| ethyl propionate | 0.005 | -0.005 | 1.607 | 0.685 | 0.975 | 0.699 |
| L-Histidine* | -0.220 | 0.236 | 1.615 | 0.692 | 0.000 | 1.126 |
| 1-Octen-3-yl primeveroside | 0.095 | -0.102 | 1.619 | 0.695 | 0.576 | 1.159 |
| DG(18:4(6Z,9Z,12Z,15Z)/20:3(8Z,11Z,14Z)/0:0) | 0.047 | -0.050 | 1.624 | 0.699 | 0.766 | 1.187 |
| Hydnocarpic acid* | -0.171 | 0.183 | 1.631 | 0.706 | 0.021 | 1.022 |
| Pro His Pro | -0.179 | 0.192 | 1.638 | 0.712 | 0.293 | 1.724 |
| 9Z-Dodecen-7-ynyl acetate | -0.127 | 0.136 | 1.644 | 0.717 | 0.045 | 0.503 |
| 17-hydroxy-heptadecanoic acid | -0.056 | 0.061 | 1.644 | 0.717 | 0.682 | 0.881 |
| PG(22:2(13Z,16Z)/0:0) | 0.099 | -0.106 | 1.645 | 0.718 | 0.473 | 1.035 |
| Ethylmethylmaleimide* | -0.257 | 0.276 | 1.651 | 0.723 | 0.033 | 1.748 |
| 16β-Hydroxy-Oleanadien-28-oic acid | 0.042 | -0.046 | 1.660 | 0.731 | 0.751 | 0.739 |
| all-trans-Carophyll yellow | -0.143 | 0.154 | 1.662 | 0.733 | 0.016 | 0.782 |
| Met Gly Asn | -0.136 | 0.146 | 1.685 | 0.752 | 0.406 | 0.855 |
| 4-Formyl Indole | -0.136 | 0.146 | 1.685 | 0.753 | 0.066 | 0.838 |
| Deoxyinosine | 0.009 | -0.009 | 1.692 | 0.758 | 0.962 | 0.801 |
| N-Methyl-L-glutamate | 0.003 | -0.003 | 1.700 | 0.765 | 0.989 | 0.540 |
| 2-Methylserine | -0.261 | 0.281 | 1.702 | 0.767 | 0.124 | 1.752 |
| 2-n-Propyl-3-pentenoic acid | -0.174 | 0.187 | 1.706 | 0.771 | 0.006 | 0.932 |
| Estradiol-17β 3-glucuronide | -0.042 | 0.045 | 1.715 | 0.778 | 0.784 | 1.203 |
| Eremosulphoxinolide A | -0.145 | 0.155 | 1.715 | 0.778 | 0.433 | 1.333 |
| 3β,9α-Dihydroxy-11-oxo-5β-cholan-24-oic Acid | -0.086 | 0.093 | 1.726 | 0.787 | 0.526 | 0.979 |
| (R)-3-Hydroxy-3-methyl-2-oxopentanoate | -0.122 | 0.131 | 1.730 | 0.791 | 0.122 | 0.867 |
| Butanal(+)* | -0.252 | 0.270 | 1.731 | 0.792 | 0.001 | 1.304 |
| PS(16:0/18:1(9Z)) | 0.331 | -0.355 | 1.732 | 0.792 | 0.069 | 1.803 |
| 14,20-Epoxy-17-hydroxy-1-oxowitha-3,5,24-trienolide | 0.007 | -0.008 | 1.736 | 0.796 | 0.956 | 1.038 |
| 10,16-DHHA* | -0.254 | 0.273 | 1.740 | 0.799 | 0.001 | 1.318 |
| 4,5-Dimethyl-4-hexen-3-one | -0.193 | 0.207 | 1.751 | 0.808 | 0.058 | 1.020 |
| (24S)-24-fluoro-1α,25-dihydroxyvitamin D2* | -0.234 | 0.251 | 1.752 | 0.809 | 0.006 | 1.188 |
| 2-Phenyl-1,3-propanediol monocarbamate | -0.139 | 0.149 | 1.759 | 0.814 | 0.073 | 0.595 |
| 1-Methyl-THBC* | -0.334 | 0.359 | 1.763 | 0.818 | 0.037 | 2.056 |
| Coumaryl acetate | 0.029 | -0.031 | 1.780 | 0.832 | 0.819 | 0.411 |
| PG(18:1(9Z)/0:0) | -0.154 | 0.165 | 1.781 | 0.833 | 0.269 | 0.514 |
| 6-Hydroxyhexanoic acid | -0.084 | 0.091 | 1.786 | 0.837 | 0.200 | 0.401 |
| DG(19:1(9Z)/20:2(11Z,14Z)/0:0) | 0.125 | -0.134 | 1.788 | 0.839 | 0.498 | 1.670 |
| S-(1,2-Dichlorovinyl)-L-cysteine* | -0.244 | 0.262 | 1.790 | 0.840 | 0.000 | 1.322 |
| PA(22:6(4Z,7Z,10Z,13Z,16Z,19Z)/21:0) | -0.011 | 0.011 | 1.861 | 0.896 | 0.956 | 1.943 |
| Probucol | -0.143 | 0.154 | 1.864 | 0.899 | 0.189 | 1.274 |
| (2R)-2-Hydroxy-2-methylbutanenitrile | -0.146 | 0.157 | 1.865 | 0.899 | 0.274 | 1.105 |
| 3-(Methylthio)propionic acid | -0.244 | 0.262 | 1.869 | 0.902 | 0.125 | 1.253 |
| N-Desmethyltolmetin* | -0.299 | 0.321 | 1.870 | 0.903 | 0.000 | 1.285 |
| (S)-Acetoin | -0.252 | 0.271 | 1.870 | 0.903 | 0.188 | 1.516 |
| N,O-Didesmethylverapamil* | -0.274 | 0.294 | 1.877 | 0.908 | 0.039 | 1.938 |
| (E)-2-Methyl-2-butenyl butyrate | -0.121 | 0.130 | 1.881 | 0.911 | 0.094 | 0.874 |
| cis-1,2-Cyclohexanediol | 0.021 | -0.022 | 1.883 | 0.913 | 0.911 | 1.416 |
| Thiobinupharidine | -0.126 | 0.136 | 1.904 | 0.929 | 0.461 | 1.370 |
| Caffeoyl aspartic acid* | -0.364 | 0.391 | 1.911 | 0.934 | 0.013 | 1.707 |
| Acetoxy-6-gingerol | -0.084 | 0.090 | 1.924 | 0.944 | 0.514 | 1.178 |
| Pro Leu Leu Lys* | -0.281 | 0.302 | 1.940 | 0.956 | 0.000 | 1.252 |
| 3,5-Dihydroxy-phenylglycine* | -0.187 | 0.201 | 1.960 | 0.971 | 0.008 | 1.030 |
| Thr Ile Ile Arg | -0.004 | 0.004 | 1.986 | 0.990 | 0.982 | 1.078 |
| 5β-Chola-3,11-dien-24-oic Acid | -0.024 | 0.026 | 1.996 | 0.997 | 0.849 | 0.826 |
| 2-bromopalmitaldehyde* | -0.178 | 0.191 | 2.016 | 1.012 | 0.037 | 1.186 |
| 3-Cresol | -0.198 | 0.212 | 2.019 | 1.014 | 0.321 | 1.815 |
| Aniline* | -0.206 | 0.221 | 2.029 | 1.021 | 0.004 | 1.086 |
| N-Hexadecanoylpyrrolidine | 0.384 | -0.412 | 2.081 | 1.058 | 0.060 | 2.235 |
| MGDG(16:3(7Z,10Z,13Z)/20:5(5Z,8Z,11Z,14Z,17Z)) | -0.082 | 0.088 | 2.088 | 1.062 | 0.619 | 0.576 |
| Fenpyroximate | -0.146 | 0.157 | 2.093 | 1.065 | 0.371 | 1.535 |
| Undecan-4-one | -0.140 | 0.151 | 2.110 | 1.077 | 0.298 | 1.613 |
| Butanal | -0.184 | 0.198 | 2.112 | 1.079 | 0.164 | 0.850 |
| Dimethyl sulfone | -0.131 | 0.141 | 2.122 | 1.085 | 0.397 | 0.772 |
| 3-Propylmalate | -0.026 | 0.028 | 2.126 | 1.088 | 0.864 | 0.151 |
| Hydroxylaminobenzene | -0.252 | 0.271 | 2.146 | 1.102 | 0.116 | 2.010 |
| Sorbitan laurate | 0.075 | -0.081 | 2.160 | 1.111 | 0.544 | 0.535 |
| PG(15:0/0:0) | -0.157 | 0.168 | 2.196 | 1.135 | 0.384 | 2.035 |
| (13E)-Labda-7,13-dien-15-ol | 0.200 | -0.215 | 2.201 | 1.138 | 0.214 | 1.077 |
| (6S)-vitamin D2 6,19-sulfur dioxide adduct | 0.021 | -0.022 | 2.206 | 1.141 | 0.847 | 0.457 |
| PA(P-20:0/0:0) | -0.203 | 0.218 | 2.213 | 1.146 | 0.166 | 1.764 |
| 2-Deoxyecdysone | -0.231 | 0.248 | 2.244 | 1.166 | 0.182 | 1.840 |
| Levonorgestrel acetate | -0.104 | 0.112 | 2.267 | 1.181 | 0.142 | 0.392 |
| Isoamericanol A | -0.166 | 0.178 | 2.285 | 1.192 | 0.282 | 1.719 |
| Dihydroisolysergic acid II | -0.262 | 0.281 | 2.346 | 1.230 | 0.097 | 1.734 |
| N-Acetylmuramate | 0.190 | -0.205 | 2.354 | 1.235 | 0.160 | 1.086 |
| 8-Amino-7-oxononanoate | -0.162 | 0.174 | 2.403 | 1.265 | 0.035 | 0.871 |
| Yangonin | -0.189 | 0.203 | 2.421 | 1.275 | 0.253 | 1.276 |
| Phe Asp Thr* | -0.254 | 0.273 | 2.488 | 1.315 | 0.013 | 1.604 |
| 9-Demethylmunduserone | -0.127 | 0.136 | 2.497 | 1.320 | 0.425 | 1.364 |
| Hercynine* | -0.254 | 0.273 | 2.534 | 1.341 | 0.002 | 1.416 |
| (1R,2R)-3-oxo-2-pentyl-cyclopentanebutanoic acid | 0.019 | -0.020 | 2.573 | 1.364 | 0.907 | 0.798 |
| Diasarone 2 | -0.017 | 0.018 | 2.657 | 1.410 | 0.880 | 0.549 |
| Docosapentaynoic acid | 0.013 | -0.014 | 2.692 | 1.429 | 0.945 | 1.535 |
| 9-Oxononanoic acid | -0.206 | 0.221 | 2.782 | 1.476 | 0.127 | 1.403 |
| Allocholic acid | -0.342 | 0.367 | 2.838 | 1.505 | 0.054 | 2.278 |
| Nirvanol | 0.056 | -0.060 | 2.893 | 1.533 | 0.540 | 0.401 |
| PG(20:1(11Z)/0:0) | -0.053 | 0.057 | 2.945 | 1.558 | 0.800 | 1.224 |
| 3-Carbamoyl-2-phenylpropionic acid | -0.118 | 0.127 | 2.984 | 1.577 | 0.285 | 0.857 |
| Lithocholic acid sulfate* | -0.295 | 0.317 | 3.016 | 1.593 | 0.014 | 1.692 |
| Traumatic acid | -0.175 | 0.188 | 3.027 | 1.598 | 0.310 | 1.096 |
| Ser Val Val Pro | -0.065 | 0.070 | 3.098 | 1.632 | 0.660 | 0.867 |
| UDP-N-acetyl-D-galactosaminuronic acid | -0.194 | 0.208 | 3.128 | 1.645 | 0.010 | 0.866 |
| L-Normetanephrine* | -0.313 | 0.336 | 3.157 | 1.659 | 0.036 | 2.135 |
| Tributyrin* | -0.281 | 0.302 | 3.227 | 1.690 | 0.000 | 1.217 |
| Val Ser Met* | -0.410 | 0.440 | 3.724 | 1.897 | 0.023 | 2.307 |
| PG(16:0/0:0) | -0.092 | 0.098 | 3.760 | 1.911 | 0.240 | 0.465 |
| N-Succinyl-AKP* | -0.553 | 0.594 | 3.952 | 1.982 | 0.002 | 3.134 |
| Pantothenate | 0.025 | -0.027 | 3.989 | 1.996 | 0.897 | 0.848 |
| 15(S)-HPETE | -0.168 | 0.181 | 3.997 | 1.999 | 0.290 | 1.455 |
| (α-2-Hydroxy-4-(methylthio)butanoic acid | -0.015 | 0.017 | 4.618 | 2.207 | 0.908 | 0.723 |
| Candoxatrilat* | -0.362 | 0.389 | 4.803 | 2.264 | 0.042 | 2.169 |
| 2-Nonenyl acetate | -0.058 | 0.062 | 5.097 | 2.350 | 0.667 | 1.039 |
| Cinncassiol A | -0.285 | 0.306 | 6.779 | 2.761 | 0.091 | 1.364 |

^*^ indicates significantly different metabolite

**Table S5.** Sample-specific DNA concentrations

| LC Contract No. | Internal Number | subjectID | specimen type | extraction plate | extraction plate_well_code | template plate | template plate_well_code | primer plate used i7 | primer plate used i5 | ng/ul | actual vol pooled | ng pooled |
| --- | --- | --- | --- | --- | --- | --- | --- | --- | --- | --- | --- | --- |
| LC-P20221204036 | la22l07A427 | H01 | fecal | V3V4-20221207-40 | F3 | V3V4-20221207-40-Y62 | F3 | GACCTACT | TCTCCAGC | 20.10269049 | 0.746168778 | 15 |
| LC-P20221204036 | la22l07A423 | H02 | fecal | V3V4-20221207-40 | B3 | V3V4-20221207-40-Y62 | B3 | GACCTACT | TCCTAGGC | 11.019994 | 1.361162265 | 15 |
| LC-P20221204036 | la22l07A431 | H03 | fecal | V3V4-20221207-40 | B4 | V3V4-20221207-40-Y62 | B4 | ACGAGACG | TCCTAGGC | 11.60441208 | 1.292611801 | 15 |
| LC-P20221204036 | la22l07A433 | H04 | fecal | V3V4-20221207-40 | D4 | V3V4-20221207-40-Y62 | D4 | ACGAGACG | TGGTTCGT | 14.85584307 | 1.009703719 | 15 |
| LC-P20221204036 | la22l07A434 | H05 | fecal | V3V4-20221207-40 | E4 | V3V4-20221207-40-Y62 | E4 | ACGAGACG | CTTGCGTG | 11.58503911 | 1.294773359 | 15 |
| LC-P20221204036 | la22l07A432 | H06 | fecal | V3V4-20221207-40 | C4 | V3V4-20221207-40-Y62 | C4 | ACGAGACG | TTGGTAAC | 14.49259979 | 1.035010986 | 15 |
| LC-P20221204036 | la22l07A435 | H07 | fecal | V3V4-20221207-40 | F4 | V3V4-20221207-40-Y62 | F4 | ACGAGACG | TCTCCAGC | 12.09196529 | 1.240493141 | 15 |
| LC-P20221204036 | la22l07A425 | H08 | fecal | V3V4-20221207-40 | D3 | V3V4-20221207-40-Y62 | D3 | GACCTACT | TGGTTCGT | 17.21934603 | 0.871113222 | 15 |
| LC-P20230902002 | la23i11A78 | H09 | fecal | V3V4-20230911-21 | F10 | V3V4-20230911-21-Y25 | F10 | TGACTTCG | ACATTATC | 10.2376375 | 1.465181786 | 15 |
| LC-P20230902002 | la23i11A79 | H10 | fecal | V3V4-20230911-21 | G10 | V3V4-20230911-21-Y25 | G10 | TGACTTCG | TGGAACAG | 1.964960192 | 7.633742434 | 15 |
| LC-P20230902002 | la23i11A80 | H11 | fecal | V3V4-20230911-21 | H10 | V3V4-20230911-21-Y25 | H10 | TGACTTCG | CCTTGTTA | 7.83680697 | 1.914044847 | 15 |
| LC-P20230902002 | la23i11A81 | H12 | fecal | V3V4-20230911-21 | A11 | V3V4-20230911-21-Y25 | A11 | AATTGTAA | TAGATCGC | 2.540805154 | 5.903640418 | 15 |
| LC-P20230902002 | la23i11A82 | H13 | fecal | V3V4-20230911-21 | B11 | V3V4-20230911-21-Y25 | B11 | AATTGTAA | CTACAAGA | 13.44819464 | 1.115391352 | 15 |
| LC-P20230902002 | la23i11A83 | H14 | fecal | V3V4-20230911-21 | C11 | V3V4-20230911-21-Y25 | C11 | AATTGTAA | TATCCTCT | 11.1625331 | 1.343781009 | 15 |
| LC-P20230902002 | la23i11A85 | H15 | fecal | V3V4-20230911-21 | E11 | V3V4-20230911-21-Y25 | E11 | AATTGTAA | TGCCTGGT | 12.63315254 | 1.187352084 | 15 |
| LC-P20230902002 | la23i11A86 | H16 | fecal | V3V4-20230911-21 | F11 | V3V4-20230911-21-Y25 | F11 | AATTGTAA | ACATTATC | 10.20574455 | 1.469760479 | 15 |
| LC-P20230902002 | la23i11A87 | H17 | fecal | V3V4-20230911-21 | G11 | V3V4-20230911-21-Y25 | G11 | AATTGTAA | TGGAACAG | 2.042920741 | 7.342428759 | 15 |
| LC-P20230902002 | la23i11A88 | H18 | fecal | V3V4-20230911-21 | H11 | V3V4-20230911-21-Y25 | H11 | AATTGTAA | CCTTGTTA | 9.74152492 | 1.539799993 | 15 |
| LC-P20230902002 | la23i11A89 | H19 | fecal | V3V4-20230911-22 | A1 | V3V4-20230911-22-Y26 | A1 | TAGAGCTC | GTTGATAG | 12.90778629 | 1.162089274 | 15 |
| LC-P20230902002 | la23i11A91 | H20 | fecal | V3V4-20230911-22 | C1 | V3V4-20230911-22-Y26 | C1 | TAGAGCTC | CATACACT | 13.88052132 | 1.080651054 | 15 |
| LC-P20230902002 | la23i11A96 | H21 | fecal | V3V4-20230911-22 | H1 | V3V4-20230911-22-Y26 | H1 | TAGAGCTC | AAGGCTAT | 12.37559995 | 1.21206245 | 15 |
| LC-P20230902002 | la23i11A97 | H22 | fecal | V3V4-20230911-22 | A2 | V3V4-20230911-22-Y26 | A2 | ATCCATTA | GTTGATAG | 13.81319176 | 1.085918466 | 15 |
| LC-P20230902002 | la23i11A98 | H23 | fecal | V3V4-20230911-22 | B2 | V3V4-20230911-22-Y26 | B2 | ATCCATTA | TCTCTCCG | 14.90463944 | 1.006398046 | 15 |
| LC-P20230902002 | la23i11A103 | H24 | fecal | V3V4-20230911-22 | G2 | V3V4-20230911-22-Y26 | G2 | ATCCATTA | CTATTAAG | 15.91635474 | 0.942426846 | 15 |
| LC-P20230902002 | la23i11A104 | H25 | fecal | V3V4-20230911-22 | H2 | V3V4-20230911-22-Y26 | H2 | ATCCATTA | AAGGCTAT | 12.09204871 | 1.240484583 | 15 |
| LC-P20230902002 | la23i11A106 | H26 | fecal | V3V4-20230911-22 | B3 | V3V4-20230911-22-Y26 | B3 | TGTTGCAC | TCTCTCCG | 14.64063667 | 1.024545608 | 15 |
| LC-P20230902002 | la23i11A107 | H27 | fecal | V3V4-20230911-22 | C3 | V3V4-20230911-22-Y26 | C3 | TGTTGCAC | CATACACT | 14.53078317 | 1.032291228 | 15 |
| LC-P20230902002 | la23i11A109 | H28 | fecal | V3V4-20230911-22 | E3 | V3V4-20230911-22-Y26 | E3 | TGTTGCAC | ATCACGAA | 14.29335786 | 1.049438497 | 15 |
| LC-P20230902002 | la23i11A112 | H29 | fecal | V3V4-20230911-22 | H3 | V3V4-20230911-22-Y26 | H3 | TGTTGCAC | AAGGCTAT | 10.65956258 | 1.407187198 | 15 |
| LC-P20230902002 | la23i11A113 | H30 | fecal | V3V4-20230911-22 | A4 | V3V4-20230911-22-Y26 | A4 | TGGCTATC | GTTGATAG | 12.18665122 | 1.230854952 | 15 |
| LC-P20230902002 | la23i11A114 | S01 | fecal | V3V4-20230911-22 | B4 | V3V4-20230911-22-Y26 | B4 | TGGCTATC | TCTCTCCG | 15.69841957 | 0.955510199 | 15 |
| LC-P20230902002 | la23i11A116 | S02 | fecal | V3V4-20230911-22 | D4 | V3V4-20230911-22-Y26 | D4 | TGGCTATC | TTCTAGCT | 14.88160564 | 1.007955752 | 15 |
| LC-P20221204036 | la22l07A421 | S03 | fecal | V3V4-20221207-40 | H2 | V3V4-20221207-40-Y62 | H2 | CTAACCTC | GCATCAAC | 20.46754819 | 0.732867457 | 15 |
| LC-P20221204036 | la22l07A419 | S04 | fecal | V3V4-20221207-40 | F2 | V3V4-20221207-40-Y62 | F2 | CTAACCTC | TCTCCAGC | 10.79720479 | 1.389248449 | 15 |
| LC-P20221204036 | la22l07A418 | S05 | fecal | V3V4-20221207-40 | E2 | V3V4-20221207-40-Y62 | E2 | CTAACCTC | CTTGCGTG | 17.02077304 | 0.881276072 | 15 |
| LC-P20221204036 | la22l07A417 | S06 | fecal | V3V4-20221207-40 | D2 | V3V4-20221207-40-Y62 | D2 | CTAACCTC | TGGTTCGT | 16.54290632 | 0.906733056 | 15 |
| LC-P20221204036 | la22l07A414 | S07 | fecal | V3V4-20221207-40 | A2 | V3V4-20221207-40-Y62 | A2 | CTAACCTC | CTGAACGC | 3.95208692 | 3.795463082 | 15 |
| LC-P20221204036 | la22l07A413 | S08 | fecal | V3V4-20221207-40 | H1 | V3V4-20221207-40-Y62 | H1 | TCAGTTCG | GCATCAAC | 16.33949008 | 0.918021305 | 15 |
| LC-P20230902002 | la23i11A119 | S09 | fecal | V3V4-20230911-22 | G4 | V3V4-20230911-22-Y26 | G4 | TGGCTATC | CTATTAAG | 16.99539961 | 0.882591781 | 15 |
| LC-P20230902002 | la23i11A122 | S10 | fecal | V3V4-20230911-22 | B5 | V3V4-20230911-22-Y26 | B5 | CGGTCATA | TCTCTCCG | 15.10308447 | 0.993174608 | 15 |
| LC-P20230902002 | la23i11A125 | S11 | fecal | V3V4-20230911-22 | E5 | V3V4-20230911-22-Y26 | E5 | CGGTCATA | ATCACGAA | 14.83199438 | 1.011327244 | 15 |
| LC-P20230902002 | la23i11A131 | S12 | fecal | V3V4-20230911-22 | C6 | V3V4-20230911-22-Y26 | C6 | GAAGGTTC | CATACACT | 11.71002877 | 1.2809533 | 15 |
| LC-P20230902002 | la23i11A134 | S13 | fecal | V3V4-20230911-22 | F6 | V3V4-20230911-22-Y26 | F6 | GAAGGTTC | GCGTAAGA | 0.607737913 | 24.68169201 | 15 |
| LC-P20230902002 | la23i11A136 | S14 | fecal | V3V4-20230911-22 | H6 | V3V4-20230911-22-Y26 | H6 | GAAGGTTC | AAGGCTAT | 11.48198393 | 1.306394443 | 15 |
| LC-P20230902002 | la23i11A138 | S15 | fecal | V3V4-20230911-22 | B7 | V3V4-20230911-22-Y26 | B7 | ATCTCGCT | TCTCTCCG | 16.81644471 | 0.891984023 | 15 |
| LC-P20230902002 | la23i11A144 | S16 | fecal | V3V4-20230911-22 | H7 | V3V4-20230911-22-Y26 | H7 | ATCTCGCT | AAGGCTAT | 6.310549429 | 2.376972111 | 15 |
| LC-P20230902002 | la23i11A147 | S17 | fecal | V3V4-20230911-22 | C8 | V3V4-20230911-22-Y26 | C8 | AGTTACGG | CATACACT | 11.57891331 | 1.295458356 | 15 |
| LC-P20230902002 | la23i11A148 | S18 | fecal | V3V4-20230911-22 | D8 | V3V4-20230911-22-Y26 | D8 | AGTTACGG | TTCTAGCT | 13.63600869 | 1.100028633 | 15 |
| LC-P20230902002 | la23i11A151 | S19 | fecal | V3V4-20230911-22 | G8 | V3V4-20230911-22-Y26 | G8 | AGTTACGG | CTATTAAG | 12.47014412 | 1.202873026 | 15 |
| LC-P20230902002 | la23i11A153 | S20 | fecal | V3V4-20230911-22 | A9 | V3V4-20230911-22-Y26 | A9 | ATGTAAGA | GTTGATAG | 14.24197477 | 1.053224727 | 15 |
| LC-P20230902002 | la23i11A156 | S21 | fecal | V3V4-20230911-22 | D9 | V3V4-20230911-22-Y26 | D9 | ATGTAAGA | TTCTAGCT | 14.38549306 | 1.042717128 | 15 |
| LC-P20231216041 | la23l19A348 | S22 | fecal | V3V4-20231219-123 | F6 | V3V4-20231219-123-Y29 | F6 | GAAGGTTC | TTGGTCTC | 10.33821845 | 1.450926973 | 15 |
| LC-P20230902002 | la23i11A158 | S23 | fecal | V3V4-20230911-22 | F9 | V3V4-20230911-22-Y26 | F9 | ATGTAAGA | GCGTAAGA | 1.617681385 | 9.272530514 | 15 |
| LC-P20230902002 | la23i11A159 | S24 | fecal | V3V4-20230911-22 | G9 | V3V4-20230911-22-Y26 | G9 | ATGTAAGA | CTATTAAG | 13.74940585 | 1.090956232 | 15 |
| LC-P20231216041 | la23l19A349 | S25 | fecal | V3V4-20231219-123 | G6 | V3V4-20231219-123-Y29 | G6 | GAAGGTTC | AAGTTGGT | 12.80261871 | 1.171635299 | 15 |
| LC-P20231216041 | la23l19A355 | S26 | fecal | V3V4-20231219-123 | E7 | V3V4-20231219-123-Y29 | E7 | ATCTCGCT | GCCTTGTT | 10.42524382 | 1.43881527 | 15 |
| LC-P20230902002 | la23i11A62 | S27 | fecal | V3V4-20230911-21 | F8 | V3V4-20230911-21-Y25 | F8 | AGTTACGG | ACATTATC | 11.5381612 | 1.300033839 | 15 |
| LC-P20230902002 | la23i11A117 | S28 | fecal | V3V4-20230911-22 | E4 | V3V4-20230911-22-Y26 | E4 | TGGCTATC | ATCACGAA | 14.41384235 | 1.040666301 | 15 |
| LC-P20221204036 | la22l07A410 | S29 | fecal | V3V4-20221207-40 | E1 | V3V4-20221207-40-Y62 | E1 | TCAGTTCG | CTTGCGTG | 15.05603048 | 0.996278536 | 15 |
| LC-P20221204036 | la22l07A409 | S30 | fecal | V3V4-20221207-40 | D1 | V3V4-20221207-40-Y62 | D1 | TCAGTTCG | TGGTTCGT | 19.34714446 | 0.775308213 | 15 |
| LC-P20230902002 | la23i11A123 | S31 | fecal | V3V4-20230911-22 | C5 | V3V4-20230911-22-Y26 | C5 | CGGTCATA | CATACACT | 15.66829845 | 0.957347095 | 15 |
| LC-P20230902002 | la23i11A124 | S32 | fecal | V3V4-20230911-22 | D5 | V3V4-20230911-22-Y26 | D5 | CGGTCATA | TTCTAGCT | 16.62863066 | 0.902058642 | 15 |
| LC-P20230902002 | la23i11A127 | S33 | fecal | V3V4-20230911-22 | G5 | V3V4-20230911-22-Y26 | G5 | CGGTCATA | CTATTAAG | 15.83839419 | 0.947065707 | 15 |
| LC-P20230902002 | la23i11A129 | S34 | fecal | V3V4-20230911-22 | A6 | V3V4-20230911-22-Y26 | A6 | GAAGGTTC | GTTGATAG | 9.727350275 | 1.542043781 | 15 |
| LC-P20231216041 | la23l19A345 | S35 | fecal | V3V4-20231219-123 | C6 | V3V4-20231219-123-Y29 | C6 | GAAGGTTC | GTAGAGCA | 10.39557608 | 1.442921478 | 15 |
| LC-P20230902002 | la23i11A133 | S36 | fecal | V3V4-20230911-22 | E6 | V3V4-20230911-22-Y26 | E6 | GAAGGTTC | ATCACGAA | 11.72597525 | 1.279211296 | 15 |
| LC-P20230902002 | la23i11A135 | S37 | fecal | V3V4-20230911-22 | G6 | V3V4-20230911-22-Y26 | G6 | GAAGGTTC | CTATTAAG | 14.67430145 | 1.022195165 | 15 |
| LC-P20231216041 | la23l19A346 | S38 | fecal | V3V4-20231219-123 | D6 | V3V4-20231219-123-Y29 | D6 | GAAGGTTC | GATACTGG | 12.66416926 | 1.184444056 | 15 |
| LC-P20230902002 | la23i11A137 | S39 | fecal | V3V4-20230911-22 | A7 | V3V4-20230911-22-Y26 | A7 | ATCTCGCT | GTTGATAG | 16.82353203 | 0.891608253 | 15 |
| LC-P20230902002 | la23i11A140 | S40 | fecal | V3V4-20230911-22 | D7 | V3V4-20230911-22-Y26 | D7 | ATCTCGCT | TTCTAGCT | 15.71790971 | 0.95432537 | 15 |
| LC-P20230902002 | la23i11A141 | S41 | fecal | V3V4-20230911-22 | E7 | V3V4-20230911-22-Y26 | E7 | ATCTCGCT | ATCACGAA | 17.29661082 | 0.867221918 | 15 |
| LC-P20230902002 | la23i11A142 | S42 | fecal | V3V4-20230911-22 | F7 | V3V4-20230911-22-Y26 | F7 | ATCTCGCT | GCGTAAGA | 0.73530972 | 20.39956713 | 15 |
| LC-P20230902002 | la23i11A143 | S43 | fecal | V3V4-20230911-22 | G7 | V3V4-20230911-22-Y26 | G7 | ATCTCGCT | CTATTAAG | 13.86634668 | 1.081755732 | 15 |
| LC-P20230902002 | la23i11A145 | S44 | fecal | V3V4-20230911-22 | A8 | V3V4-20230911-22-Y26 | A8 | AGTTACGG | GTTGATAG | 12.04136111 | 1.245706351 | 15 |
| LC-P20230902002 | la23i11A149 | S45 | fecal | V3V4-20230911-22 | E8 | V3V4-20230911-22-Y26 | E8 | AGTTACGG | ATCACGAA | 11.72597525 | 1.279211296 | 15 |
| LC-P20230902002 | la23i11A150 | S46 | fecal | V3V4-20230911-22 | F8 | V3V4-20230911-22-Y26 | F8 | AGTTACGG | GCGTAAGA | 1.374940585 | 10.90956232 | 15 |
| LC-P20230902002 | la23i11A155 | S47 | fecal | V3V4-20230911-22 | C9 | V3V4-20230911-22-Y26 | C9 | ATGTAAGA | CATACACT | 15.11903095 | 0.992127078 | 15 |
| LC-P20231216041 | la23l19A351 | S48 | fecal | V3V4-20231219-123 | A7 | V3V4-20231219-123-Y29 | A7 | ATCTCGCT | TAAGTGGC | 10.63885154 | 1.40992662 | 15 |
| LC-P20231216041 | la23l19A353 | S49 | fecal | V3V4-20231219-123 | C7 | V3V4-20231219-123-Y29 | C7 | ATCTCGCT | GTAGAGCA | 16.5229533 | 0.907828021 | 15 |
| LC-P20230902002 | la23i11A71 | S50 | fecal | V3V4-20230911-21 | G9 | V3V4-20230911-21-Y25 | G9 | ATGTAAGA | TGGAACAG | 2.262627742 | 6.629459952 | 15 |
| LC-P20230902002 | la23i11A76 | S51 | fecal | V3V4-20230911-21 | D10 | V3V4-20230911-21-Y25 | D10 | TGACTTCG | AGAGTAGA | 14.83553804 | 1.011085675 | 15 |
| LC-P20230902002 | la23i11A118 | S52 | fecal | V3V4-20230911-22 | F4 | V3V4-20230911-22-Y26 | F4 | TGGCTATC | GCGTAAGA | 0.882371664 | 16.99963928 | 15 |
| LC-P20230902002 | la23i11A120 | S53 | fecal | V3V4-20230911-22 | H4 | V3V4-20230911-22-Y26 | H4 | TGGCTATC | AAGGCTAT | 12.6800186 | 1.182963565 | 15 |
| LC-P20230902002 | la23i11A126 | S54 | fecal | V3V4-20230911-22 | F5 | V3V4-20230911-22-Y26 | F5 | CGGTCATA | GCGTAAGA | 1.102078665 | 13.61064366 | 15 |
| LC-P20230902002 | la23i11A128 | S55 | fecal | V3V4-20230911-22 | H5 | V3V4-20230911-22-Y26 | H5 | CGGTCATA | AAGGCTAT | 10.04090534 | 1.493889196 | 15 |
| LC-P20231216041 | la23l19A347 | S56 | fecal | V3V4-20231219-123 | E6 | V3V4-20231219-123-Y29 | E6 | GAAGGTTC | GCCTTGTT | 12.35760261 | 1.213827671 | 15 |
| LC-P20230902002 | la23i11A139 | S57 | fecal | V3V4-20230911-22 | C7 | V3V4-20230911-22-Y26 | C7 | ATCTCGCT | CATACACT | 15.79587026 | 0.949615295 | 15 |
| LC-P20230902002 | la23i11A146 | S58 | fecal | V3V4-20230911-22 | B8 | V3V4-20230911-22-Y26 | B8 | AGTTACGG | TCTCTCCG | 12.9715722 | 1.156374861 | 15 |
| LC-P20230902002 | la23i11A157 | S59 | fecal | V3V4-20230911-22 | E9 | V3V4-20230911-22-Y26 | E9 | ATGTAAGA | ATCACGAA | 13.13280879 | 1.142177598 | 15 |
| LC-P20231216041 | la23l19A350 | S60 | fecal | V3V4-20231219-123 | H6 | V3V4-20231219-123-Y29 | H6 | GAAGGTTC | GATCACCG | 8.856809297 | 1.693612168 | 15 |
| LC-P20231216041 | la23l19A352 | S61 | fecal | V3V4-20231219-123 | B7 | V3V4-20231219-123-Y29 | B7 | ATCTCGCT | ACTAGCCG | 9.972316319 | 1.50416408 | 15 |
| LC-P20231216041 | la23l19A354 | S62 | fecal | V3V4-20231219-123 | D7 | V3V4-20231219-123-Y29 | D7 | ATCTCGCT | GATACTGG | 13.94185992 | 1.075896622 | 15 |
| LC-P20230902002 | la23i11A59 | S63 | fecal | V3V4-20230911-21 | C8 | V3V4-20230911-21-Y25 | C8 | AGTTACGG | TATCCTCT | 12.41167371 | 1.208539666 | 15 |
| LC-P20230902002 | la23i11A60 | S64 | fecal | V3V4-20230911-21 | D8 | V3V4-20230911-21-Y25 | D8 | AGTTACGG | AGAGTAGA | 16.40183634 | 0.914531745 | 15 |
| LC-P20230902002 | la23i11A61 | S65 | fecal | V3V4-20230911-21 | E8 | V3V4-20230911-21-Y25 | E8 | AGTTACGG | TGCCTGGT | 13.02118346 | 1.151969024 | 15 |
| LC-P20230902002 | la23i11A63 | S66 | fecal | V3V4-20230911-21 | G8 | V3V4-20230911-21-Y25 | G8 | AGTTACGG | TGGAACAG | 2.097847491 | 7.150186114 | 15 |
| LC-P20230902002 | la23i11A64 | S67 | fecal | V3V4-20230911-21 | H8 | V3V4-20230911-21-Y25 | H8 | AGTTACGG | CCTTGTTA | 8.901677192 | 1.685075708 | 15 |
| LC-P20230902002 | la23i11A65 | S68 | fecal | V3V4-20230911-21 | A9 | V3V4-20230911-21-Y25 | A9 | ATGTAAGA | TAGATCGC | 3.079441672 | 4.871012865 | 15 |
| LC-P20230902002 | la23i11A66 | S69 | fecal | V3V4-20230911-21 | B9 | V3V4-20230911-21-Y25 | B9 | ATGTAAGA | CTACAAGA | 18.7814049 | 0.798662298 | 15 |
| LC-P20230902002 | la23i11A67 | S70 | fecal | V3V4-20230911-21 | C9 | V3V4-20230911-21-Y25 | C9 | ATGTAAGA | TATCCTCT | 16.2264251 | 0.924418034 | 15 |
| LC-P20230902002 | la23i11A68 | S71 | fecal | V3V4-20230911-21 | D9 | V3V4-20230911-21-Y25 | D9 | ATGTAAGA | AGAGTAGA | 17.20624745 | 0.871776373 | 15 |
| LC-P20230902002 | la23i11A69 | S72 | fecal | V3V4-20230911-21 | E9 | V3V4-20230911-21-Y25 | E9 | ATGTAAGA | TGCCTGGT | 16.3185603 | 0.919198736 | 15 |
| LC-P20230902002 | la23i11A70 | S73 | fecal | V3V4-20230911-21 | F9 | V3V4-20230911-21-Y25 | F9 | ATGTAAGA | ACATTATC | 12.46128497 | 1.20372819 | 15 |
| LC-P20230902002 | la23i11A72 | S74 | fecal | V3V4-20230911-21 | H9 | V3V4-20230911-21-Y25 | H9 | ATGTAAGA | CCTTGTTA | 10.01438684 | 1.497845074 | 15 |
| LC-P20230902002 | la23i11A73 | S75 | fecal | V3V4-20230911-21 | A10 | V3V4-20230911-21-Y25 | A10 | TGACTTCG | TAGATCGC | 3.490506383 | 4.297370741 | 15 |
| LC-P20230902002 | la23i11A74 | S76 | fecal | V3V4-20230911-21 | B10 | V3V4-20230911-21-Y25 | B10 | TGACTTCG | CTACAAGA | 15.53186749 | 0.965756372 | 15 |
| LC-P20230902002 | la23i11A75 | S77 | fecal | V3V4-20230911-21 | C10 | V3V4-20230911-21-Y25 | C10 | TGACTTCG | TATCCTCT | 15.77460829 | 0.950895244 | 15 |
| LC-P20230902002 | la23i11A77 | S78 | fecal | V3V4-20230911-21 | E10 | V3V4-20230911-21-Y25 | E10 | TGACTTCG | TGCCTGGT | 16.26186172 | 0.922403613 | 15 |
